# Antimicrobial use in Sweden during the COVID-19 pandemic: prescription fill and inpatient care requisition patterns

**DOI:** 10.1186/s12879-022-07405-3

**Published:** 2022-05-24

**Authors:** Aya Olivia Nakitanda, Pär Karlsson, Lukas Löfling, Carolyn E. Cesta, Ingvild Odsbu

**Affiliations:** 1 Centre for Pharmacoepidemiology, Department of Medicine Solna, Karolinska Institutet, Eugeniahemmet T4, Maria Aspmans gata 30A, 17176 Stockholm, Sweden; 2 Section for Epidemiology and Prevention, Department of Research, Cancer Registry of Norway, Oslo, Norway; 3 Department of Mental Disorders, Norwegian Institute of Public Health, Oslo, Norway

**Keywords:** Antimicrobials, Antimicrobial stewardship, Covid-19, Pandemic, Sweden

## Abstract

**Background:**

Increased and inappropriate antimicrobial use are the key drivers of the emergence of antimicrobial resistance, and there have been widespread concerns around potential antimicrobial misuse, overuse and their consequences during the COVID-19 pandemic. To better understand the impact of the pandemic on antimicrobial use, particularly in light of the resurgence of COVID-19 cases since the summer of 2020, we assessed trends in antimicrobial prescription fills and hospital requisitions in Sweden during 2020 against those of preceding years.

**Methods:**

We performed a descriptive study using population-based data from the Swedish Prescribed Drug Register and the Swedish e-Health Agency. The weekly number of prescriptions filled and the total volume sold to inpatient care institutions in defined daily doses (DDDs) per 1000 inhabitants for systemic antibacterials (Anatomical Therapeutic Chemical therapeutic subgroup J01 excluding J01XX), antimycotics (J02), antivirals (J05) and antiprotozoals (P01) were computed and evaluated from time series graphs. A time series linear regression with ordinary least squares (OLS) estimation was used to model 2015–2019 data and predict the expected number of prescriptions filled and volumes sold in DDDs per 1000 inhabitants during 2020 with 95% confidence limits.

**Results:**

From mid-March 2020, the weekly rate of antibiotic and antiprotozoal prescriptions filled plummeted to unprecedentedly low levels for the rest of the year; while unprecedentedly high numbers of antiviral prescriptions were filled weekly between mid-February and mid-March 2020. There was a net reduction in annual dispensing of antibiotics by 17%; of antiprotozoals by 21%; and of antivirals by 0.3% during 2020 compared to 2019. Inpatient care requisitions of antiprotozoals and antibiotics surged to 6-year highs during March 2020, resulting in a 127% increase in DDDs of antiprotozoals sold from 2019. The volume of antibiotics and antivirals sold to inpatient care institutions in 2020 decreased by 3% and 13% compared to 2019, respectively.

**Conclusions:**

The overall decline in antimicrobial prescriptions filled in Sweden during 2020 were in part, collateral dividends of the COVID-19 pandemic.

**Supplementary information:**

The online version contains supplementary material available at 10.1186/s12879-022-07405-3.

## Background

Effective treatment against the corona virus disease 2019 (COVID-19) causing severe acute respiratory syndrome coronavirus 2 (SARS-COV-2) is yet to be established. A novel virus, coupled with the lack of readily accessible testing and clinical guidelines resulted in widespread diagnostic and clinical uncertainty of COVID-19 in the first months following the declaration of the pandemic by the World Health Organization (WHO) on 11 March 2020 [1, 2]. The rapidly mounting pressure on health systems in many countries prompted proposals and eventual use of empirical treatments that included different antimicrobials in the regimen. Antimicrobial use during the pandemic has been compounded by the anticipated co-infections and secondary infections with bacterial or fungal pathogens based on experiences from previous coronavirus epidemics, and speculations grew about potential antimicrobial misuse, overuse and their consequences [3, 4]. The European Medicines Agency (EMA) reported among others, a growing shortage of antibiotics in the European Union (EU), driven by increased demand and disruption in supply chains [5]. Restrictions were also placed on the use of hydroxychloroquine/chloroquine-azithromycin combination therapy to clinical trials and emergency programmes [6]. To date, remdesivir and nirmatrelvir/ritonavir combination are the only antimicrobials authorized for use in the treatment of COVID-19 across the EU [7].

While Sweden’s overall public health response has been considered less restrictive without a full societal lockdown [8], measures targeting medication use closely followed those of the EMA. The Swedish Medical Products Agency issued early warnings of and instituted counter measures to foreseeable drug shortages [9, 10] and unrecommended antimicrobial use accordingly [11]. However, unprecedented dispensing of chloroquine and hydroxychloroquine had already been detected on one hand [12], and on the other, antibiotic prescription fills were reportedly significantly lower during this first wave of the pandemic compared to the same period in pre-pandemic years [13]. The dynamics of these earlier observations and the impact of the pandemic on the use of other antimicrobial agents are limited and warrant further evaluation, particularly in light of the resurgence of COVID-19 cases since the summer of 2020.

In this descriptive study, we assessed trends in antimicrobial prescription fills and hospital requisitions in Sweden during 2020 against those of preceding years, with the overall aim to better understand the impact of the COVID-19 pandemic on antimicrobial use in primary and secondary care settings.

## Methods

### Materials

Aggregated data on drugs dispensed at pharmacies from 1 January 2015 to 29 December 2020 were obtained from the Swedish National Board of Health and Welfare. This included the total number of prescriptions filled at pharmacies per week, for all anatomical main groups of the Anatomical Therapeutic Chemical (ATC) classification system [14]. This data was available at population level, by sex, age groups (0–19, 20–39, 40–59, 60–79, 80 + years) as well as geography (metropolitan: three most populous counties, non-metropolitan: all other counties), and to the ATC chemical subgroup (4th level).

For hospital requisitions, the total daily sales of drugs in defined daily doses (DDDs) from pharmacies to inpatient healthcare institutions were received from the Swedish eHealth Agency [15]. The data covered the period from 1 January 2015 to 29 December 2020, and included all ATC codes to the therapeutic subgroup (2nd level). One DDD is the assumed average maintenance dose per day for a drug used for its main indication in adults [16]. This data did not include information on age, sex or geography. As previously described by Karlsson et al. (2021), weeks were started on a Wednesday for both dispensed and requisition data to align with the Swedish Medical Products Agency ordinance to limit dispensing of prescription drugs by pharmacies, that was issued on Wednesday 1 April 2020 [13].

We defined antimicrobials as systemic antibacterials, antimycotics, antivirals and antiparasitics according to their corresponding ATC therapeutic subgroups (Additional file 1: Table S1). The ATC chemical subgroup J01XX which contains the urinary antiseptic, methenamine (J01XX05), was not included in our analyses of filled prescriptions but the data format did not allow for exclusion at ATC therapeutic subgroup for inpatient care requisitions. Systemic antimicrobials are not available over-the-counter (OTC) in Sweden, and OTC sales were therefore not considered.

ATC/DDD index 2021 was applied in all the data [14]. Total population at national level and for each stratum was downloaded from the Statistics Sweden website on 12 March 2021 [17].

### Statistical analyses

Antimicrobial use was computed as the total number of prescriptions filled per 1000 inhabitants by week, and in DDDs per 1000 inhabitants by week for inpatient care requisitions.

Overall yearly use were calculated, and crude trends were initially analysed using time series line graphs at ATC therapeutic subgroup level (2nd level). This was followed by a time series linear regression model with ordinary least squares (OLS) estimation of 2015–2019 data to predict the expected number of prescriptions filled or volumes of DDDs sold, per 1000 inhabitants in 2020 with 95% confidence limits (95% CLs) at ATC therapeutic then chemical subgroups. At this point, subgroups with invariably few prescriptions filled over the years i.e. less than 0.01 prescriptions per 1000 inhabitants weekly, a poor fit of the model as assessed visually and/or without statistically significant deviations in 2020 compared to the model were excluded from further analyses and interpretation. We modelled: linear change of drug use over time using the date as a continuous variable; seasonal patterns using week as a categorical variable; Easter holidays using a 4-tier categorical variable consisting of the week before Easter, the week of Easter, the week after Easter, and other weeks; as well as holidays other than Easter using a continuous variable for the number of working days per week. Easter is a week-long holiday in Sweden that runs through different weeks each year, so these weeks cannot be directly aligned for analysis. The number of working days took into consideration days when pharmacies are closed. To understand the drivers and intensity of the deviations in 2020, the actual observed weekly values for 2020 were then compared against those predicted by this model. Bonferroni correction was applied by dividing the standard significance limits by 52, the number of weeks in 2020. This addressed the multiplicity in weekly comparisons within drug groups.

All statistical analyses were performed with SAS version 9.4 and SAS/STAT 14.3.

## Results

### Prescription fills

**Fig. 1:**
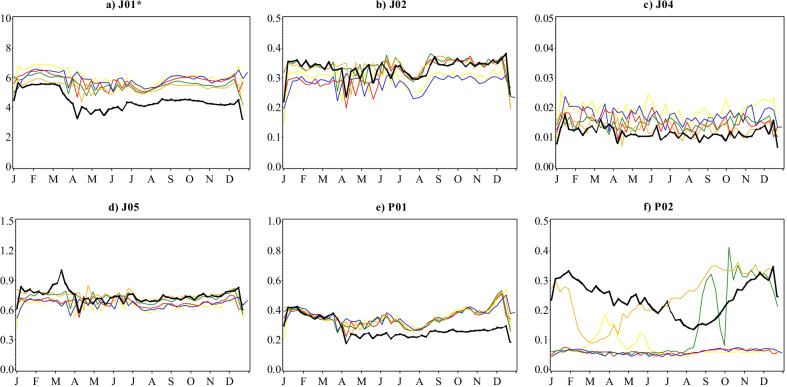
Weekly number of antimicrobial prescriptions filled per 1000 inhabitants by ATC therapeutic subgroup, Sweden, 2015–2020. Legend:Yellow-2015; Blue-2016; Red-2017; Green-2018; Orange-2019; Black-2020. Note that the scales on the y-axes are different and not directly comparable. *Excluding J01XX

**Fig. 2:**
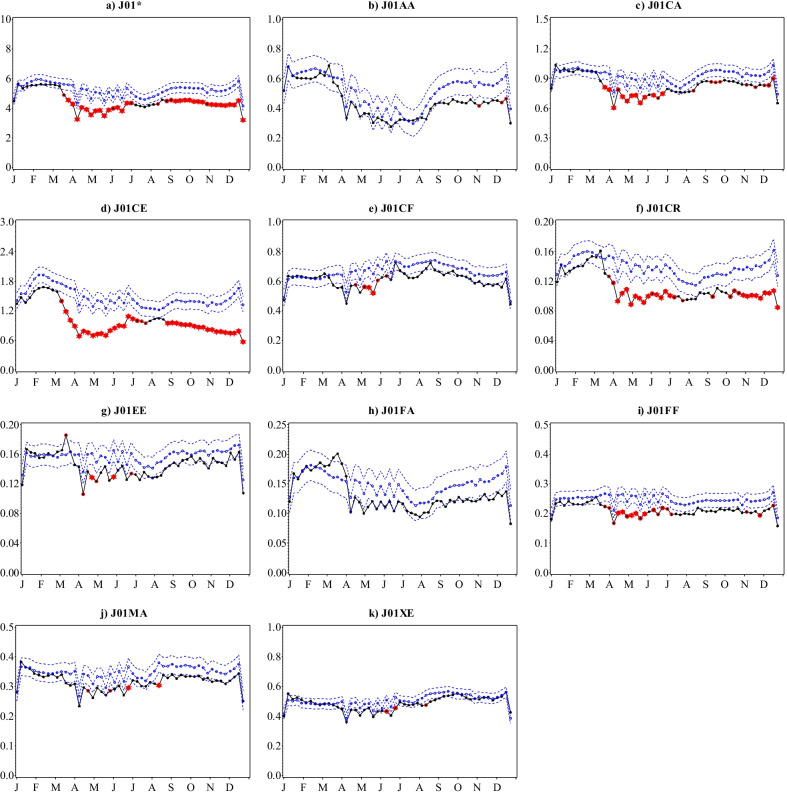
Observed versus predicted weekly number of antibacterial prescriptions filled per 1000 inhabitants with 95% confidence limits by ATC therapeutic subgroup and selected chemical subgroups, Sweden, 2020. Legend: Black-observed; Blue dashed dotted-predicted; Blue dashed-95% confidence limits. Values that remained significant after Bonferroni correction are marked as follows: Single red circle: p ≤ 0.05; Double red circle: p ≤ 0.01; Red star: p ≤ 0.001. Note that the scales on the y-axes are different and not directly comparable. *Excluding J01XX

The steady annual decrease in dispensing of antibacterials (J01) in Sweden between 2015 and 2019 from 314 to 279 prescriptions filled per 1000 inhabitants continued into 2020 reaching 232 prescriptions filled per 1000 inhabitants (Fig. 1a, Additional file 1: Table S1). This represented a decrease of 17% in 2020 from 2019 compared to the annual decline of 0.3–4.0% recorded between 2015 and 2019. While prescription fill patterns in 2020 followed the overall seasonality of preceding years, an earlier descent commenced in the second week of March reaching an all-time low for the 6-year reporting period in the first week of April, then remained at unprecedentedly low levels for the rest of the year. Statistically significant deviations from predicted estimates in 2020 were observed in the weekly number of prescriptions filled for penicillins with extended spectrum (J01CA); beta-lactamase sensitive penicillins (J01CE); beta-lactamase resistant penicillins (J01CF); combinations of penicillins, including beta-lactamase inhibitors (J01CR); combinations of sulfonamides and trimethoprim, including derivatives (J01EE); lincosamides (J01FF); and floroquinolones (J01MA) (Fig. 2).

**Fig. 3:**
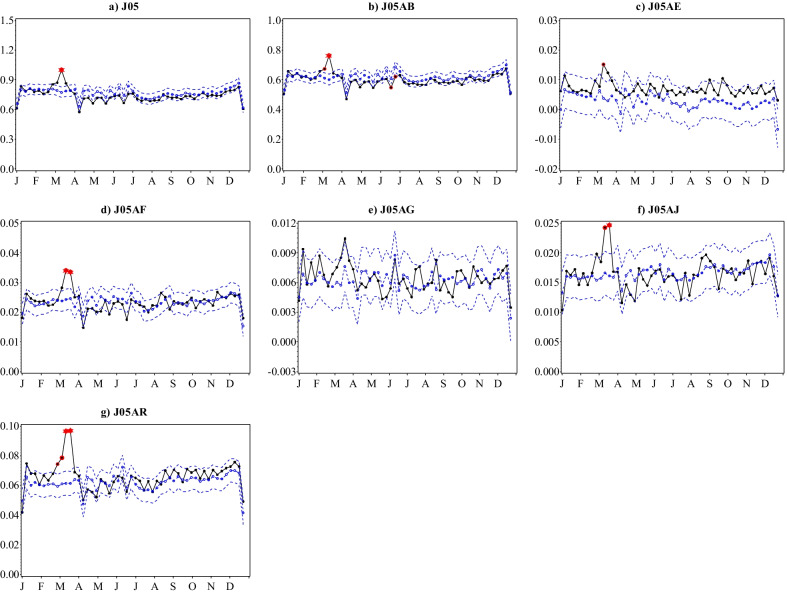
Observed versus predicted weekly number of antiviral prescriptions filled per 1000 inhabitants with 95% confidence limits by ATC therapeutic and selected chemical subgroups, Sweden, 2020. Legend: Black-observed; Blue dashed dotted-predicted; Blue dashed-95% confidence limits. Values that remained significant after Bonferroni correction are marked as follows: Single red circle:  p ≤ 0.05; Double red circle: p ≤ 0.01; Red star: p ≤ 0.001. Note that the scales on the y-axes are different and not directly comparable

For antivirals (J05), the steady increase in the total number of prescriptions filled between 2015 and 2019 carried on into the first quarter of 2020. However, the number of prescriptions filled soared from the third week of February 2020 reaching a peak in mid-March (Fig. 1d). This was followed by a sharp decline over the next two weeks, and a return to patterns closely aligned with previous years for the rest of 2020. The total number of antiviral prescriptions filled per 1000 inhabitants during 2020 decreased by 0.3% to 38.7 from 38.8 in 2019 overall, contrary to consistent annual increases of up to 6.8% registered between 2015 and 2019 (Additional file 1: Table S1). The ascending trajectory that commenced in the last week of February and reached an all-time high for the 2015–2020 period in the 2nd and 3rd weeks of March 2020 was observed for all frequently dispensed ATC chemical subgroups dispensed except non-nucleoside reverse transcriptase inhibitors (NNRTIs, J05AG) (Fig. 3), reaching peaks in March that were significantly higher than predicted. These increases were most apparent for nucleosides and nucleotides excluding reverse transcriptase inhibitors (J05AB), nucleoside and nucleotide reverse transcriptase inhibitors (NRTIs, J05AF), integrase inhibitors (J05AJ) and antivirals for treatment of HIV infections, combinations (J05AR). Following the sharp decline in the ensuing weeks, minor deviations from predicted values occurred among all antiviral ATC chemical subgroups for the rest of the year.

**Fig. 4:**
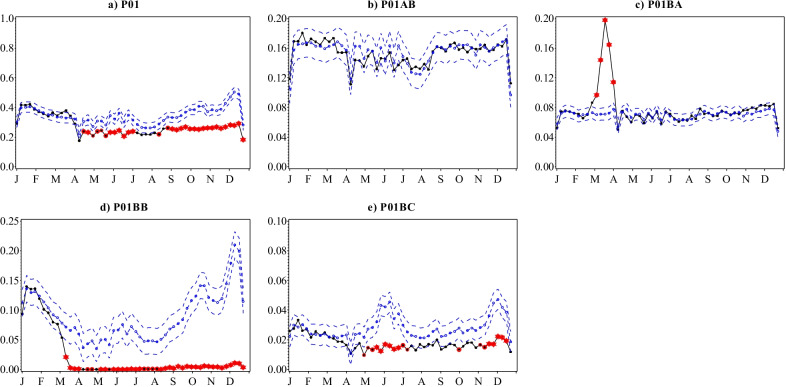
Observed versus predicted weekly number of antiprotozoal prescriptions filled per 1000 inhabitants with 95% confidence limits by ATC therapeutic and selected chemical subgroups, Sweden, 2020. Legend: Black-observed; Blue dashed dotted-predicted; Blue dashed-95% confidence limits. Values that remained significant after Bonferroni correction are marked as follows: Single red circle: p ≤ 0.05; Double red circle: p ≤ 0.01; Red star: p ≤ 0.001. Note that the scales on the y-axes are different and not directly comparable

The number of antiprotozoal (P01) prescriptions filled in Sweden had been stable and followed distinct seasonality through 2015 until February 2020 (Fig. 1e), with 18–19 prescriptions annually filled per 1000 inhabitants in the 2015–2019 period. A modest increase in antiprotozoal prescription fills followed from mid-February that peaked in the 2nd week of March, before plummeting to unprecedentedly low prescription fill rates for the rest of 2020. Compared to the total number of antiprotozoal prescriptions filled for 2019, the 14 prescriptions filled per 1000 inhabitants in 2020 was a 21% decrease (Additional file 1: Table S1). Significantly higher numbers of prescriptions per 1000 inhabitants were filled than predicted for the aminoquinolines (P01BA) during a 5-week period between the last week of February and until the end of March 2020 (Fig. 4). By the 2nd week of April, weekly trends had descended to seasonal patterns which were sustained for the rest of the year. Conversely, biguanides (P01BB) prescriptions filled in Sweden plummeted from early February to virtually no prescriptions filled from the 3rd week of March 2020. Prescription fill rates for methanolquinolines (P01BC) also remained significantly lower than predicted for the most part of the year from May 2020. For nitroimidazole derivatives (P01AB), prescription fill trends in 2020 remained largely within prediction.

The total number of antimycotic (J02) prescriptions filled has largely remained low, ranging from 15 to 18 per 1000 inhabitants annually in 2015 and 2020 respectively, with comparable seasonality over the entire reporting period of the study (Fig. 1b). Trends in the total number of antimycobacterial (J04) prescriptions filled annually remained low and continued to decrease into 2020, from 1.0 to 0.6 prescriptions per 1000 inhabitants over the 6 years (Fig. 1c). Anthelmintics (P02) use in Sweden had been stable averaging 3.3 prescriptions filled per 1000 inhabitants with consistent seasonality for the most part (Fig. 1f). Since August 2018 when a marked increase was observed, the total number of prescriptions filled annually have been relatively higher (7.2–13 per 1000 inhabitants) with irregular patterns.

**Fig. 5:**
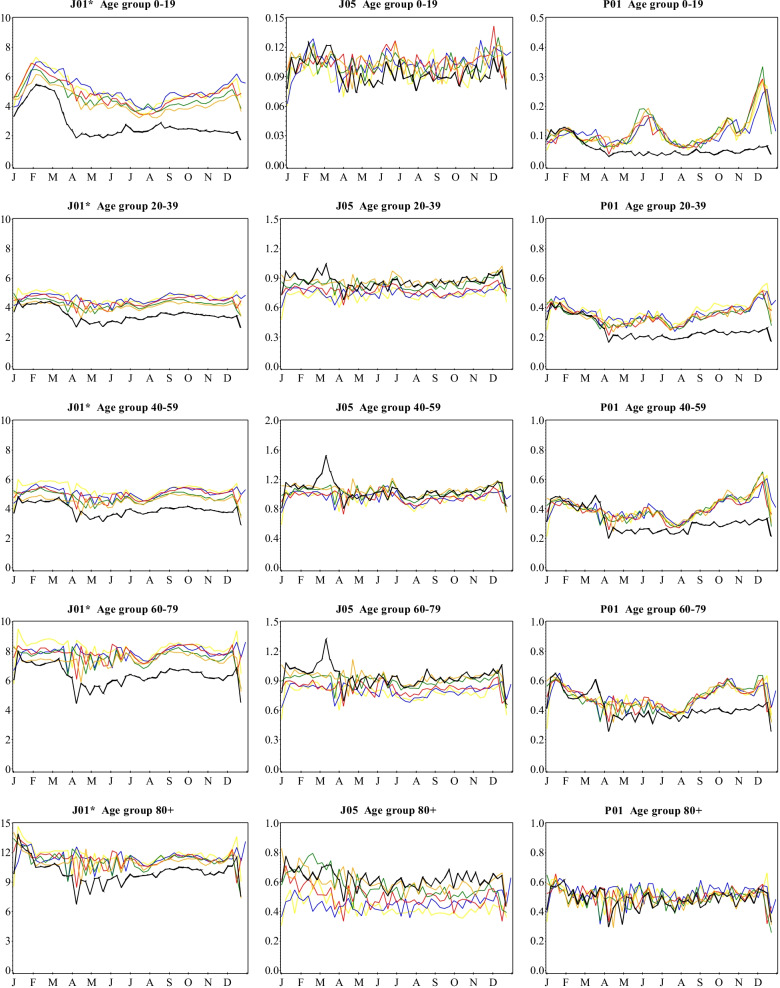
Weekly number of antimicrobial prescriptions filled per 1000 inhabitants for selected ATC therapeutic subgroups, by age group, Sweden, 2015–2020. Legend: Yellow-2015; Blue-2016; Red-2017; Green-2018; Orange-2019; Black-2020. Note that the scales on the y-axes are different and not directly comparable. *Excluding J01XX

Demographically, the elderly (≥ 60 years) population have consistently filled more antibacterial, antiviral and antiprotozoal prescriptions over the 6-year reporting period (Fig. 5). Women than men as well as those registered as living in metropolitan than other areas, have also filled more prescriptions overall between 2015 and 2020 (Additional file 2: Figs. S1–S4). The overall decline in the number of antibacterial prescriptions filled from mid-March 2020 for the rest of the year was most apparent among those aged 0–19 years, while the increase in antiviral prescription fills between late February and mid-March was observed among the population aged 20–39, 40–59 and 60–79 years (Fig. 5). For both antibacterials and antivirals, the observed deviations from preceding years did not, however, differ considerably by sex or geographical area (Additional file 2:  Fig. S1). During March 2020, the surge in antiprotozoal prescription fills occurred only in the 40–59 and 60–79 age groups (Fig. 5), and was more apparent among men than women as well as in metropolitan than non-metropolitan areas. The subsequent decline and unprecedentedly lower number of antiprotozoal prescriptions filled from April 2020 tended to be more overt in the younger population (< 60 years), among men and those registered as living in metropolitan areas (Fig. 5).

### Inpatient care requisitions

**Fig. 6:**
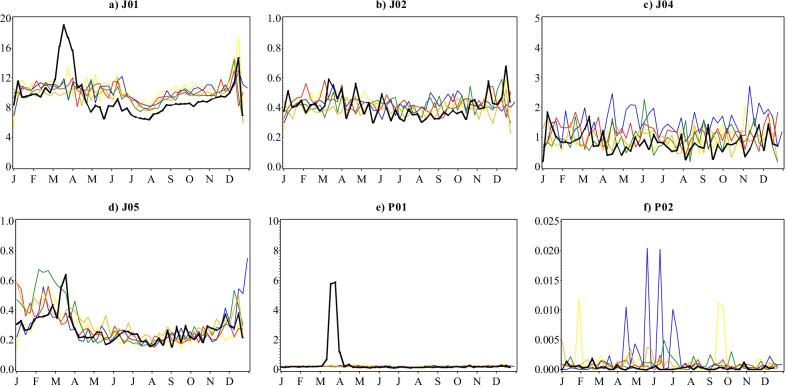
Weekly volumes of inpatient care antimicrobial requisitions in DDDs per 1000 inhabitants by ATC therapeutic subgroup, Sweden, 2015–2020. Legend: Yellow-2015; Blue-2016; Red-2017; Green-2018; Orange-2019; Black-2020. Note that the scales on the y-axes are different and not directly comparable

**Fig. 7:**
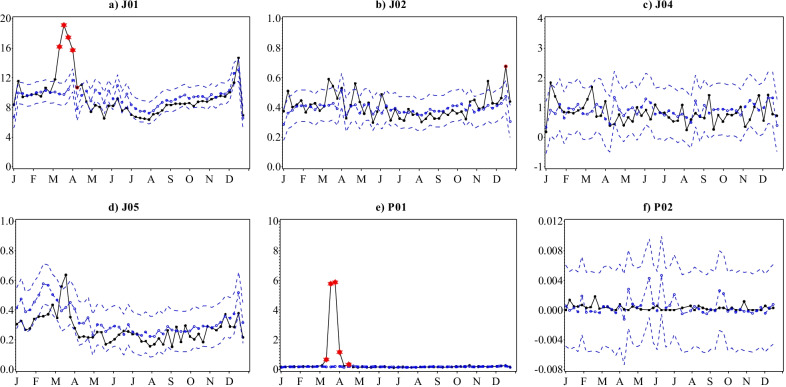
Observed versus predicted weekly volumes of inpatient care antimicrobial requisitions in DDDs per 1000 population with 95% confidence limits by ATC therapeutic subgroup, Sweden, 2020. Legend: Black-observed; Blue dashed dotted-predicted; Blue dashed-95% confidence limits. Values that remained significant after Bonferroni correction are marked as follows: Single red circle: p ≤ 0.05; Double red circle: p ≤ 0.01; Red star: p ≤ 0.001. Note that the scales on the y-axes are different and not directly comparable

As for the trends in antibacterial prescription fills, there has been a steady decline in the yearly inpatient care requisitions of antibacterials (J01) over the reporting period (Fig. 6a) from 553 to 492 DDDs per 1000 inhabitants in 2015 and 2020, respectively. However, antibacterial requisitions soared to 6-year highs during March 2020, remaining significantly higher than predicted until the 2nd week of April (Fig. 7a). For the rest of the year, values were largely lower than the preceding five years and reached 6-year lows, but seasonality were aligned to previous years and values not significantly lower than predicted. Summing up, antibacterial requisitions decreased by 3.0% in 2020 from 2019. Antiprotozoals (P01) on the other hand, had maintained a consistently low total volume of requisitions of 11 to 9.7 DDDs per 1000 inhabitants yearly between 2015 and 2019 (Fig. 6e). Similar to antibacterials, requisitions rose to record highs during March before returning to the predicted seasonal patterns from April 2020 (Fig. 7e). At its peak in mid-March 2020, inpatient care requisitions were almost twice the model estimate and contributed to an overall 127% excess in total requisitions compared to 2019.

No statistically significant deviations from predicted patterns were observed in the volumes of antivirals (J05), antimycotics (J02), antimycobacterials (J04) and anthelmintics (P02) requisitioned by inpatient care institutions for 2020 (Fig. 7b–d, f). Although an isolated surge in antiviral requisitions occurred during March 2020 peaking at 0.6 DDDs per 1000 inhabitants in the 3rd week, weekly volumes over the month were within the limits predicted by the model and otherwise followed seasonal patterns from previous years for the rest of the year (Figs. 6d and 7d). Moreover, despite the overall increasing trend in the total volume of antiviral requisitions between 2015 and 2019 from 13 to 16 DDDs per 1000 inhabitants, the total volume of 14.1 DDDs per 1000 inhabitants requisitioned in 2020 was a 13% decline from 2019. The total inpatient requisitions of both antimycotics and antimycobacterials maintained a stable low and non-deviant seasonality throughout the entire 2015–2020 reporting period (Figs. 6b and c and 7b and c) registering 22–21 and 51–43 DDDs per 1000 inhabitants, respectively. Inpatient care anthelmintics requisitions have also remained low over the years i.e. < 0.02 DDDs per 1000 inhabitants per week, except for sporadic sales, and remained so during 2020.

## Discussion

In assessing trends in prescription fills and inpatient care requisitions, the present study found that the number of antibiotics, antiviral and antiprotozoal prescriptions filled at pharmacies in Sweden registered significant shifts from predicted estimates in the days leading to, and in the aftermath of, the WHO’s declaration of the COVID-19 pandemic on 11 March 2020. From mid-March 2020, the weekly number of antibiotic and antiprotozoal prescriptions filled plummeted to unprecedentedly low levels for the rest of the year; while unprecedentedly high numbers of antiviral prescriptions were filled between mid-February and mid-March 2020. Overall, there were net reductions in the yearly dispensing of antibiotics, antiprotozoals and antivirals during 2020 from 2019. At the same time, inpatient care requisitions of antiprotozoals and antibiotics surged to 6-year highs during the month of March. This led to excess antiprotozoals requisitions and menial reduction of antibiotic requisitions in 2020 compared to 2019. The volume of antivirals sold to inpatient care institutions also decreased in 2020 overall compared to 2019.

The first confirmed case of COVID-19 was detected in Sweden on 31 January 2020 and following the diagnosis of the second, the Swedish National Board of Health and Welfare issued a high alert on 26 February 2020. A myriad of public health responses were launched in the ensuing weeks, but it was not until late May 2020 that WHO first released clinical guidelines [1], with the first Swedish clinical guideline following suit in late June 2020 [18]. Among the antimicrobial agents initially proposed as potential treatments against SARS-CoV-2 infection were the aminoquinolines (P01BA): hydroxychloroquine and chloroquine, and our results corroborate earlier reports from Swedish authorities that these prescriptions had been filled at unprecedented levels during March 2020. Notably, macrolides (J01FA) which includes azithromycin, used in combination with the aminoquinolines, followed the same trend during this period. Prescription fills had dropped markedly by the 2nd week of April, which corresponds to the period soon after EMA and the Swedish Medical Products Agency issued restrictions for the use of hydroxychloroquine/chloroquine and azithromycin combination therapy to clinical trials [6, 11], with Sweden further reinforcing this restriction with a law limiting prescribing of hydroxychloroquine and chloroquine to certain specialists which remained in force until 31 October 2020 [19].

According to the Swedish national statistical database of drugs that contains data on all prescriptions redeemed at pharmacies, hydroxychloroquine and chloroquine are the only aminoquinolines (P01BA) that have been dispensed by Swedish pharmacies between 2015 and 2020 [20]. Both drugs are otherwise indicated for rheumatoid arthritis, polymorphic light eczema, systemic and discoid lupus erythematosus in Sweden [21], for which recent changes in disease burden have not been reported in the literature. Although the surges observed in 2020 are thus likely to be in relation to the COVID-19 pandemic, this is not reflected in the number of confirmed cases during the month of March 2020 owing to the low coverage of testing particularly outside hospital settings at that time [8, 22]. Clinicians may have prescribed them out of vigilance considering the uncertainties of the clinical course amidst a lack of clinical guidelines, or were driven by the undetected surge in community transmission. The latter is supported by the inclination in prescription filling of these antimicrobials by men aged 40–79 years living in metropolitan areas, which reflects the epidemiological situation of COVID-19 at that time i.e. older males presenting with more severe forms of disease in metropolitan areas like Stockholm, where disease transmission was higher [22]. In the latter part of 2020, ivermectin (P02CF01) was also proposed as potential treatment for COVID-19 in some countries. However, our analyses detected no increased dispensing in relation to the pandemic.

By the end of 2020, about 455,000 confirmed cases, over 4000 intensive care unit (ICU) admissions and almost 10,000 fatalities in connection with SARS-CoV-2 infection had been recorded in Sweden [22]. Between March and May 2020, about 20% of adult patients admitted in ICUs with COVID-19 had been treated with hydroxychloroquine or chloroquine, and less than 1% with remdesivir or lopinavir-ritonavir combination as COVID-19 specific antimicrobial pharmacotherapy [23]. Earlier in March, majority received hydroxychloroquine and chloroquine before remdesivir or lopinavir-ritonavir were used [23]. This could in part explain the sudden increase in the volume of antiprotozoals (P01) sold to inpatient care institutions during March, as well as the corresponding decline in April in line with the EMA and Swedish Medical Products Agency guidelines issued on the use of hydroxychloroquine and chloroquine. Remdesivir was only approved for use in the EU in late June 2020 and for the treatment of COVID patients from 12 years of age with pneumonia who require supplemental oxygen [24–26]. At the same time, our dataset was based on the ATC/DDD version 2021 which does not contain the temporary ATC code for remdesivir (J05AB16) [25]. Although remedisivir remained the only antimicrobial approved for COVID-19 treatment for the rest of 2020, a potential increase in the inpatient care requisitions of antivirals which could have occurred following its approval and roll out was therefore not observed in our data. The isolated increase in antiviral requisitions during March 2020 could, therefore, be attributed to the increased demand for lopinavir-ritonavir combination (J05AR10) in inpatient care, but this could not be verified from our dataset nor the statistical database for drugs [19].

Although bacterial or fungal co-infections occurs in about 8–9% of hospitalized COVID-19 patients [27, 28], a recent systematic review estimated that 75% of patients with COVID receive antibiotics [28]. Our analyses did indicate significantly higher volumes in inpatient care requisitions of antibiotics in the month of March, but there were no reports pertaining to the use of antibiotics or antifungals for the management of bacterial and fungal co-infections and secondary infections in Sweden. Owing to the increased workload and pressure placed on hospitals as a result of the pandemic, elective surgeries were cancelled and therefore prophylactic use of antibiotics decreased [29]. The modest overall decrease in requisitions for 2020, decline in prophylactic use and a return to predicted trends rather than lower requisitions for the rest of the year imply that antibiotics could have been used for COVID-19 inpatients. However, the antibiotic use in 2020 and 2019 were comparable relative to patient stay in hospitals [29].

In relation to antibiotic prescriptions filled during 2020, the present study confirms and builds on the Swedish Public Health Agency’s earliest reports of outstanding decreases particularly among antibiotics used to treat respiratory tract infections (RTIs), urinary tract infections (UTIs) and skin infections [30]. Marked reductions were seen for penicillins with extended spectrum (J01CA); beta-lactamase resistant penicillins (J01CF); and combinations of penicillins, including beta-lactamase inhibitors (J01CR); that are largely indicated for the treatment of these common infections [31]. Pivmecillinam (J01CA08) and amoxicillin (J01CA04) the most frequently filled J01CA prescriptions in Sweden during the reporting period [19], are primarily used to treat uncomplicated UTIs and RTIs, respectively. Flucloxacillin (J01CF05) represents almost all beta-lactamase resistant penicillins (J01CF) dispensed in Sweden [19] and is used to treat skin infections. Amoxicillin and beta-lactamase inhibitor combination therapies (J01CR02) such as amoxicillin-clavulanic acid is used for a range of infections from RTIs, UTIs to skin infections.

Notably, combinations of sulfonamides and trimethoprim, including derivatives (J01EE); and macrolides (J01FA) chemical subgroups prescription fills increased briefly beyond the predicted limits during the first 2 weeks of March before plummeting. While we speculated macrolides (J01FA) increases to be in relation to hydroxychloroquine and chloroquine dispensing, the former (J01EE) includes sulfamethoxazole and trimethoprim (J01EE01) [19] which is not only indicated for treatment of UTIs, but also RTIs. The other ATC chemical subgroups that followed the same trends but to a lesser extent and within prediction limits: tetracyclines (J01AA), beta-lactamase resistant penicillins (J01CF) and combinations of penicillins, including beta-lactamase inhibitors (J01CR) may also be indicated for RTIs further raising speculations of a COVID-19 related increase in the community burden of RTIs during this period. Parallel to this increased dispensing of respiratory antibiotics, the largest increase in prescription fills of drugs targeting the respiratory system i.e. ATC anatomical group R, particularly drugs for obstructive airway diseases (R03) like asthma, occurred during March 2020 [13].

Subsequently, trends in antibiotics used to treat common infections remained unprecedentedly low for the rest of 2020. Dispensing of broader spectrum antibiotics such as other beta-lactam antibacterials (J01D) and quinolones (J01M) also reached significantly lower than predicted levels. Similar declines in antibiotic prescriptions filled during 2020 have been reported in Denmark (17% less than 2010–2019) and Norway, with marked reductions in those indicated for RTIs, UTIs and among younger age groups [32, 33]. As in Denmark and Norway, the Swedish Public Health Agency attributes these to improved infection control practices including personal hygiene, reduced physical contact in the society but also diminished contact with healthcare settings in light of advice to refrain from unnecessary healthcare visits or fear of acquiring/transmitting infections, among potential determinants [30]. Sweden has been a global leader in antimicrobial stewardship [34] with consistently low levels of antibiotic consumption [35] and resistance [36] in the pre-pandemic era, but the long-held Swedish national annual target of 250 prescriptions per 1000 inhabitants was achieved in 2020 [29].

The surge in antiviral prescription fills that occurred between late February and mid-March 2020, was largely driven by nucleosides and nucleotides excluding reverse transcriptase inhibitors (J05AB), NRTIs (J05AF), integrase inhibitors (J05AJ) and antivirals for treatment of HIV infections, combinations (J05AR); and to a lesser extent by protease inhibitors (J05AE) and NNRTIs (J05AG). Of these, J05AR, J05AF, J05AE, J05AG and J05AJ constitute antiretroviral therapies for HIV and chronic hepatitis B virus (HBV) infection. J05AB alike, are indicated for herpes simplex and herpes zoster infections, also chronic viral infections. Lopinavir-ritonavir combination (J05AR10) therapy was used to treat COVID-19 inpatients in Sweden before April 2020, and whereas our analyses detected profound increases in prescription fills for J05AR during February and March alongside other antimicrobial therapies proposed for the treatment of COVID-19, the overall number of prescriptions for 2020 was not considerably increased [19]. Thus, it is more likely that patients with chronic communicable diseases sought prescription refills in anticipation of restrictions. Such phenomena have also been reported in Sweden with drugs indicated for chronic respiratory, cardiovascular and psychiatric conditions, which were particularly vulnerable to potential stockpiling during the same period of the pandemic [13]. Similarly, stockpiling of aminoquinolines (P01BA) for its conventional indications of chronic inflammatory conditions could thus be postulated.

Despite these early surges in the year, the total number of antiviral prescriptions filled registered a small yet noteworthy decline in 2020, contrary to consistent increases recorded in preceding years. The overall reduction in inpatient care requisitions of antivirals was even larger, but this data did not include remdesivir. The decline in prescription fills could be explained by the impact of the pandemic on healthcare services, social and economic disruptions that affected case detection and linkage to care. For chronic hepatitis C virus (HCV) infection, the number of patients diagnosed and initiated on treatment between January and October 2020 reduced by 27% and 55%, respectively, compared to the same period in 2019 [37, 38]. Further, reduced transmission of chronic viral infections and subsequent decreased demand for prescribed antiviral drugs could also have been influenced by the decreased physical contact, travel to and immigration from endemic countries in relation to COVID-19 mandated restrictions. The incidence of HIV infections in 2020, for example, has been reported to be lowest since 2002, with declines for infections contracted both within Sweden and abroad [38]. Put together, these reductions albeit minimal, are suggestive of not only reduced disease transmission but also potential setbacks for HIV, HBV and HCV elimination programmes.

Although aminoquinoline (P01BA) prescriptions were filled at significantly high levels, a parallel decrease in fills of biguanides (P01BB) resulted in a minor net increase in dispensing of antiprotozoals during March. Biguanide combinations (P01BB51) [39] is the only biguanide (P01BB) that has been dispensed at pharmacies in Sweden between 2015 and 2020 [19] and is likely to be the malaria prophylaxis atovaquone-proguanil combination. While the Swedish Ministry of Foreign Affairs recommended against non-essential travel on  14 March 2020 [40], our data showed virtually no prescriptions filled by this date and for the rest of the year, indicating diminished travel to malaria endemic areas, the majority of which are located outside the EU where travel was particularly restricted.

### Strengths and limitations

The data was population-based with comprehensive national coverage and of high overall quality [41, 42]. We also presented previously unreported data covering a broad range of antimicrobials and the second half of 2020 corresponding to the second wave of the pandemic. While our dataset did not have data at substance level, we utilized publicly available national prescriptions data in the Swedish National Board of Health and Welfare’s online statistical database for drugs to aid the interpretation of our findings [19]. However, some limitations remained. Although filled prescription data mitigates primary non-compliance and requisitions may reflect the anticipated needs of hospitals, we acknowledge that they do not represent actual use. The indications for which the antimicrobials were prescribed for, or requisitioned for use in hospitals, were not available in the datasets. In addition, we did not have individual level data on filled prescriptions, limiting us in delineating between incident and prevalent use.

## Conclusions

Contrary to speculations and reports of exponential trends in antibiotic use during the COVID-19 pandemic in other countries [28], Sweden registered overall reductions in the rates of antibiotic, antiviral and antiprotozoal prescriptions filled at pharmacies in 2020 despite maintaining a non-conventional public health response towards the pandemic. Antimicrobial use in Sweden was not negatively impacted beyond the early months of the COVID-19 pandemic in Sweden and reductions could be partly attributed as collateral dividends at par with countries that implemented more restrictive measures.

## Supplementary Information

**Additional file 1: Table S1.** Total number of prescriptions filled per1000 inhabitants for selected ATC therapeutic and chemical subgroups by year, Sweden, 2015-2020. **Table S2.** Total number of antimicrobial prescriptions filled per 1000 inhabitants by ATC therapeutic subgroup, age group and year, Sweden, 2015-2020. **Table S3.** Total number of antimicrobial prescriptions filled per 1000 inhabitants, by ATC therapeutic subgroup, sex and year, Sweden, 2015-2020. **Table S4.** Total number of antimicrobial prescriptions filled per 1000 inhabitants, by ATC therapeutic subgroup, geography and year, Sweden, 2015-2020. **Table S5.** Observed versus predicted weekly number of prescriptions filled per 1000 inhabitants for selected ATC therapeutic and chemical subgroups, Sweden, 2020. **Table S6.** Total volumes of antimicrobials sold to inpatient care institutions in DDDs per 1000 inhabitants, by ATC therapeutic subgroup and year, Sweden, 2015-2020. **Table S7.** Observed versus predicted weekly volumes of antimicrobials sold per 1000 inhabitants by ATC therapeutic subgroup, Sweden, 2020.

**Additional file 2: Figure S1.** Weekly number of prescriptions filled per 1000 inhabitants for selected ATC therapeutic subgroups, by gender, Sweden, 2015-2020 Legend: Yellow-2015;Blue-2016; Red-2017; Green-2018; Orange-2019; Black-2020. Note that the scales on the y-axes are different and not directly comparable. *Excluding J01XX. **Figure S2.** Weekly number of prescriptions filled per 1000 inhabitants for selected ATC therapeutic subgroups, by geographical area, Sweden, 2015-2020. Legend: Yellow-2015; Blue-2016; Red-2017; Green-2018; Orange-2019; Black-2020. Note that the scales on the y-axes are different and not directly comparable. *Excluding J01XX. **Figure S3.** Observed versus predicted weekly number of prescriptions filled per 1000 inhabitants with 95% CL for selected antimicrobials by ATC therapeutic subgroup, Sweden, 2020. Note that the scales on the y-axes are different and not directly comparable. Legend: Black–observed; Blue dashed dotted–predicted; Blue dashed-95% confidence limits. Values that remained significant after Bonferroni correction are marked as follows: Single red circle– p≤0.05; Double red circle–p≤0.01;Red star-p≤0.001. **Figure S4.** Observed versus predicted weekly number of prescriptions filled per 1000 inhabitants with 95% CL for other antimicrobials by ATC chemical subgroups, Sweden, 2020. Note that the scales on they-axes are different and not directly comparable. Legend: Black-observed; Blue dashed dotted-predicted; Blue dashed-95% confidence limits. Values that remained significant after Bonferroni correction are marked as follows: p≤0.05 – single red circle;p≤0.01 – double red circle;p≤0.001 - red star.

## Data Availability

We provided links to the websites of the Swedish National Board of Health and the Swedish eHealth Agency.
