# Supplementary material for: Antimicrobial use in Sweden during the COVID-19 pandemic: prescription fill and inpatient care requisition patterns
Source: BMC Infect Dis. 2022 May 24;22:492. doi: 10.1186/s12879-022-07405-3 (PMC9128331; doi:10.1186/s12879-022-07405-3)
Supplement: Supplementary file 1 — Additional file 1: Table S1. Total number of prescriptions filled per1000 inhabitants for selected ATC therapeutic and chemical subgroups by year, Sweden, 2015-2020. Table S2. Total number of antimicrobial prescriptions filled per 1000 inhabitants by ATC therapeutic subgroup, age group and year, Sweden, 2015-2020. Table S3. Total number of antimicrobial prescriptions filled per 1000 inhabitants, by ATC therapeutic subgroup, sex and year, Sweden, 2015-2020. Table S4. Total number of antimicrobial prescriptions filled per 1000 inhabitants, by ATC therapeutic subgroup, geography and year, Sweden, 2015-2020. Table S5. Observed versus predicted weekly number of prescriptions filled per 1000 inhabitants for selected ATC therapeutic and chemical subgroups, Sweden, 2020. Table S6. Total volumes of antimicrobials sold to inpatient care institutions in DDDs per 1000 inhabitants, by ATC therapeutic subgroup and year, Sweden, 2015-2020. Table S7. Observed versus predicted weekly volumes of antimicrobials sold per 1000 inhabitants by ATC therapeutic subgroup, Sweden, 2020. [file 12879_2022_7405_MOESM1_ESM.docx]

Table S1 Total number of prescriptions filled per 1000 inhabitants for selected ATC therapeutic and chemical subgroups by year, Sweden, 2015-2020

|  | | Prescriptions filled per 1000 inhabitants, N | | | | | |
| --- | --- | --- | --- | --- | --- | --- | --- |
|  | | 2015 | 2016 | 2017 | 2018 | 2019 | 2020 |
| J01 excluding J01XX | Antibacterials for systemic use | 313.7 | 312.7 | 300.6 | 288.6 | 279.4 | 231.5 |
| J01AA | Tetracyclines | 37.9 | 35.3 | 33.7 | 30.4 | 29.1 | 22.9 |
| J01CA | Penicillins with extended spectrum | 51.0 | 51.1 | 49.5 | 49.0 | 48.7 | 43.1 |
| J01CE | Beta-lactamase sensitive penicillins | 89.5 | 91.8 | 88.5 | 82.6 | 77.7 | 52.7 |
| J01CF | Beta-lactamase resistant penicillins | 36.4 | 36.2 | 35.2 | 35.8 | 34.4 | 31.6 |
| J01CR | Combinations of penicillins, including beta-lactamase inhibitors | 6.0 | 6.4 | 6.8 | 6.6 | 7.0 | 5.7 |
| J01EE | Combinations of sulfonamides and trimethoprim, including derivatives | 6.1 | 6.2 | 6.7 | 7.1 | 7.9 | 7.5 |
| J01FA | Macrolides | 8.7 | 8.6 | 8.7 | 7.8 | 8.0 | 6.8 |
| J01FF | Lincosamides | 14.4 | 14.6 | 14.1 | 13.6 | 12.9 | 10.9 |
| J01MA | Fluoroquinolones | 22.4 | 21.9 | 20.7 | 20.1 | 18.6 | 16.4 |
| J01XE | Nitrofuran derivatives | 24.7 | 25.1 | 25.3 | 25.6 | 25.8 | 25.2 |
| J02 | Antimycotics for systemic use | 15.5 | 14.9 | 16.2 | 17.5 | 17.5 | 17.2 |
| J04 | Antimycobacterials | 1.0 | 0.9 | 0.8 | 0.8 | 0.7 | 0.6 |
| J05 | Antivirals for systemic use | 33.5 | 34.9 | 35.1 | 37.5 | 38.8 | 38.7 |
| J05AB | Nucleosides and nucleotides excl. reverse transcriptase inhibitors | 25.8 | 27.5 | 28.3 | 29.5 | 30.9 | 31.2 |
| J05AE | Protease inhibitors | 1.4 | 1.1 | 0.7 | 0.6 | 0.5 | 0.4 |
| J05AF | Nucleoside and nucleotide reverse transcriptase inhibitors | 0.9 | 0.9 | 1.0 | 1.1 | 1.2 | 1.2 |
| J05AG | Non-nucleoside reverse transcriptase inhibitors | 0.4 | 0.4 | 0.4 | 0.4 | 0.3 | 0.3 |
| J05AJ | Integrase inhibitors | 0.3 | 0.4 | 0.5 | 0.6 | 0.8 | 0.9 |
| J05AR | Antivirals for treatment of HIV infections, combinations | 2.5 | 2.6 | 2.7 | 2.8 | 3.2 | 3.4 |
| P01 | Antiprotozoals | 18.4 | 18.8 | 18.0 | 18.0 | 18.2 | 14.3 |
| P01AB | Nitroimidazole derivatives | 9.7 | 9.5 | 8.7 | 8.3 | 8.7 | 7.9 |
| P01BA | Aminoquinolines | 3.1 | 3.2 | 3.3 | 3.4 | 3.5 | 4.0 |
| P01BB | Biguanides | 3.4 | 4.0 | 4.0 | 4.4 | 4.3 | 1.1 |
| P01BC | Methanoquinolines | 2.0 | 1.9 | 1.8 | 1.7 | 1.6 | 0.9 |
| P02 | Anthelmintics | 3.7 | 3.1 | 3.0 | 7.2 | 12.6 | 12.5 |

Table S2 Total number of antimicrobial prescriptions filled per 1000 inhabitants by ATC therapeutic subgroup, age group and year, Sweden, 2015-2020

|  | | | Prescriptions filled per 1000 inhabitants, N | | | | | |
| --- | --- | --- | --- | --- | --- | --- | --- | --- |
|  | | Age group, years | 2015 | 2016 | 2017 | 2018 | 2019 | 2020 |
| J01 excluding J01XX | Antibacterials for systemic use | 0-19 | 262.9 | 271.9 | 252.6 | 239.5 | 224.0 | 148.9 |
|  |  | 20-39 | 244.1 | 244.7 | 232.6 | 221.8 | 215.9 | 183.7 |
|  |  | 40-59 | 276.3 | 270.6 | 261.3 | 249.9 | 241.2 | 207.9 |
|  |  | 60-79 | 426.6 | 419.3 | 411.4 | 397.7 | 389.0 | 334.2 |
|  |  | 80+ | 610.6 | 607.6 | 595.3 | 585.3 | 580.3 | 525.3 |
| J02 | Antimycotics for systemic use | 0-19 | 2.8 | 2.9 | 3.3 | 3.7 | 3.8 | 3.8 |
|  |  | 20-39 | 22.1 | 21.2 | 24.8 | 28.1 | 28.7 | 28.9 |
|  |  | 40-59 | 18.1 | 17.6 | 19.0 | 20.6 | 20.6 | 20.0 |
|  |  | 60-79 | 17.0 | 16.3 | 16.2 | 16.1 | 15.6 | 15.1 |
|  |  | 80+ | 16.7 | 16.2 | 15.7 | 14.9 | 14.0 | 13.1 |
| J04 | Antimycobacterials | 0-19 | 0.7 | 0.5 | 0.4 | 0.4 | 0.3 | 0.2 |
|  |  | 20-39 | 0.9 | 0.8 | 0.6 | 0.5 | 0.5 | 0.4 |
|  |  | 40-59 | 0.8 | 0.7 | 0.6 | 0.6 | 0.5 | 0.6 |
|  |  | 60-79 | 1.5 | 1.5 | 1.4 | 1.4 | 1.4 | 1.2 |
|  |  | 80+ | 1.8 | 1.8 | 1.4 | 1.5 | 1.3 | 1.0 |
| J05 | Antivirals for systemic use | 0-19 | 4.9 | 5.5 | 5.5 | 5.4 | 5.5 | 4.9 |
|  |  | 20-39 | 38.0 | 40.1 | 40.7 | 43.7 | 45.5 | 45.3 |
|  |  | 40-59 | 49.9 | 51.3 | 50.6 | 53.6 | 55.2 | 54.9 |
|  |  | 60-79 | 41.0 | 42.7 | 43.4 | 47.0 | 48.9 | 49.6 |
|  |  | 80+ | 22.5 | 24.4 | 26.4 | 30.2 | 31.5 | 32.6 |
| P01 | Antiprotozoals | 0-19 | 5.7 | 5.8 | 5.9 | 6.1 | 6.1 | 3.0 |
|  |  | 20-39 | 19.5 | 19.5 | 18.1 | 18.0 | 18.4 | 13.3 |
|  |  | 40-59 | 20.8 | 21.6 | 20.6 | 21.0 | 21.5 | 16.7 |
|  |  | 60-79 | 25.4 | 26.0 | 25.4 | 25.2 | 25.6 | 22.0 |
|  |  | 80+ | 27.0 | 28.1 | 26.6 | 26.2 | 25.8 | 25.5 |
| P02 | Anthelmintics | 0-19 | 10.9 | 9.8 | 9.8 | 22.7 | 39.4 | 38.5 |
|  |  | 20-39 | 2.3 | 1.6 | 1.5 | 3.8 | 7.0 | 7.5 |
|  |  | 40-59 | 2.2 | 1.5 | 1.4 | 3.2 | 5.7 | 5.9 |
|  |  | 60-79 | 0.5 | 0.4 | 0.4 | 0.8 | 1.3 | 1.1 |
|  |  | 80+ | 0.1 | 0.1 | 0.0 | 0.0 | 0.1 | 0.1 |
|  |  |  |  |  |  |  |  |  |

Table S3 Total number of antimicrobial prescriptions filled per 1000 inhabitants, by ATC therapeutic subgroup, sex and year, Sweden, 2015-2020

|  | | | Prescriptions filled per 1000 inhabitants, N | | | | | |
| --- | --- | --- | --- | --- | --- | --- | --- | --- |
|  | | Sex | 2015 | 2016 | 2017 | 2018 | 2019 | 2020 |
| J01 excluding J01XX | Antibacterials for systemic use | Men | 250.5 | 250.2 | 239.8 | 229.6 | 222.0 | 179.7 |
|  |  | Women | 377.0 | 375.5 | 361.7 | 348.1 | 337.4 | 284.0 |
| J02 | Antimycotics for systemic use | Men | 4.7 | 4.6 | 4.6 | 4.6 | 4.5 | 4.2 |
|  |  | Women | 26.2 | 25.2 | 28.0 | 30.5 | 30.7 | 30.5 |
| J04 | Antimycobacterials | Men | 1.0 | 1.0 | 0.9 | 0.8 | 0.8 | 0.6 |
|  |  | Women | 0.9 | 0.8 | 0.7 | 0.7 | 0.6 | 0.5 |
| J05 | Antivirals for systemic use | Men | 26.3 | 27.0 | 26.5 | 28.6 | 29.7 | 29.2 |
|  |  | Women | 40.8 | 42.9 | 43.6 | 46.4 | 48.0 | 48.3 |
| P01 | Antiprotozoals | Men | 12.5 | 13.0 | 12.5 | 12.5 | 12.8 | 9.2 |
|  |  | Women | 24.3 | 24.7 | 23.5 | 23.5 | 23.8 | 19.4 |
| P02 | Anthelmintics | Men | 2.7 | 2.3 | 2.2 | 5.3 | 9.4 | 9.4 |
|  |  | Women | 4.7 | 3.9 | 3.8 | 9.0 | 15.7 | 15.6 |

Table S4 Total number of antimicrobial prescriptions filled per 1000 inhabitants, by ATC therapeutic subgroup, geography and year, Sweden, 2015-2020

|  | | | Prescriptions filled per 1000 inhabitants, N | | | | | |
| --- | --- | --- | --- | --- | --- | --- | --- | --- |
|  | | Geography | 2015 | 2016 | 2017 | 2018 | 2019 | 2020 |
| J01 excluding J01XX | Antibacterials for systemic use | Metropolitan | 436.1 | 432.3 | 414.4 | 394.9 | 376.6 | 309.1 |
|  |  | Non-metropolitan | 231.9 | 232.4 | 223.7 | 216.2 | 212.5 | 177.8 |
| J02 | Antimycotics for systemic use | Metropolitan | 21.9 | 21.3 | 23.0 | 24.3 | 24.3 | 23.8 |
|  |  | Non-metropolitan | 11.1 | 10.7 | 11.7 | 12.9 | 12.8 | 12.7 |
| J04 | Antimycobacterials | Metropolitan | 0.9 | 0.9 | 0.9 | 0.8 | 0.7 | 0.6 |
|  |  | Non-metropolitan | 1.0 | 0.9 | 0.7 | 0.7 | 0.7 | 0.6 |
| J05 | Antivirals for systemic use | Metropolitan | 51.7 | 53.5 | 52.8 | 56.1 | 58.1 | 58.3 |
|  |  | Non-metropolitan | 21.5 | 22.6 | 23.1 | 24.9 | 25.6 | 25.2 |
| P01 | Antiprotozoals | Metropolitan | 26.2 | 26.8 | 25.4 | 25.3 | 25.8 | 19.5 |
|  |  | Non-metropolitan | 13.2 | 13.5 | 13.0 | 13.0 | 13.1 | 10.7 |
| P02 | Anthelmintics | Metropolitan | 5.1 | 4.2 | 4.2 | 9.8 | 17.3 | 17.4 |
|  |  | Non-metropolitan | 2.7 | 2.3 | 2.3 | 5.4 | 9.3 | 9.1 |
|  |  |  |  |  |  |  |  |  |

Table S5 Observed versus predicted weekly number of prescriptions filled per 1000 inhabitants for selected ATC therapeutic and chemical subgroups, Sweden, 2020

|  | | | Prescriptions filled per 1000 inhabitants, N | | | | | | |
| --- | --- | --- | --- | --- | --- | --- | --- | --- | --- |
|  |  | Week | Observed | Predicted | Standard  error | Lower  95% CL | Upper  95% CL | Ratio  (observed/predicted) | p-value* |
| J01 excluding J01XX | Antibacterials for systemic use | 2020-01-01 | 4.5 | 4.6 | 0.18 | 4.3 | 5.0 | 0.97 | 0.4334 |
|  |  | 2020-01-08 | 5.7 | 5.6 | 0.18 | 5.2 | 5.9 | 1.02 | 0.6285 |
|  |  | 2020-01-15 | 5.3 | 5.5 | 0.18 | 5.1 | 5.8 | 0.97 | 0.3919 |
|  |  | 2020-01-22 | 5.5 | 5.7 | 0.18 | 5.3 | 6.0 | 0.96 | 0.2297 |
|  |  | 2020-01-29 | 5.6 | 5.8 | 0.18 | 5.5 | 6.2 | 0.95 | 0.1392 |
|  |  | 2020-02-05 | 5.6 | 5.9 | 0.18 | 5.6 | 6.3 | 0.94 | 0.0394 |
|  |  | 2020-02-12 | 5.6 | 5.9 | 0.18 | 5.6 | 6.3 | 0.95 | 0.0826 |
|  |  | 2020-02-19 | 5.6 | 5.9 | 0.18 | 5.5 | 6.2 | 0.95 | 0.1411 |
|  |  | 2020-02-26 | 5.6 | 5.8 | 0.18 | 5.4 | 6.1 | 0.97 | 0.3574 |
|  |  | 2020-03-04 | 5.6 | 5.7 | 0.18 | 5.4 | 6.1 | 0.97 | 0.3889 |
|  |  | 2020-03-11 | 5.5 | 5.7 | 0.18 | 5.3 | 6.0 | 0.97 | 0.3160 |
|  |  | 2020-03-18 | 4.9 | 5.6 | 0.19 | 5.2 | 6.0 | 0.87 | 2.0E-04 |
|  |  | 2020-03-25 | 4.5 | 5.6 | 0.20 | 5.2 | 6.0 | 0.81 | 2.1E-07 |
|  |  | 2020-04-01 | 4.3 | 5.5 | 0.19 | 5.1 | 5.9 | 0.77 | 8.4E-10 |
|  |  | 2020-04-08 | 3.3 | 4.4 | 0.19 | 4.0 | 4.7 | 0.75 | 4.6E-08 |
|  |  | 2020-04-15 | 4.1 | 5.3 | 0.18 | 4.9 | 5.7 | 0.76 | 6.8E-11 |
|  |  | 2020-04-22 | 3.9 | 5.2 | 0.18 | 4.9 | 5.6 | 0.75 | 5.6E-12 |
|  |  | 2020-04-29 | 3.5 | 4.7 | 0.18 | 4.3 | 5.0 | 0.76 | 4.0E-09 |
|  |  | 2020-05-06 | 3.8 | 5.1 | 0.18 | 4.7 | 5.4 | 0.75 | 4.3E-11 |
|  |  | 2020-05-13 | 3.9 | 5.0 | 0.18 | 4.7 | 5.4 | 0.77 | 9.2E-10 |
|  |  | 2020-05-20 | 3.5 | 4.5 | 0.18 | 4.2 | 4.9 | 0.77 | 4.8E-08 |
|  |  | 2020-05-27 | 3.9 | 5.1 | 0.18 | 4.7 | 5.5 | 0.76 | 2.3E-10 |
|  |  | 2020-06-03 | 4.0 | 4.7 | 0.18 | 4.4 | 5.1 | 0.84 | 4.2E-05 |
|  |  | 2020-06-10 | 4.0 | 5.2 | 0.18 | 4.9 | 5.6 | 0.77 | 4.6E-10 |
|  |  | 2020-06-17 | 3.8 | 4.7 | 0.18 | 4.3 | 5.0 | 0.81 | 2.3E-06 |
|  |  | 2020-06-24 | 4.3 | 5.4 | 0.18 | 5.0 | 5.7 | 0.81 | 7.0E-08 |
|  |  | 2020-07-01 | 4.3 | 5.0 | 0.18 | 4.7 | 5.4 | 0.86 | 1.5E-04 |
|  |  | 2020-07-08 | 4.2 | 4.8 | 0.18 | 4.4 | 5.2 | 0.88 | 0.0016 |
|  |  | 2020-07-15 | 4.1 | 4.6 | 0.18 | 4.3 | 5.0 | 0.89 | 0.0071 |
|  |  | 2020-07-22 | 4.1 | 4.6 | 0.18 | 4.2 | 4.9 | 0.89 | 0.0077 |
|  |  | 2020-07-29 | 4.2 | 4.7 | 0.18 | 4.3 | 5.0 | 0.89 | 0.0071 |
|  |  | 2020-08-05 | 4.3 | 4.8 | 0.18 | 4.4 | 5.1 | 0.89 | 0.0043 |
|  |  | 2020-08-12 | 4.3 | 4.9 | 0.18 | 4.6 | 5.3 | 0.87 | 7.6E-04 |
|  |  | 2020-08-19 | 4.6 | 5.1 | 0.18 | 4.7 | 5.4 | 0.90 | 0.0064 |
|  |  | 2020-08-26 | 4.5 | 5.2 | 0.18 | 4.9 | 5.6 | 0.86 | 7.6E-05 |
|  |  | 2020-09-02 | 4.6 | 5.3 | 0.18 | 5.0 | 5.7 | 0.85 | 2.0E-05 |
|  |  | 2020-09-09 | 4.5 | 5.4 | 0.18 | 5.0 | 5.7 | 0.83 | 1.3E-06 |
|  |  | 2020-09-16 | 4.5 | 5.4 | 0.18 | 5.0 | 5.8 | 0.84 | 2.1E-06 |
|  |  | 2020-09-23 | 4.6 | 5.4 | 0.18 | 5.0 | 5.7 | 0.85 | 9.7E-06 |
|  |  | 2020-09-30 | 4.6 | 5.4 | 0.18 | 5.0 | 5.7 | 0.85 | 1.3E-05 |
|  |  | 2020-10-07 | 4.5 | 5.4 | 0.18 | 5.0 | 5.7 | 0.83 | 1.9E-06 |
|  |  | 2020-10-14 | 4.4 | 5.4 | 0.18 | 5.0 | 5.7 | 0.83 | 8.6E-07 |
|  |  | 2020-10-21 | 4.4 | 5.3 | 0.18 | 4.9 | 5.7 | 0.83 | 1.6E-06 |
|  |  | 2020-10-28 | 4.3 | 5.0 | 0.18 | 4.7 | 5.4 | 0.85 | 3.0E-05 |
|  |  | 2020-11-04 | 4.2 | 5.3 | 0.18 | 4.9 | 5.6 | 0.81 | 5.1E-08 |
|  |  | 2020-11-11 | 4.2 | 5.1 | 0.18 | 4.8 | 5.5 | 0.82 | 1.0E-06 |
|  |  | 2020-11-18 | 4.2 | 5.2 | 0.18 | 4.8 | 5.5 | 0.82 | 4.2E-07 |
|  |  | 2020-11-25 | 4.2 | 5.2 | 0.18 | 4.8 | 5.6 | 0.80 | 4.5E-08 |
|  |  | 2020-12-02 | 4.2 | 5.3 | 0.18 | 5.0 | 5.7 | 0.79 | 5.3E-09 |
|  |  | 2020-12-09 | 4.2 | 5.5 | 0.18 | 5.2 | 5.9 | 0.76 | 8.2E-12 |
|  |  | 2020-12-16 | 4.5 | 5.8 | 0.18 | 5.5 | 6.2 | 0.77 | 8.1E-12 |
|  |  | 2020-12-23 | 3.2 | 4.2 | 0.18 | 3.8 | 4.5 | 0.77 | 3.4E-07 |
| J01AA | Tetracyclines | 2020-01-01 | 0.52 | 0.52 | 0.045 | 0.43 | 0.61 | 1.00 | 0.9807 |
|  |  | 2020-01-08 | 0.68 | 0.68 | 0.045 | 0.59 | 0.77 | 1.00 | 0.9950 |
|  |  | 2020-01-15 | 0.62 | 0.62 | 0.045 | 0.53 | 0.71 | 1.00 | 0.9851 |
|  |  | 2020-01-22 | 0.60 | 0.63 | 0.045 | 0.55 | 0.72 | 0.95 | 0.5122 |
|  |  | 2020-01-29 | 0.60 | 0.64 | 0.045 | 0.55 | 0.73 | 0.94 | 0.3533 |
|  |  | 2020-02-05 | 0.60 | 0.65 | 0.045 | 0.56 | 0.74 | 0.92 | 0.2652 |
|  |  | 2020-02-12 | 0.60 | 0.66 | 0.045 | 0.57 | 0.75 | 0.91 | 0.1709 |
|  |  | 2020-02-19 | 0.61 | 0.67 | 0.045 | 0.58 | 0.76 | 0.91 | 0.1868 |
|  |  | 2020-02-26 | 0.64 | 0.65 | 0.045 | 0.57 | 0.74 | 0.97 | 0.7021 |
|  |  | 2020-03-04 | 0.62 | 0.64 | 0.045 | 0.56 | 0.73 | 0.96 | 0.5985 |
|  |  | 2020-03-11 | 0.69 | 0.62 | 0.046 | 0.53 | 0.71 | 1.12 | 0.1212 |
|  |  | 2020-03-18 | 0.57 | 0.61 | 0.046 | 0.52 | 0.70 | 0.95 | 0.4882 |
|  |  | 2020-03-25 | 0.55 | 0.60 | 0.049 | 0.51 | 0.70 | 0.91 | 0.2622 |
|  |  | 2020-04-01 | 0.48 | 0.59 | 0.048 | 0.50 | 0.69 | 0.81 | 0.0226 |
|  |  | 2020-04-08 | 0.33 | 0.41 | 0.048 | 0.31 | 0.50 | 0.81 | 0.1145 |
|  |  | 2020-04-15 | 0.44 | 0.54 | 0.045 | 0.45 | 0.63 | 0.82 | 0.0353 |
|  |  | 2020-04-22 | 0.41 | 0.52 | 0.045 | 0.43 | 0.61 | 0.79 | 0.0167 |
|  |  | 2020-04-29 | 0.34 | 0.41 | 0.045 | 0.32 | 0.50 | 0.83 | 0.1219 |
|  |  | 2020-05-06 | 0.37 | 0.45 | 0.045 | 0.36 | 0.54 | 0.82 | 0.0728 |
|  |  | 2020-05-13 | 0.36 | 0.43 | 0.045 | 0.34 | 0.51 | 0.85 | 0.1653 |
|  |  | 2020-05-20 | 0.30 | 0.34 | 0.045 | 0.25 | 0.43 | 0.88 | 0.3797 |
|  |  | 2020-05-27 | 0.34 | 0.40 | 0.045 | 0.31 | 0.49 | 0.84 | 0.1507 |
|  |  | 2020-06-03 | 0.32 | 0.34 | 0.045 | 0.25 | 0.43 | 0.93 | 0.5955 |
|  |  | 2020-06-10 | 0.30 | 0.39 | 0.045 | 0.31 | 0.48 | 0.77 | 0.0465 |
|  |  | 2020-06-17 | 0.27 | 0.32 | 0.045 | 0.23 | 0.41 | 0.85 | 0.2905 |
|  |  | 2020-06-24 | 0.30 | 0.41 | 0.045 | 0.32 | 0.49 | 0.74 | 0.0213 |
|  |  | 2020-07-01 | 0.32 | 0.36 | 0.045 | 0.27 | 0.45 | 0.89 | 0.3755 |
|  |  | 2020-07-08 | 0.32 | 0.33 | 0.045 | 0.24 | 0.42 | 0.98 | 0.9096 |
|  |  | 2020-07-15 | 0.32 | 0.31 | 0.045 | 0.22 | 0.40 | 1.01 | 0.9243 |
|  |  | 2020-07-22 | 0.32 | 0.30 | 0.045 | 0.21 | 0.39 | 1.07 | 0.6557 |
|  |  | 2020-07-29 | 0.33 | 0.32 | 0.045 | 0.23 | 0.41 | 1.03 | 0.8200 |
|  |  | 2020-08-05 | 0.34 | 0.36 | 0.045 | 0.27 | 0.45 | 0.93 | 0.5773 |
|  |  | 2020-08-12 | 0.33 | 0.42 | 0.045 | 0.33 | 0.51 | 0.78 | 0.0460 |
|  |  | 2020-08-19 | 0.40 | 0.46 | 0.045 | 0.38 | 0.55 | 0.85 | 0.1252 |
|  |  | 2020-08-26 | 0.43 | 0.50 | 0.045 | 0.41 | 0.59 | 0.86 | 0.1118 |
|  |  | 2020-09-02 | 0.44 | 0.53 | 0.045 | 0.44 | 0.62 | 0.82 | 0.0344 |
|  |  | 2020-09-09 | 0.44 | 0.54 | 0.045 | 0.46 | 0.63 | 0.80 | 0.0164 |
|  |  | 2020-09-16 | 0.43 | 0.56 | 0.045 | 0.47 | 0.65 | 0.76 | 0.0034 |
|  |  | 2020-09-23 | 0.46 | 0.57 | 0.045 | 0.48 | 0.66 | 0.80 | 0.0123 |
|  |  | 2020-09-30 | 0.44 | 0.58 | 0.045 | 0.49 | 0.67 | 0.76 | 0.0025 |
|  |  | 2020-10-07 | 0.44 | 0.57 | 0.045 | 0.49 | 0.66 | 0.76 | 0.0029 |
|  |  | 2020-10-14 | 0.43 | 0.57 | 0.045 | 0.48 | 0.66 | 0.76 | 0.0024 |
|  |  | 2020-10-21 | 0.46 | 0.58 | 0.045 | 0.49 | 0.67 | 0.79 | 0.0068 |
|  |  | 2020-10-28 | 0.43 | 0.54 | 0.045 | 0.45 | 0.63 | 0.80 | 0.0162 |
|  |  | 2020-11-04 | 0.42 | 0.57 | 0.045 | 0.49 | 0.66 | 0.73 | 5.8E-04 |
|  |  | 2020-11-11 | 0.44 | 0.56 | 0.045 | 0.47 | 0.65 | 0.80 | 0.0125 |
|  |  | 2020-11-18 | 0.43 | 0.56 | 0.045 | 0.47 | 0.65 | 0.78 | 0.0067 |
|  |  | 2020-11-25 | 0.45 | 0.55 | 0.045 | 0.46 | 0.64 | 0.82 | 0.0293 |
|  |  | 2020-12-02 | 0.45 | 0.57 | 0.045 | 0.48 | 0.66 | 0.79 | 0.0102 |
|  |  | 2020-12-09 | 0.44 | 0.59 | 0.045 | 0.51 | 0.68 | 0.74 | 6.3E-04 |
|  |  | 2020-12-16 | 0.46 | 0.62 | 0.046 | 0.53 | 0.71 | 0.75 | 7.4E-04 |
|  |  | 2020-12-23 | 0.30 | 0.40 | 0.045 | 0.31 | 0.49 | 0.76 | 0.0337 |
| J01CA | Penicillins with extended spectrum | 2020-01-01 | 0.80 | 0.83 | 0.033 | 0.77 | 0.90 | 0.96 | 0.3053 |
|  |  | 2020-01-08 | 1.0 | 0.99 | 0.033 | 0.92 | 1.1 | 1.05 | 0.1620 |
|  |  | 2020-01-15 | 0.97 | 0.97 | 0.033 | 0.90 | 1.0 | 1.01 | 0.7766 |
|  |  | 2020-01-22 | 1.0 | 0.98 | 0.033 | 0.92 | 1.0 | 1.02 | 0.5399 |
|  |  | 2020-01-29 | 0.97 | 0.99 | 0.033 | 0.93 | 1.1 | 0.98 | 0.5738 |
|  |  | 2020-02-05 | 0.97 | 1.0 | 0.033 | 0.94 | 1.1 | 0.97 | 0.3266 |
|  |  | 2020-02-12 | 1.00 | 1.0 | 0.033 | 0.95 | 1.1 | 0.99 | 0.6815 |
|  |  | 2020-02-19 | 0.98 | 0.99 | 0.033 | 0.92 | 1.1 | 0.99 | 0.7174 |
|  |  | 2020-02-26 | 0.98 | 0.98 | 0.033 | 0.92 | 1.0 | 0.99 | 0.8634 |
|  |  | 2020-03-04 | 0.98 | 0.97 | 0.033 | 0.90 | 1.0 | 1.01 | 0.7662 |
|  |  | 2020-03-11 | 0.97 | 0.96 | 0.034 | 0.90 | 1.0 | 1.01 | 0.8487 |
|  |  | 2020-03-18 | 0.88 | 0.96 | 0.034 | 0.89 | 1.0 | 0.92 | 0.0186 |
|  |  | 2020-03-25 | 0.81 | 0.97 | 0.036 | 0.90 | 1.0 | 0.84 | 1.8E-05 |
|  |  | 2020-04-01 | 0.79 | 0.95 | 0.035 | 0.88 | 1.0 | 0.83 | 1.2E-05 |
|  |  | 2020-04-08 | 0.60 | 0.77 | 0.035 | 0.70 | 0.84 | 0.79 | 5.4E-06 |
|  |  | 2020-04-15 | 0.79 | 0.92 | 0.033 | 0.86 | 0.99 | 0.85 | 5.6E-05 |
|  |  | 2020-04-22 | 0.72 | 0.92 | 0.033 | 0.85 | 0.98 | 0.78 | 3.5E-09 |
|  |  | 2020-04-29 | 0.67 | 0.83 | 0.033 | 0.76 | 0.89 | 0.81 | 4.0E-06 |
|  |  | 2020-05-06 | 0.73 | 0.90 | 0.033 | 0.84 | 0.97 | 0.81 | 4.3E-07 |
|  |  | 2020-05-13 | 0.73 | 0.89 | 0.033 | 0.82 | 0.95 | 0.83 | 6.5E-06 |
|  |  | 2020-05-20 | 0.65 | 0.81 | 0.033 | 0.74 | 0.87 | 0.81 | 8.8E-06 |
|  |  | 2020-05-27 | 0.71 | 0.89 | 0.033 | 0.82 | 0.95 | 0.80 | 1.6E-07 |
|  |  | 2020-06-03 | 0.74 | 0.83 | 0.033 | 0.77 | 0.90 | 0.89 | 0.0045 |
|  |  | 2020-06-10 | 0.73 | 0.92 | 0.033 | 0.86 | 0.99 | 0.79 | 2.8E-08 |
|  |  | 2020-06-17 | 0.70 | 0.82 | 0.033 | 0.76 | 0.89 | 0.86 | 4.4E-04 |
|  |  | 2020-06-24 | 0.75 | 0.92 | 0.033 | 0.85 | 0.98 | 0.81 | 5.6E-07 |
|  |  | 2020-07-01 | 0.80 | 0.88 | 0.033 | 0.81 | 0.94 | 0.91 | 0.0125 |
|  |  | 2020-07-08 | 0.78 | 0.85 | 0.033 | 0.78 | 0.91 | 0.92 | 0.0361 |
|  |  | 2020-07-15 | 0.77 | 0.82 | 0.033 | 0.76 | 0.88 | 0.93 | 0.0958 |
|  |  | 2020-07-22 | 0.76 | 0.80 | 0.033 | 0.74 | 0.87 | 0.95 | 0.2264 |
|  |  | 2020-07-29 | 0.76 | 0.84 | 0.033 | 0.77 | 0.90 | 0.91 | 0.0267 |
|  |  | 2020-08-05 | 0.77 | 0.86 | 0.033 | 0.80 | 0.92 | 0.89 | 0.0052 |
|  |  | 2020-08-12 | 0.78 | 0.89 | 0.033 | 0.83 | 0.96 | 0.87 | 5.9E-04 |
|  |  | 2020-08-19 | 0.84 | 0.93 | 0.033 | 0.86 | 0.99 | 0.91 | 0.0100 |
|  |  | 2020-08-26 | 0.87 | 0.95 | 0.033 | 0.88 | 1.0 | 0.91 | 0.0135 |
|  |  | 2020-09-02 | 0.87 | 0.97 | 0.033 | 0.91 | 1.0 | 0.90 | 0.0041 |
|  |  | 2020-09-09 | 0.87 | 0.98 | 0.033 | 0.92 | 1.0 | 0.88 | 5.8E-04 |
|  |  | 2020-09-16 | 0.86 | 0.98 | 0.033 | 0.92 | 1.0 | 0.87 | 2.0E-04 |
|  |  | 2020-09-23 | 0.87 | 0.99 | 0.033 | 0.92 | 1.1 | 0.88 | 4.2E-04 |
|  |  | 2020-09-30 | 0.88 | 0.97 | 0.033 | 0.91 | 1.0 | 0.91 | 0.0085 |
|  |  | 2020-10-07 | 0.87 | 0.97 | 0.033 | 0.91 | 1.0 | 0.90 | 0.0033 |
|  |  | 2020-10-14 | 0.87 | 0.97 | 0.033 | 0.91 | 1.0 | 0.90 | 0.0028 |
|  |  | 2020-10-21 | 0.86 | 0.96 | 0.033 | 0.89 | 1.0 | 0.90 | 0.0033 |
|  |  | 2020-10-28 | 0.84 | 0.92 | 0.033 | 0.85 | 0.98 | 0.91 | 0.0172 |
|  |  | 2020-11-04 | 0.84 | 0.95 | 0.033 | 0.89 | 1.0 | 0.88 | 4.1E-04 |
|  |  | 2020-11-11 | 0.84 | 0.93 | 0.033 | 0.86 | 0.99 | 0.90 | 0.0053 |
|  |  | 2020-11-18 | 0.81 | 0.93 | 0.033 | 0.86 | 0.99 | 0.88 | 6.0E-04 |
|  |  | 2020-11-25 | 0.84 | 0.93 | 0.033 | 0.86 | 0.99 | 0.90 | 0.0064 |
|  |  | 2020-12-02 | 0.83 | 0.94 | 0.033 | 0.88 | 1.0 | 0.88 | 7.5E-04 |
|  |  | 2020-12-09 | 0.83 | 0.97 | 0.033 | 0.91 | 1.0 | 0.86 | 3.5E-05 |
|  |  | 2020-12-16 | 0.90 | 1.0 | 0.033 | 0.97 | 1.1 | 0.87 | 8.1E-05 |
|  |  | 2020-12-23 | 0.65 | 0.74 | 0.033 | 0.68 | 0.81 | 0.88 | 0.0065 |
| J01CE | Beta-lactamase sensitive penicillins | 2020-01-01 | 1.3 | 1.4 | 0.082 | 1.2 | 1.6 | 0.95 | 0.4139 |
|  |  | 2020-01-08 | 1.5 | 1.5 | 0.082 | 1.4 | 1.7 | 0.95 | 0.3366 |
|  |  | 2020-01-15 | 1.4 | 1.5 | 0.082 | 1.4 | 1.7 | 0.88 | 0.0271 |
|  |  | 2020-01-22 | 1.5 | 1.7 | 0.082 | 1.5 | 1.9 | 0.86 | 0.0037 |
|  |  | 2020-01-29 | 1.6 | 1.8 | 0.082 | 1.7 | 2.0 | 0.87 | 0.0035 |
|  |  | 2020-02-05 | 1.7 | 1.9 | 0.082 | 1.8 | 2.1 | 0.86 | 0.0013 |
|  |  | 2020-02-12 | 1.7 | 1.9 | 0.082 | 1.8 | 2.1 | 0.87 | 0.0031 |
|  |  | 2020-02-19 | 1.7 | 1.9 | 0.082 | 1.7 | 2.0 | 0.89 | 0.0114 |
|  |  | 2020-02-26 | 1.6 | 1.8 | 0.082 | 1.6 | 2.0 | 0.90 | 0.0220 |
|  |  | 2020-03-04 | 1.6 | 1.8 | 0.082 | 1.6 | 1.9 | 0.89 | 0.0207 |
|  |  | 2020-03-11 | 1.4 | 1.7 | 0.084 | 1.6 | 1.9 | 0.80 | 3.7E-05 |
|  |  | 2020-03-18 | 1.2 | 1.7 | 0.085 | 1.5 | 1.9 | 0.70 | 8.7E-09 |
|  |  | 2020-03-25 | 1.0 | 1.7 | 0.089 | 1.5 | 1.8 | 0.61 | 7.0E-12 |
|  |  | 2020-04-01 | 0.89 | 1.6 | 0.088 | 1.5 | 1.8 | 0.55 | 7.3E-15 |
|  |  | 2020-04-08 | 0.69 | 1.3 | 0.088 | 1.1 | 1.5 | 0.52 | 1.2E-11 |
|  |  | 2020-04-15 | 0.79 | 1.5 | 0.082 | 1.3 | 1.7 | 0.53 | 3.9E-15 |
|  |  | 2020-04-22 | 0.76 | 1.4 | 0.082 | 1.3 | 1.6 | 0.53 | 1.8E-14 |
|  |  | 2020-04-29 | 0.70 | 1.3 | 0.082 | 1.1 | 1.5 | 0.54 | 1.1E-11 |
|  |  | 2020-05-06 | 0.73 | 1.4 | 0.082 | 1.2 | 1.6 | 0.52 | 1.7E-14 |
|  |  | 2020-05-13 | 0.75 | 1.4 | 0.082 | 1.2 | 1.6 | 0.54 | 2.1E-13 |
|  |  | 2020-05-20 | 0.70 | 1.3 | 0.083 | 1.1 | 1.4 | 0.56 | 2.5E-10 |
|  |  | 2020-05-27 | 0.81 | 1.4 | 0.082 | 1.2 | 1.6 | 0.57 | 4.3E-12 |
|  |  | 2020-06-03 | 0.85 | 1.4 | 0.082 | 1.2 | 1.5 | 0.63 | 4.8E-09 |
|  |  | 2020-06-10 | 0.90 | 1.5 | 0.082 | 1.3 | 1.6 | 0.62 | 7.0E-11 |
|  |  | 2020-06-17 | 0.89 | 1.4 | 0.082 | 1.2 | 1.5 | 0.66 | 5.0E-08 |
|  |  | 2020-06-24 | 1.1 | 1.6 | 0.082 | 1.4 | 1.7 | 0.70 | 6.8E-08 |
|  |  | 2020-07-01 | 1.0 | 1.4 | 0.082 | 1.3 | 1.6 | 0.73 | 3.1E-06 |
|  |  | 2020-07-08 | 1.0 | 1.4 | 0.082 | 1.2 | 1.5 | 0.74 | 3.3E-05 |
|  |  | 2020-07-15 | 0.99 | 1.3 | 0.082 | 1.1 | 1.4 | 0.78 | 8.2E-04 |
|  |  | 2020-07-22 | 0.95 | 1.3 | 0.082 | 1.1 | 1.4 | 0.75 | 2.1E-04 |
|  |  | 2020-07-29 | 0.99 | 1.2 | 0.082 | 1.1 | 1.4 | 0.80 | 0.0022 |
|  |  | 2020-08-05 | 1.0 | 1.2 | 0.082 | 1.1 | 1.4 | 0.83 | 0.0126 |
|  |  | 2020-08-12 | 1.0 | 1.2 | 0.082 | 1.1 | 1.4 | 0.86 | 0.0390 |
|  |  | 2020-08-19 | 1.0 | 1.3 | 0.082 | 1.1 | 1.4 | 0.82 | 0.0055 |
|  |  | 2020-08-26 | 0.95 | 1.3 | 0.082 | 1.2 | 1.5 | 0.71 | 7.2E-06 |
|  |  | 2020-09-02 | 0.96 | 1.4 | 0.082 | 1.2 | 1.5 | 0.70 | 9.9E-07 |
|  |  | 2020-09-09 | 0.95 | 1.4 | 0.082 | 1.3 | 1.6 | 0.67 | 5.0E-08 |
|  |  | 2020-09-16 | 0.93 | 1.4 | 0.082 | 1.2 | 1.6 | 0.66 | 3.5E-08 |
|  |  | 2020-09-23 | 0.91 | 1.4 | 0.082 | 1.2 | 1.5 | 0.66 | 4.7E-08 |
|  |  | 2020-09-30 | 0.92 | 1.4 | 0.082 | 1.2 | 1.6 | 0.66 | 1.7E-08 |
|  |  | 2020-10-07 | 0.89 | 1.4 | 0.082 | 1.2 | 1.6 | 0.64 | 4.3E-09 |
|  |  | 2020-10-14 | 0.87 | 1.4 | 0.082 | 1.2 | 1.5 | 0.63 | 1.5E-09 |
|  |  | 2020-10-21 | 0.87 | 1.4 | 0.082 | 1.2 | 1.5 | 0.64 | 6.7E-09 |
|  |  | 2020-10-28 | 0.82 | 1.3 | 0.082 | 1.1 | 1.5 | 0.63 | 2.6E-08 |
|  |  | 2020-11-04 | 0.82 | 1.4 | 0.082 | 1.2 | 1.5 | 0.60 | 1.4E-10 |
|  |  | 2020-11-11 | 0.78 | 1.3 | 0.082 | 1.2 | 1.5 | 0.59 | 2.1E-10 |
|  |  | 2020-11-18 | 0.78 | 1.3 | 0.082 | 1.2 | 1.5 | 0.58 | 8.9E-11 |
|  |  | 2020-11-25 | 0.76 | 1.4 | 0.082 | 1.2 | 1.6 | 0.55 | 7.4E-13 |
|  |  | 2020-12-02 | 0.75 | 1.5 | 0.082 | 1.3 | 1.6 | 0.51 | 1.5E-15 |
|  |  | 2020-12-09 | 0.75 | 1.5 | 0.082 | 1.4 | 1.7 | 0.49 | 1.7E-18 |
|  |  | 2020-12-16 | 0.79 | 1.7 | 0.083 | 1.5 | 1.8 | 0.48 | 2.3E-20 |
|  |  | 2020-12-23 | 0.58 | 1.3 | 0.082 | 1.2 | 1.5 | 0.44 | 1.2E-16 |
| J01CF | Beta-lactamase resistant penicillins | 2020-01-01 | 0.47 | 0.49 | 0.029 | 0.43 | 0.55 | 0.96 | 0.5338 |
|  |  | 2020-01-08 | 0.63 | 0.61 | 0.028 | 0.56 | 0.67 | 1.03 | 0.4581 |
|  |  | 2020-01-15 | 0.62 | 0.63 | 0.028 | 0.58 | 0.69 | 0.99 | 0.8140 |
|  |  | 2020-01-22 | 0.64 | 0.63 | 0.028 | 0.57 | 0.69 | 1.01 | 0.7890 |
|  |  | 2020-01-29 | 0.63 | 0.62 | 0.028 | 0.57 | 0.68 | 1.01 | 0.8048 |
|  |  | 2020-02-05 | 0.62 | 0.63 | 0.028 | 0.57 | 0.68 | 1.00 | 0.9572 |
|  |  | 2020-02-12 | 0.62 | 0.62 | 0.028 | 0.56 | 0.67 | 1.00 | 0.9323 |
|  |  | 2020-02-19 | 0.62 | 0.61 | 0.028 | 0.56 | 0.67 | 1.01 | 0.7889 |
|  |  | 2020-02-26 | 0.63 | 0.62 | 0.028 | 0.56 | 0.67 | 1.02 | 0.5943 |
|  |  | 2020-03-04 | 0.65 | 0.63 | 0.028 | 0.57 | 0.68 | 1.04 | 0.4218 |
|  |  | 2020-03-11 | 0.62 | 0.64 | 0.029 | 0.58 | 0.70 | 0.97 | 0.5608 |
|  |  | 2020-03-18 | 0.57 | 0.64 | 0.029 | 0.58 | 0.70 | 0.89 | 0.0196 |
|  |  | 2020-03-25 | 0.55 | 0.65 | 0.031 | 0.59 | 0.71 | 0.85 | 0.0021 |
|  |  | 2020-04-01 | 0.56 | 0.64 | 0.030 | 0.58 | 0.70 | 0.87 | 0.0057 |
|  |  | 2020-04-08 | 0.45 | 0.53 | 0.030 | 0.47 | 0.59 | 0.86 | 0.0136 |
|  |  | 2020-04-15 | 0.57 | 0.66 | 0.029 | 0.60 | 0.72 | 0.86 | 0.0017 |
|  |  | 2020-04-22 | 0.58 | 0.67 | 0.028 | 0.61 | 0.73 | 0.86 | 9.4E-04 |
|  |  | 2020-04-29 | 0.52 | 0.61 | 0.029 | 0.56 | 0.67 | 0.85 | 0.0018 |
|  |  | 2020-05-06 | 0.56 | 0.67 | 0.028 | 0.62 | 0.73 | 0.83 | 1.2E-04 |
|  |  | 2020-05-13 | 0.56 | 0.69 | 0.028 | 0.63 | 0.74 | 0.81 | 9.7E-06 |
|  |  | 2020-05-20 | 0.52 | 0.65 | 0.029 | 0.59 | 0.70 | 0.80 | 1.5E-05 |
|  |  | 2020-05-27 | 0.61 | 0.71 | 0.028 | 0.65 | 0.76 | 0.86 | 4.8E-04 |
|  |  | 2020-06-03 | 0.62 | 0.68 | 0.028 | 0.62 | 0.74 | 0.92 | 0.0485 |
|  |  | 2020-06-10 | 0.63 | 0.74 | 0.028 | 0.68 | 0.79 | 0.86 | 4.9E-04 |
|  |  | 2020-06-17 | 0.61 | 0.66 | 0.028 | 0.60 | 0.71 | 0.93 | 0.0908 |
|  |  | 2020-06-24 | 0.73 | 0.73 | 0.028 | 0.68 | 0.79 | 0.99 | 0.7942 |
|  |  | 2020-07-01 | 0.67 | 0.72 | 0.028 | 0.67 | 0.78 | 0.93 | 0.0703 |
|  |  | 2020-07-08 | 0.65 | 0.70 | 0.028 | 0.64 | 0.75 | 0.93 | 0.0994 |
|  |  | 2020-07-15 | 0.62 | 0.70 | 0.028 | 0.64 | 0.76 | 0.89 | 0.0078 |
|  |  | 2020-07-22 | 0.62 | 0.71 | 0.028 | 0.65 | 0.77 | 0.87 | 0.0018 |
|  |  | 2020-07-29 | 0.63 | 0.72 | 0.028 | 0.67 | 0.78 | 0.87 | 0.0014 |
|  |  | 2020-08-05 | 0.66 | 0.73 | 0.028 | 0.67 | 0.78 | 0.91 | 0.0197 |
|  |  | 2020-08-12 | 0.69 | 0.73 | 0.028 | 0.68 | 0.79 | 0.94 | 0.1151 |
|  |  | 2020-08-19 | 0.72 | 0.74 | 0.028 | 0.68 | 0.79 | 0.98 | 0.5848 |
|  |  | 2020-08-26 | 0.67 | 0.74 | 0.028 | 0.68 | 0.80 | 0.91 | 0.0191 |
|  |  | 2020-09-02 | 0.66 | 0.72 | 0.028 | 0.67 | 0.78 | 0.92 | 0.0312 |
|  |  | 2020-09-09 | 0.64 | 0.71 | 0.028 | 0.66 | 0.77 | 0.90 | 0.0103 |
|  |  | 2020-09-16 | 0.66 | 0.71 | 0.028 | 0.65 | 0.76 | 0.93 | 0.0819 |
|  |  | 2020-09-23 | 0.67 | 0.70 | 0.028 | 0.64 | 0.76 | 0.95 | 0.2597 |
|  |  | 2020-09-30 | 0.64 | 0.69 | 0.028 | 0.63 | 0.74 | 0.93 | 0.1000 |
|  |  | 2020-10-07 | 0.64 | 0.69 | 0.028 | 0.63 | 0.74 | 0.93 | 0.0768 |
|  |  | 2020-10-14 | 0.63 | 0.68 | 0.028 | 0.63 | 0.74 | 0.92 | 0.0548 |
|  |  | 2020-10-21 | 0.61 | 0.66 | 0.028 | 0.60 | 0.71 | 0.93 | 0.1228 |
|  |  | 2020-10-28 | 0.59 | 0.64 | 0.028 | 0.58 | 0.69 | 0.92 | 0.0705 |
|  |  | 2020-11-04 | 0.60 | 0.65 | 0.028 | 0.59 | 0.71 | 0.92 | 0.0693 |
|  |  | 2020-11-11 | 0.57 | 0.64 | 0.028 | 0.58 | 0.69 | 0.89 | 0.0151 |
|  |  | 2020-11-18 | 0.58 | 0.63 | 0.028 | 0.58 | 0.69 | 0.92 | 0.0589 |
|  |  | 2020-11-25 | 0.57 | 0.64 | 0.028 | 0.58 | 0.70 | 0.89 | 0.0166 |
|  |  | 2020-12-02 | 0.58 | 0.64 | 0.028 | 0.58 | 0.69 | 0.91 | 0.0404 |
|  |  | 2020-12-09 | 0.56 | 0.65 | 0.028 | 0.59 | 0.71 | 0.86 | 0.0015 |
|  |  | 2020-12-16 | 0.62 | 0.66 | 0.029 | 0.60 | 0.72 | 0.93 | 0.1307 |
|  |  | 2020-12-23 | 0.45 | 0.46 | 0.029 | 0.41 | 0.52 | 0.96 | 0.5200 |
| J01CR | Combinations of penicillin, including beta-lactamase inhibitors | 2020-01-01 | 0.12 | 0.13 | 0.0076 | 0.11 | 0.14 | 0.92 | 0.1765 |
|  |  | 2020-01-08 | 0.14 | 0.14 | 0.0075 | 0.13 | 0.16 | 1.00 | 0.9664 |
|  |  | 2020-01-15 | 0.13 | 0.14 | 0.0075 | 0.13 | 0.16 | 0.92 | 0.1550 |
|  |  | 2020-01-22 | 0.13 | 0.15 | 0.0075 | 0.13 | 0.16 | 0.92 | 0.1339 |
|  |  | 2020-01-29 | 0.14 | 0.15 | 0.0075 | 0.14 | 0.17 | 0.89 | 0.0321 |
|  |  | 2020-02-05 | 0.14 | 0.16 | 0.0075 | 0.14 | 0.17 | 0.90 | 0.0362 |
|  |  | 2020-02-12 | 0.14 | 0.16 | 0.0075 | 0.14 | 0.17 | 0.88 | 0.0145 |
|  |  | 2020-02-19 | 0.15 | 0.16 | 0.0075 | 0.15 | 0.17 | 0.93 | 0.1455 |
|  |  | 2020-02-26 | 0.15 | 0.16 | 0.0075 | 0.14 | 0.17 | 0.97 | 0.4763 |
|  |  | 2020-03-04 | 0.15 | 0.15 | 0.0075 | 0.14 | 0.17 | 0.98 | 0.7514 |
|  |  | 2020-03-11 | 0.16 | 0.15 | 0.0077 | 0.14 | 0.17 | 1.06 | 0.2741 |
|  |  | 2020-03-18 | 0.13 | 0.15 | 0.0077 | 0.13 | 0.17 | 0.87 | 0.0139 |
|  |  | 2020-03-25 | 0.13 | 0.15 | 0.0081 | 0.14 | 0.17 | 0.82 | 7.9E-04 |
|  |  | 2020-04-01 | 0.12 | 0.15 | 0.0080 | 0.14 | 0.17 | 0.78 | 3.7E-05 |
|  |  | 2020-04-08 | 0.093 | 0.13 | 0.0081 | 0.12 | 0.15 | 0.70 | 1.6E-06 |
|  |  | 2020-04-15 | 0.10 | 0.15 | 0.0076 | 0.13 | 0.16 | 0.70 | 1.5E-08 |
|  |  | 2020-04-22 | 0.11 | 0.14 | 0.0075 | 0.13 | 0.16 | 0.75 | 3.1E-06 |
|  |  | 2020-04-29 | 0.089 | 0.13 | 0.0076 | 0.12 | 0.15 | 0.67 | 2.3E-08 |
|  |  | 2020-05-06 | 0.100 | 0.14 | 0.0075 | 0.13 | 0.16 | 0.70 | 5.8E-08 |
|  |  | 2020-05-13 | 0.097 | 0.14 | 0.0075 | 0.13 | 0.15 | 0.69 | 3.4E-08 |
|  |  | 2020-05-20 | 0.091 | 0.13 | 0.0076 | 0.12 | 0.15 | 0.70 | 6.5E-07 |
|  |  | 2020-05-27 | 0.100 | 0.14 | 0.0075 | 0.12 | 0.15 | 0.72 | 3.4E-07 |
|  |  | 2020-06-03 | 0.10 | 0.13 | 0.0075 | 0.12 | 0.15 | 0.76 | 3.8E-05 |
|  |  | 2020-06-10 | 0.10 | 0.14 | 0.0075 | 0.13 | 0.15 | 0.73 | 1.2E-06 |
|  |  | 2020-06-17 | 0.098 | 0.13 | 0.0075 | 0.12 | 0.15 | 0.74 | 7.8E-06 |
|  |  | 2020-06-24 | 0.11 | 0.14 | 0.0075 | 0.13 | 0.16 | 0.75 | 3.5E-06 |
|  |  | 2020-07-01 | 0.10 | 0.14 | 0.0075 | 0.12 | 0.15 | 0.73 | 1.5E-06 |
|  |  | 2020-07-08 | 0.098 | 0.13 | 0.0075 | 0.12 | 0.15 | 0.75 | 3.0E-05 |
|  |  | 2020-07-15 | 0.099 | 0.12 | 0.0075 | 0.11 | 0.14 | 0.81 | 0.0028 |
|  |  | 2020-07-22 | 0.094 | 0.12 | 0.0075 | 0.11 | 0.13 | 0.78 | 5.4E-04 |
|  |  | 2020-07-29 | 0.095 | 0.12 | 0.0075 | 0.10 | 0.13 | 0.81 | 0.0043 |
|  |  | 2020-08-05 | 0.096 | 0.12 | 0.0075 | 0.10 | 0.13 | 0.83 | 0.0075 |
|  |  | 2020-08-12 | 0.096 | 0.11 | 0.0075 | 0.099 | 0.13 | 0.84 | 0.0170 |
|  |  | 2020-08-19 | 0.10 | 0.12 | 0.0075 | 0.10 | 0.13 | 0.88 | 0.0665 |
|  |  | 2020-08-26 | 0.10 | 0.13 | 0.0075 | 0.11 | 0.14 | 0.82 | 0.0035 |
|  |  | 2020-09-02 | 0.10 | 0.13 | 0.0075 | 0.11 | 0.14 | 0.83 | 0.0042 |
|  |  | 2020-09-09 | 0.099 | 0.13 | 0.0075 | 0.11 | 0.14 | 0.78 | 1.8E-04 |
|  |  | 2020-09-16 | 0.11 | 0.13 | 0.0075 | 0.12 | 0.14 | 0.85 | 0.0096 |
|  |  | 2020-09-23 | 0.11 | 0.13 | 0.0075 | 0.11 | 0.14 | 0.82 | 0.0026 |
|  |  | 2020-09-30 | 0.10 | 0.13 | 0.0075 | 0.11 | 0.14 | 0.81 | 0.0018 |
|  |  | 2020-10-07 | 0.099 | 0.13 | 0.0075 | 0.12 | 0.15 | 0.75 | 2.3E-05 |
|  |  | 2020-10-14 | 0.11 | 0.14 | 0.0075 | 0.12 | 0.15 | 0.78 | 8.2E-05 |
|  |  | 2020-10-21 | 0.10 | 0.14 | 0.0075 | 0.12 | 0.15 | 0.76 | 2.7E-05 |
|  |  | 2020-10-28 | 0.10 | 0.14 | 0.0075 | 0.12 | 0.15 | 0.75 | 8.8E-06 |
|  |  | 2020-11-04 | 0.100 | 0.14 | 0.0075 | 0.12 | 0.15 | 0.72 | 3.9E-07 |
|  |  | 2020-11-11 | 0.10 | 0.14 | 0.0075 | 0.12 | 0.15 | 0.74 | 7.3E-06 |
|  |  | 2020-11-18 | 0.10 | 0.14 | 0.0075 | 0.13 | 0.16 | 0.71 | 2.2E-07 |
|  |  | 2020-11-25 | 0.097 | 0.14 | 0.0075 | 0.12 | 0.15 | 0.70 | 5.7E-08 |
|  |  | 2020-12-02 | 0.10 | 0.15 | 0.0075 | 0.13 | 0.16 | 0.72 | 1.5E-07 |
|  |  | 2020-12-09 | 0.10 | 0.15 | 0.0075 | 0.13 | 0.16 | 0.70 | 1.8E-08 |
|  |  | 2020-12-16 | 0.11 | 0.16 | 0.0076 | 0.15 | 0.18 | 0.66 | 1.7E-11 |
|  |  | 2020-12-23 | 0.085 | 0.13 | 0.0076 | 0.11 | 0.14 | 0.66 | 4.0E-08 |
| J01EE | Combinations of sulfonamides and trimethoprim, incl. derivatives | 2020-01-01 | 0.12 | 0.13 | 0.0073 | 0.12 | 0.15 | 0.90 | 0.0688 |
|  |  | 2020-01-08 | 0.17 | 0.16 | 0.0073 | 0.15 | 0.18 | 1.03 | 0.4812 |
|  |  | 2020-01-15 | 0.16 | 0.16 | 0.0073 | 0.14 | 0.17 | 1.03 | 0.4694 |
|  |  | 2020-01-22 | 0.16 | 0.16 | 0.0073 | 0.14 | 0.17 | 1.03 | 0.5502 |
|  |  | 2020-01-29 | 0.15 | 0.16 | 0.0073 | 0.14 | 0.17 | 0.98 | 0.5922 |
|  |  | 2020-02-05 | 0.16 | 0.16 | 0.0073 | 0.15 | 0.17 | 0.97 | 0.5535 |
|  |  | 2020-02-12 | 0.16 | 0.16 | 0.0073 | 0.14 | 0.17 | 1.01 | 0.7833 |
|  |  | 2020-02-19 | 0.16 | 0.16 | 0.0073 | 0.14 | 0.17 | 1.00 | 0.9975 |
|  |  | 2020-02-26 | 0.16 | 0.16 | 0.0073 | 0.14 | 0.17 | 1.05 | 0.2985 |
|  |  | 2020-03-04 | 0.17 | 0.16 | 0.0073 | 0.14 | 0.17 | 1.04 | 0.4185 |
|  |  | 2020-03-11 | 0.19 | 0.16 | 0.0074 | 0.14 | 0.17 | 1.17 | 3.8E-04 |
|  |  | 2020-03-18 | 0.16 | 0.16 | 0.0075 | 0.15 | 0.18 | 1.00 | 0.9830 |
|  |  | 2020-03-25 | 0.15 | 0.16 | 0.0079 | 0.14 | 0.17 | 0.92 | 0.0886 |
|  |  | 2020-04-01 | 0.14 | 0.16 | 0.0078 | 0.14 | 0.18 | 0.89 | 0.0297 |
|  |  | 2020-04-08 | 0.11 | 0.14 | 0.0078 | 0.12 | 0.15 | 0.78 | 2.3E-04 |
|  |  | 2020-04-15 | 0.14 | 0.16 | 0.0073 | 0.15 | 0.17 | 0.85 | 0.0017 |
|  |  | 2020-04-22 | 0.13 | 0.16 | 0.0073 | 0.15 | 0.18 | 0.80 | 1.4E-05 |
|  |  | 2020-04-29 | 0.12 | 0.15 | 0.0073 | 0.13 | 0.16 | 0.83 | 6.4E-04 |
|  |  | 2020-05-06 | 0.14 | 0.16 | 0.0073 | 0.14 | 0.17 | 0.86 | 0.0020 |
|  |  | 2020-05-13 | 0.14 | 0.16 | 0.0073 | 0.14 | 0.17 | 0.92 | 0.0910 |
|  |  | 2020-05-20 | 0.12 | 0.15 | 0.0074 | 0.13 | 0.16 | 0.84 | 0.0016 |
|  |  | 2020-05-27 | 0.13 | 0.17 | 0.0073 | 0.15 | 0.18 | 0.78 | 1.9E-06 |
|  |  | 2020-06-03 | 0.14 | 0.15 | 0.0073 | 0.14 | 0.16 | 0.93 | 0.1262 |
|  |  | 2020-06-10 | 0.14 | 0.16 | 0.0073 | 0.15 | 0.18 | 0.88 | 0.0068 |
|  |  | 2020-06-17 | 0.13 | 0.15 | 0.0073 | 0.13 | 0.16 | 0.86 | 0.0069 |
|  |  | 2020-06-24 | 0.13 | 0.16 | 0.0073 | 0.15 | 0.18 | 0.83 | 2.4E-04 |
|  |  | 2020-07-01 | 0.13 | 0.15 | 0.0073 | 0.14 | 0.17 | 0.87 | 0.0089 |
|  |  | 2020-07-08 | 0.13 | 0.15 | 0.0073 | 0.13 | 0.16 | 0.85 | 0.0027 |
|  |  | 2020-07-15 | 0.14 | 0.14 | 0.0073 | 0.13 | 0.16 | 0.96 | 0.4179 |
|  |  | 2020-07-22 | 0.13 | 0.14 | 0.0073 | 0.13 | 0.16 | 0.90 | 0.0589 |
|  |  | 2020-07-29 | 0.13 | 0.14 | 0.0073 | 0.13 | 0.16 | 0.91 | 0.0676 |
|  |  | 2020-08-05 | 0.13 | 0.15 | 0.0073 | 0.13 | 0.16 | 0.87 | 0.0100 |
|  |  | 2020-08-12 | 0.13 | 0.15 | 0.0073 | 0.14 | 0.16 | 0.88 | 0.0119 |
|  |  | 2020-08-19 | 0.14 | 0.16 | 0.0073 | 0.14 | 0.17 | 0.90 | 0.0435 |
|  |  | 2020-08-26 | 0.14 | 0.16 | 0.0073 | 0.15 | 0.17 | 0.90 | 0.0344 |
|  |  | 2020-09-02 | 0.15 | 0.16 | 0.0073 | 0.15 | 0.18 | 0.91 | 0.0489 |
|  |  | 2020-09-09 | 0.14 | 0.16 | 0.0073 | 0.15 | 0.18 | 0.89 | 0.0199 |
|  |  | 2020-09-16 | 0.15 | 0.16 | 0.0073 | 0.15 | 0.18 | 0.92 | 0.0865 |
|  |  | 2020-09-23 | 0.15 | 0.16 | 0.0073 | 0.15 | 0.18 | 0.95 | 0.2751 |
|  |  | 2020-09-30 | 0.16 | 0.16 | 0.0073 | 0.14 | 0.17 | 0.99 | 0.8005 |
|  |  | 2020-10-07 | 0.15 | 0.16 | 0.0073 | 0.15 | 0.18 | 0.92 | 0.0667 |
|  |  | 2020-10-14 | 0.15 | 0.16 | 0.0073 | 0.15 | 0.18 | 0.94 | 0.1620 |
|  |  | 2020-10-21 | 0.15 | 0.16 | 0.0073 | 0.15 | 0.18 | 0.90 | 0.0203 |
|  |  | 2020-10-28 | 0.14 | 0.16 | 0.0073 | 0.14 | 0.17 | 0.89 | 0.0177 |
|  |  | 2020-11-04 | 0.15 | 0.16 | 0.0073 | 0.15 | 0.18 | 0.95 | 0.2221 |
|  |  | 2020-11-11 | 0.15 | 0.17 | 0.0073 | 0.15 | 0.18 | 0.90 | 0.0284 |
|  |  | 2020-11-18 | 0.15 | 0.16 | 0.0073 | 0.15 | 0.18 | 0.91 | 0.0419 |
|  |  | 2020-11-25 | 0.14 | 0.16 | 0.0073 | 0.15 | 0.18 | 0.88 | 0.0088 |
|  |  | 2020-12-02 | 0.16 | 0.17 | 0.0073 | 0.15 | 0.18 | 0.97 | 0.4803 |
|  |  | 2020-12-09 | 0.15 | 0.17 | 0.0073 | 0.16 | 0.19 | 0.89 | 0.0113 |
|  |  | 2020-12-16 | 0.16 | 0.17 | 0.0074 | 0.16 | 0.19 | 0.94 | 0.1984 |
|  |  | 2020-12-23 | 0.11 | 0.13 | 0.0073 | 0.11 | 0.14 | 0.86 | 0.0177 |
| J01FA | Macrolides | 2020-01-01 | 0.12 | 0.13 | 0.013 | 0.10 | 0.15 | 0.94 | 0.5528 |
|  |  | 2020-01-08 | 0.17 | 0.16 | 0.013 | 0.13 | 0.19 | 1.05 | 0.5771 |
|  |  | 2020-01-15 | 0.16 | 0.16 | 0.013 | 0.14 | 0.19 | 0.98 | 0.8131 |
|  |  | 2020-01-22 | 0.17 | 0.17 | 0.013 | 0.14 | 0.20 | 1.01 | 0.8934 |
|  |  | 2020-01-29 | 0.18 | 0.18 | 0.013 | 0.15 | 0.20 | 1.01 | 0.8872 |
|  |  | 2020-02-05 | 0.17 | 0.18 | 0.013 | 0.16 | 0.21 | 0.95 | 0.5129 |
|  |  | 2020-02-12 | 0.18 | 0.18 | 0.013 | 0.15 | 0.20 | 1.00 | 0.9630 |
|  |  | 2020-02-19 | 0.19 | 0.18 | 0.013 | 0.15 | 0.20 | 1.05 | 0.4744 |
|  |  | 2020-02-26 | 0.18 | 0.17 | 0.013 | 0.15 | 0.20 | 1.05 | 0.5254 |
|  |  | 2020-03-04 | 0.18 | 0.16 | 0.013 | 0.14 | 0.19 | 1.10 | 0.2094 |
|  |  | 2020-03-11 | 0.19 | 0.16 | 0.013 | 0.13 | 0.19 | 1.21 | 0.0133 |
|  |  | 2020-03-18 | 0.20 | 0.16 | 0.013 | 0.13 | 0.19 | 1.25 | 0.0035 |
|  |  | 2020-03-25 | 0.18 | 0.16 | 0.014 | 0.13 | 0.18 | 1.17 | 0.0542 |
|  |  | 2020-04-01 | 0.16 | 0.15 | 0.014 | 0.13 | 0.18 | 1.06 | 0.5257 |
|  |  | 2020-04-08 | 0.10 | 0.13 | 0.014 | 0.099 | 0.15 | 0.81 | 0.0853 |
|  |  | 2020-04-15 | 0.13 | 0.16 | 0.013 | 0.13 | 0.18 | 0.81 | 0.0241 |
|  |  | 2020-04-22 | 0.12 | 0.15 | 0.013 | 0.13 | 0.18 | 0.78 | 0.0123 |
|  |  | 2020-04-29 | 0.10 | 0.13 | 0.013 | 0.11 | 0.16 | 0.75 | 0.0131 |
|  |  | 2020-05-06 | 0.11 | 0.15 | 0.013 | 0.13 | 0.18 | 0.73 | 0.0024 |
|  |  | 2020-05-13 | 0.12 | 0.15 | 0.013 | 0.12 | 0.17 | 0.82 | 0.0470 |
|  |  | 2020-05-20 | 0.11 | 0.13 | 0.013 | 0.10 | 0.16 | 0.82 | 0.0808 |
|  |  | 2020-05-27 | 0.12 | 0.15 | 0.013 | 0.12 | 0.17 | 0.82 | 0.0454 |
|  |  | 2020-06-03 | 0.11 | 0.13 | 0.013 | 0.11 | 0.16 | 0.85 | 0.1267 |
|  |  | 2020-06-10 | 0.12 | 0.15 | 0.013 | 0.12 | 0.18 | 0.80 | 0.0258 |
|  |  | 2020-06-17 | 0.10 | 0.13 | 0.013 | 0.10 | 0.15 | 0.81 | 0.0604 |
|  |  | 2020-06-24 | 0.12 | 0.15 | 0.013 | 0.12 | 0.17 | 0.80 | 0.0260 |
|  |  | 2020-07-01 | 0.12 | 0.14 | 0.013 | 0.11 | 0.16 | 0.84 | 0.1029 |
|  |  | 2020-07-08 | 0.10 | 0.13 | 0.013 | 0.10 | 0.15 | 0.82 | 0.0802 |
|  |  | 2020-07-15 | 0.10 | 0.12 | 0.013 | 0.093 | 0.14 | 0.85 | 0.1612 |
|  |  | 2020-07-22 | 0.098 | 0.11 | 0.013 | 0.088 | 0.14 | 0.86 | 0.2334 |
|  |  | 2020-07-29 | 0.094 | 0.12 | 0.013 | 0.092 | 0.14 | 0.80 | 0.0762 |
|  |  | 2020-08-05 | 0.10 | 0.12 | 0.013 | 0.092 | 0.14 | 0.86 | 0.2213 |
|  |  | 2020-08-12 | 0.10 | 0.12 | 0.013 | 0.094 | 0.15 | 0.85 | 0.1734 |
|  |  | 2020-08-19 | 0.12 | 0.12 | 0.013 | 0.099 | 0.15 | 0.94 | 0.5484 |
|  |  | 2020-08-26 | 0.12 | 0.14 | 0.013 | 0.11 | 0.16 | 0.89 | 0.2660 |
|  |  | 2020-09-02 | 0.12 | 0.14 | 0.013 | 0.11 | 0.16 | 0.88 | 0.2190 |
|  |  | 2020-09-09 | 0.11 | 0.14 | 0.013 | 0.12 | 0.17 | 0.79 | 0.0211 |
|  |  | 2020-09-16 | 0.12 | 0.14 | 0.013 | 0.12 | 0.17 | 0.85 | 0.0918 |
|  |  | 2020-09-23 | 0.12 | 0.15 | 0.013 | 0.12 | 0.17 | 0.81 | 0.0332 |
|  |  | 2020-09-30 | 0.13 | 0.15 | 0.013 | 0.12 | 0.17 | 0.86 | 0.1183 |
|  |  | 2020-10-07 | 0.12 | 0.15 | 0.013 | 0.12 | 0.17 | 0.81 | 0.0321 |
|  |  | 2020-10-14 | 0.12 | 0.15 | 0.013 | 0.13 | 0.18 | 0.81 | 0.0306 |
|  |  | 2020-10-21 | 0.12 | 0.15 | 0.013 | 0.13 | 0.18 | 0.78 | 0.0113 |
|  |  | 2020-10-28 | 0.12 | 0.15 | 0.013 | 0.12 | 0.17 | 0.82 | 0.0415 |
|  |  | 2020-11-04 | 0.12 | 0.16 | 0.013 | 0.13 | 0.18 | 0.79 | 0.0125 |
|  |  | 2020-11-11 | 0.13 | 0.15 | 0.013 | 0.13 | 0.18 | 0.86 | 0.1053 |
|  |  | 2020-11-18 | 0.12 | 0.15 | 0.013 | 0.13 | 0.18 | 0.80 | 0.0165 |
|  |  | 2020-11-25 | 0.12 | 0.16 | 0.013 | 0.13 | 0.18 | 0.78 | 0.0082 |
|  |  | 2020-12-02 | 0.13 | 0.16 | 0.013 | 0.14 | 0.19 | 0.82 | 0.0270 |
|  |  | 2020-12-09 | 0.13 | 0.17 | 0.013 | 0.14 | 0.19 | 0.78 | 0.0046 |
|  |  | 2020-12-16 | 0.14 | 0.18 | 0.013 | 0.15 | 0.21 | 0.77 | 0.0017 |
|  |  | 2020-12-23 | 0.083 | 0.11 | 0.013 | 0.088 | 0.14 | 0.73 | 0.0189 |
| J01FF | Lincosamides | 2020-01-01 | 0.18 | 0.20 | 0.012 | 0.17 | 0.22 | 0.92 | 0.1946 |
|  |  | 2020-01-08 | 0.23 | 0.25 | 0.012 | 0.22 | 0.27 | 0.95 | 0.2940 |
|  |  | 2020-01-15 | 0.24 | 0.25 | 0.012 | 0.23 | 0.27 | 0.96 | 0.3662 |
|  |  | 2020-01-22 | 0.23 | 0.25 | 0.012 | 0.23 | 0.28 | 0.90 | 0.0380 |
|  |  | 2020-01-29 | 0.24 | 0.25 | 0.012 | 0.23 | 0.27 | 0.96 | 0.3975 |
|  |  | 2020-02-05 | 0.23 | 0.25 | 0.012 | 0.23 | 0.27 | 0.92 | 0.1017 |
|  |  | 2020-02-12 | 0.23 | 0.25 | 0.012 | 0.23 | 0.28 | 0.91 | 0.0501 |
|  |  | 2020-02-19 | 0.23 | 0.25 | 0.012 | 0.23 | 0.27 | 0.92 | 0.0777 |
|  |  | 2020-02-26 | 0.24 | 0.25 | 0.012 | 0.23 | 0.28 | 0.94 | 0.1703 |
|  |  | 2020-03-04 | 0.24 | 0.25 | 0.012 | 0.23 | 0.28 | 0.96 | 0.3688 |
|  |  | 2020-03-11 | 0.26 | 0.25 | 0.012 | 0.23 | 0.28 | 1.00 | 0.9340 |
|  |  | 2020-03-18 | 0.23 | 0.26 | 0.012 | 0.24 | 0.29 | 0.88 | 0.0101 |
|  |  | 2020-03-25 | 0.22 | 0.27 | 0.013 | 0.24 | 0.29 | 0.83 | 7.4E-04 |
|  |  | 2020-04-01 | 0.22 | 0.26 | 0.013 | 0.24 | 0.29 | 0.83 | 5.1E-04 |
|  |  | 2020-04-08 | 0.17 | 0.21 | 0.013 | 0.19 | 0.24 | 0.79 | 6.7E-04 |
|  |  | 2020-04-15 | 0.20 | 0.26 | 0.012 | 0.24 | 0.29 | 0.77 | 9.9E-07 |
|  |  | 2020-04-22 | 0.21 | 0.27 | 0.012 | 0.24 | 0.29 | 0.77 | 1.1E-06 |
|  |  | 2020-04-29 | 0.19 | 0.24 | 0.012 | 0.22 | 0.26 | 0.79 | 5.4E-05 |
|  |  | 2020-05-06 | 0.19 | 0.26 | 0.012 | 0.24 | 0.28 | 0.75 | 1.2E-07 |
|  |  | 2020-05-13 | 0.20 | 0.26 | 0.012 | 0.24 | 0.28 | 0.77 | 1.9E-06 |
|  |  | 2020-05-20 | 0.18 | 0.23 | 0.012 | 0.21 | 0.25 | 0.80 | 1.3E-04 |
|  |  | 2020-05-27 | 0.20 | 0.26 | 0.012 | 0.24 | 0.29 | 0.75 | 1.1E-07 |
|  |  | 2020-06-03 | 0.21 | 0.24 | 0.012 | 0.22 | 0.26 | 0.86 | 0.0060 |
|  |  | 2020-06-10 | 0.21 | 0.26 | 0.012 | 0.24 | 0.29 | 0.81 | 3.5E-05 |
|  |  | 2020-06-17 | 0.20 | 0.24 | 0.012 | 0.21 | 0.26 | 0.83 | 7.8E-04 |
|  |  | 2020-06-24 | 0.22 | 0.27 | 0.012 | 0.24 | 0.29 | 0.82 | 1.0E-04 |
|  |  | 2020-07-01 | 0.22 | 0.26 | 0.012 | 0.23 | 0.28 | 0.84 | 7.6E-04 |
|  |  | 2020-07-08 | 0.20 | 0.24 | 0.012 | 0.22 | 0.26 | 0.82 | 4.1E-04 |
|  |  | 2020-07-15 | 0.20 | 0.23 | 0.012 | 0.21 | 0.26 | 0.85 | 0.0036 |
|  |  | 2020-07-22 | 0.20 | 0.23 | 0.012 | 0.21 | 0.25 | 0.84 | 0.0028 |
|  |  | 2020-07-29 | 0.20 | 0.23 | 0.012 | 0.21 | 0.25 | 0.87 | 0.0147 |
|  |  | 2020-08-05 | 0.20 | 0.23 | 0.012 | 0.21 | 0.25 | 0.85 | 0.0050 |
|  |  | 2020-08-12 | 0.20 | 0.24 | 0.012 | 0.21 | 0.26 | 0.84 | 0.0015 |
|  |  | 2020-08-19 | 0.22 | 0.24 | 0.012 | 0.22 | 0.26 | 0.91 | 0.0676 |
|  |  | 2020-08-26 | 0.21 | 0.24 | 0.012 | 0.22 | 0.26 | 0.87 | 0.0091 |
|  |  | 2020-09-02 | 0.21 | 0.25 | 0.012 | 0.22 | 0.27 | 0.84 | 0.0015 |
|  |  | 2020-09-09 | 0.21 | 0.24 | 0.012 | 0.22 | 0.27 | 0.86 | 0.0052 |
|  |  | 2020-09-16 | 0.21 | 0.24 | 0.012 | 0.22 | 0.27 | 0.84 | 0.0015 |
|  |  | 2020-09-23 | 0.22 | 0.24 | 0.012 | 0.22 | 0.27 | 0.88 | 0.0182 |
|  |  | 2020-09-30 | 0.21 | 0.24 | 0.012 | 0.22 | 0.27 | 0.87 | 0.0086 |
|  |  | 2020-10-07 | 0.21 | 0.24 | 0.012 | 0.22 | 0.27 | 0.88 | 0.0135 |
|  |  | 2020-10-14 | 0.21 | 0.25 | 0.012 | 0.22 | 0.27 | 0.85 | 0.0027 |
|  |  | 2020-10-21 | 0.22 | 0.25 | 0.012 | 0.22 | 0.27 | 0.88 | 0.0118 |
|  |  | 2020-10-28 | 0.20 | 0.23 | 0.012 | 0.21 | 0.25 | 0.88 | 0.0253 |
|  |  | 2020-11-04 | 0.20 | 0.25 | 0.012 | 0.22 | 0.27 | 0.83 | 4.4E-04 |
|  |  | 2020-11-11 | 0.20 | 0.24 | 0.012 | 0.22 | 0.26 | 0.84 | 0.0014 |
|  |  | 2020-11-18 | 0.21 | 0.24 | 0.012 | 0.22 | 0.27 | 0.86 | 0.0039 |
|  |  | 2020-11-25 | 0.19 | 0.24 | 0.012 | 0.22 | 0.27 | 0.80 | 4.7E-05 |
|  |  | 2020-12-02 | 0.21 | 0.25 | 0.012 | 0.22 | 0.27 | 0.85 | 0.0025 |
|  |  | 2020-12-09 | 0.21 | 0.25 | 0.012 | 0.23 | 0.28 | 0.85 | 0.0018 |
|  |  | 2020-12-16 | 0.23 | 0.27 | 0.012 | 0.25 | 0.30 | 0.83 | 2.6E-04 |
|  |  | 2020-12-23 | 0.16 | 0.19 | 0.012 | 0.16 | 0.21 | 0.85 | 0.0214 |
| J01MA | Fluoroquinolones | 2020-01-01 | 0.28 | 0.28 | 0.015 | 0.25 | 0.31 | 0.99 | 0.9063 |
|  |  | 2020-01-08 | 0.38 | 0.37 | 0.015 | 0.34 | 0.40 | 1.05 | 0.2785 |
|  |  | 2020-01-15 | 0.36 | 0.37 | 0.015 | 0.34 | 0.40 | 1.00 | 0.9529 |
|  |  | 2020-01-22 | 0.36 | 0.36 | 0.015 | 0.33 | 0.39 | 0.99 | 0.8031 |
|  |  | 2020-01-29 | 0.34 | 0.35 | 0.015 | 0.32 | 0.38 | 0.97 | 0.4783 |
|  |  | 2020-02-05 | 0.34 | 0.35 | 0.015 | 0.32 | 0.38 | 0.97 | 0.5209 |
|  |  | 2020-02-12 | 0.33 | 0.35 | 0.015 | 0.32 | 0.38 | 0.96 | 0.3495 |
|  |  | 2020-02-19 | 0.34 | 0.34 | 0.015 | 0.31 | 0.37 | 0.98 | 0.6447 |
|  |  | 2020-02-26 | 0.34 | 0.34 | 0.015 | 0.31 | 0.37 | 0.99 | 0.9088 |
|  |  | 2020-03-04 | 0.33 | 0.35 | 0.015 | 0.32 | 0.38 | 0.95 | 0.2825 |
|  |  | 2020-03-11 | 0.34 | 0.35 | 0.016 | 0.32 | 0.38 | 0.97 | 0.5067 |
|  |  | 2020-03-18 | 0.31 | 0.35 | 0.016 | 0.32 | 0.38 | 0.89 | 0.0159 |
|  |  | 2020-03-25 | 0.30 | 0.34 | 0.016 | 0.31 | 0.37 | 0.89 | 0.0199 |
|  |  | 2020-04-01 | 0.31 | 0.35 | 0.016 | 0.32 | 0.38 | 0.88 | 0.0099 |
|  |  | 2020-04-08 | 0.23 | 0.27 | 0.016 | 0.24 | 0.30 | 0.87 | 0.0365 |
|  |  | 2020-04-15 | 0.30 | 0.34 | 0.015 | 0.31 | 0.37 | 0.87 | 0.0032 |
|  |  | 2020-04-22 | 0.29 | 0.34 | 0.015 | 0.31 | 0.37 | 0.85 | 9.4E-04 |
|  |  | 2020-04-29 | 0.26 | 0.31 | 0.015 | 0.28 | 0.34 | 0.85 | 0.0020 |
|  |  | 2020-05-06 | 0.30 | 0.33 | 0.015 | 0.30 | 0.36 | 0.89 | 0.0143 |
|  |  | 2020-05-13 | 0.28 | 0.33 | 0.015 | 0.30 | 0.36 | 0.85 | 0.0012 |
|  |  | 2020-05-20 | 0.27 | 0.30 | 0.015 | 0.26 | 0.33 | 0.92 | 0.1115 |
|  |  | 2020-05-27 | 0.28 | 0.34 | 0.015 | 0.31 | 0.37 | 0.83 | 2.1E-04 |
|  |  | 2020-06-03 | 0.29 | 0.31 | 0.015 | 0.28 | 0.34 | 0.93 | 0.1520 |
|  |  | 2020-06-10 | 0.30 | 0.35 | 0.015 | 0.32 | 0.38 | 0.86 | 0.0014 |
|  |  | 2020-06-17 | 0.27 | 0.32 | 0.015 | 0.29 | 0.35 | 0.85 | 0.0026 |
|  |  | 2020-06-24 | 0.30 | 0.37 | 0.015 | 0.34 | 0.40 | 0.81 | 7.7E-06 |
|  |  | 2020-07-01 | 0.32 | 0.34 | 0.015 | 0.31 | 0.37 | 0.93 | 0.1328 |
|  |  | 2020-07-08 | 0.32 | 0.33 | 0.015 | 0.30 | 0.36 | 0.97 | 0.4720 |
|  |  | 2020-07-15 | 0.30 | 0.33 | 0.015 | 0.30 | 0.36 | 0.92 | 0.0715 |
|  |  | 2020-07-22 | 0.30 | 0.32 | 0.015 | 0.29 | 0.35 | 0.95 | 0.2528 |
|  |  | 2020-07-29 | 0.31 | 0.34 | 0.015 | 0.31 | 0.37 | 0.93 | 0.1061 |
|  |  | 2020-08-05 | 0.31 | 0.35 | 0.015 | 0.32 | 0.38 | 0.88 | 0.0055 |
|  |  | 2020-08-12 | 0.30 | 0.38 | 0.015 | 0.35 | 0.41 | 0.80 | 1.2E-06 |
|  |  | 2020-08-19 | 0.34 | 0.37 | 0.015 | 0.34 | 0.40 | 0.92 | 0.0491 |
|  |  | 2020-08-26 | 0.33 | 0.37 | 0.015 | 0.34 | 0.40 | 0.89 | 0.0089 |
|  |  | 2020-09-02 | 0.34 | 0.38 | 0.015 | 0.35 | 0.41 | 0.91 | 0.0202 |
|  |  | 2020-09-09 | 0.33 | 0.37 | 0.015 | 0.34 | 0.40 | 0.89 | 0.0093 |
|  |  | 2020-09-16 | 0.34 | 0.37 | 0.015 | 0.34 | 0.40 | 0.92 | 0.0552 |
|  |  | 2020-09-23 | 0.33 | 0.37 | 0.015 | 0.34 | 0.40 | 0.90 | 0.0122 |
|  |  | 2020-09-30 | 0.33 | 0.37 | 0.015 | 0.34 | 0.40 | 0.91 | 0.0254 |
|  |  | 2020-10-07 | 0.33 | 0.36 | 0.015 | 0.33 | 0.39 | 0.92 | 0.0462 |
|  |  | 2020-10-14 | 0.34 | 0.37 | 0.015 | 0.34 | 0.40 | 0.91 | 0.0227 |
|  |  | 2020-10-21 | 0.32 | 0.36 | 0.015 | 0.33 | 0.39 | 0.89 | 0.0106 |
|  |  | 2020-10-28 | 0.33 | 0.34 | 0.015 | 0.31 | 0.37 | 0.95 | 0.2898 |
|  |  | 2020-11-04 | 0.32 | 0.36 | 0.015 | 0.33 | 0.39 | 0.88 | 0.0060 |
|  |  | 2020-11-11 | 0.32 | 0.35 | 0.015 | 0.32 | 0.38 | 0.91 | 0.0486 |
|  |  | 2020-11-18 | 0.32 | 0.34 | 0.015 | 0.31 | 0.37 | 0.93 | 0.0939 |
|  |  | 2020-11-25 | 0.31 | 0.34 | 0.015 | 0.31 | 0.37 | 0.90 | 0.0317 |
|  |  | 2020-12-02 | 0.32 | 0.35 | 0.015 | 0.32 | 0.38 | 0.91 | 0.0357 |
|  |  | 2020-12-09 | 0.33 | 0.36 | 0.015 | 0.33 | 0.39 | 0.92 | 0.0689 |
|  |  | 2020-12-16 | 0.34 | 0.38 | 0.015 | 0.34 | 0.41 | 0.91 | 0.0389 |
|  |  | 2020-12-23 | 0.25 | 0.25 | 0.015 | 0.22 | 0.28 | 1.00 | 0.9744 |
| J01XE | Nitrofuran derivatives | 2020-01-01 | 0.40 | 0.42 | 0.018 | 0.38 | 0.45 | 0.96 | 0.3617 |
|  |  | 2020-01-08 | 0.55 | 0.51 | 0.017 | 0.47 | 0.54 | 1.08 | 0.0175 |
|  |  | 2020-01-15 | 0.51 | 0.50 | 0.017 | 0.47 | 0.54 | 1.02 | 0.5141 |
|  |  | 2020-01-22 | 0.52 | 0.51 | 0.017 | 0.47 | 0.54 | 1.03 | 0.3555 |
|  |  | 2020-01-29 | 0.51 | 0.49 | 0.017 | 0.45 | 0.52 | 1.04 | 0.2153 |
|  |  | 2020-02-05 | 0.49 | 0.49 | 0.017 | 0.45 | 0.52 | 1.01 | 0.8258 |
|  |  | 2020-02-12 | 0.50 | 0.48 | 0.017 | 0.45 | 0.52 | 1.03 | 0.3514 |
|  |  | 2020-02-19 | 0.48 | 0.48 | 0.017 | 0.45 | 0.52 | 1.00 | 0.8990 |
|  |  | 2020-02-26 | 0.47 | 0.48 | 0.017 | 0.45 | 0.52 | 0.98 | 0.6189 |
|  |  | 2020-03-04 | 0.48 | 0.48 | 0.017 | 0.45 | 0.52 | 0.99 | 0.8189 |
|  |  | 2020-03-11 | 0.49 | 0.48 | 0.018 | 0.44 | 0.51 | 1.02 | 0.6709 |
|  |  | 2020-03-18 | 0.47 | 0.48 | 0.018 | 0.45 | 0.52 | 0.98 | 0.6251 |
|  |  | 2020-03-25 | 0.46 | 0.49 | 0.019 | 0.45 | 0.52 | 0.95 | 0.1701 |
|  |  | 2020-04-01 | 0.45 | 0.50 | 0.019 | 0.46 | 0.53 | 0.90 | 0.0104 |
|  |  | 2020-04-08 | 0.36 | 0.39 | 0.019 | 0.35 | 0.42 | 0.93 | 0.1652 |
|  |  | 2020-04-15 | 0.44 | 0.49 | 0.018 | 0.45 | 0.52 | 0.91 | 0.0139 |
|  |  | 2020-04-22 | 0.44 | 0.49 | 0.017 | 0.46 | 0.53 | 0.90 | 0.0041 |
|  |  | 2020-04-29 | 0.40 | 0.45 | 0.018 | 0.42 | 0.49 | 0.89 | 0.0044 |
|  |  | 2020-05-06 | 0.44 | 0.48 | 0.017 | 0.45 | 0.52 | 0.92 | 0.0269 |
|  |  | 2020-05-13 | 0.46 | 0.48 | 0.017 | 0.45 | 0.52 | 0.94 | 0.1255 |
|  |  | 2020-05-20 | 0.39 | 0.43 | 0.018 | 0.40 | 0.47 | 0.91 | 0.0217 |
|  |  | 2020-05-27 | 0.43 | 0.49 | 0.017 | 0.45 | 0.52 | 0.88 | 0.0013 |
|  |  | 2020-06-03 | 0.44 | 0.45 | 0.017 | 0.42 | 0.49 | 0.96 | 0.3398 |
|  |  | 2020-06-10 | 0.43 | 0.51 | 0.017 | 0.47 | 0.54 | 0.85 | 3.7E-05 |
|  |  | 2020-06-17 | 0.40 | 0.46 | 0.017 | 0.43 | 0.50 | 0.88 | 0.0012 |
|  |  | 2020-06-24 | 0.45 | 0.53 | 0.017 | 0.49 | 0.56 | 0.86 | 4.6E-05 |
|  |  | 2020-07-01 | 0.49 | 0.50 | 0.017 | 0.47 | 0.54 | 0.98 | 0.5824 |
|  |  | 2020-07-08 | 0.48 | 0.50 | 0.017 | 0.47 | 0.53 | 0.96 | 0.2408 |
|  |  | 2020-07-15 | 0.47 | 0.50 | 0.017 | 0.46 | 0.53 | 0.95 | 0.1908 |
|  |  | 2020-07-22 | 0.48 | 0.49 | 0.017 | 0.45 | 0.52 | 0.98 | 0.4994 |
|  |  | 2020-07-29 | 0.49 | 0.51 | 0.017 | 0.47 | 0.54 | 0.96 | 0.2798 |
|  |  | 2020-08-05 | 0.47 | 0.52 | 0.017 | 0.49 | 0.55 | 0.90 | 0.0046 |
|  |  | 2020-08-12 | 0.48 | 0.54 | 0.017 | 0.51 | 0.57 | 0.88 | 2.9E-04 |
|  |  | 2020-08-19 | 0.50 | 0.55 | 0.017 | 0.52 | 0.59 | 0.90 | 0.0028 |
|  |  | 2020-08-26 | 0.51 | 0.55 | 0.017 | 0.52 | 0.59 | 0.92 | 0.0093 |
|  |  | 2020-09-02 | 0.52 | 0.56 | 0.017 | 0.52 | 0.59 | 0.93 | 0.0238 |
|  |  | 2020-09-09 | 0.53 | 0.56 | 0.017 | 0.53 | 0.59 | 0.95 | 0.1287 |
|  |  | 2020-09-16 | 0.54 | 0.57 | 0.017 | 0.53 | 0.60 | 0.95 | 0.0912 |
|  |  | 2020-09-23 | 0.54 | 0.56 | 0.017 | 0.52 | 0.59 | 0.97 | 0.4183 |
|  |  | 2020-09-30 | 0.56 | 0.55 | 0.017 | 0.51 | 0.58 | 1.01 | 0.7212 |
|  |  | 2020-10-07 | 0.54 | 0.55 | 0.017 | 0.51 | 0.58 | 0.99 | 0.6848 |
|  |  | 2020-10-14 | 0.52 | 0.55 | 0.017 | 0.51 | 0.58 | 0.96 | 0.2083 |
|  |  | 2020-10-21 | 0.51 | 0.55 | 0.017 | 0.51 | 0.58 | 0.94 | 0.0619 |
|  |  | 2020-10-28 | 0.52 | 0.52 | 0.017 | 0.48 | 0.55 | 1.00 | 0.9704 |
|  |  | 2020-11-04 | 0.51 | 0.53 | 0.017 | 0.50 | 0.57 | 0.97 | 0.2978 |
|  |  | 2020-11-11 | 0.53 | 0.52 | 0.017 | 0.49 | 0.56 | 1.01 | 0.8072 |
|  |  | 2020-11-18 | 0.53 | 0.52 | 0.017 | 0.49 | 0.56 | 1.01 | 0.6992 |
|  |  | 2020-11-25 | 0.51 | 0.52 | 0.017 | 0.48 | 0.55 | 0.98 | 0.5134 |
|  |  | 2020-12-02 | 0.52 | 0.53 | 0.017 | 0.49 | 0.56 | 0.99 | 0.8395 |
|  |  | 2020-12-09 | 0.53 | 0.54 | 0.017 | 0.51 | 0.58 | 0.98 | 0.5618 |
|  |  | 2020-12-16 | 0.56 | 0.56 | 0.018 | 0.53 | 0.59 | 1.00 | 0.8943 |
|  |  | 2020-12-23 | 0.42 | 0.39 | 0.018 | 0.35 | 0.42 | 1.10 | 0.0277 |
| J05 | Antivirals for systemic use | 2020-01-01 | 0.61 | 0.66 | 0.026 | 0.61 | 0.71 | 0.93 | 0.0968 |
|  |  | 2020-01-08 | 0.83 | 0.80 | 0.026 | 0.75 | 0.85 | 1.04 | 0.2135 |
|  |  | 2020-01-15 | 0.79 | 0.78 | 0.026 | 0.73 | 0.84 | 1.00 | 0.9224 |
|  |  | 2020-01-22 | 0.81 | 0.81 | 0.026 | 0.76 | 0.86 | 1.00 | 0.9897 |
|  |  | 2020-01-29 | 0.78 | 0.80 | 0.026 | 0.75 | 0.85 | 0.97 | 0.3850 |
|  |  | 2020-02-05 | 0.79 | 0.81 | 0.026 | 0.75 | 0.86 | 0.98 | 0.5503 |
|  |  | 2020-02-12 | 0.76 | 0.80 | 0.026 | 0.75 | 0.85 | 0.95 | 0.1322 |
|  |  | 2020-02-19 | 0.78 | 0.80 | 0.026 | 0.75 | 0.86 | 0.97 | 0.3235 |
|  |  | 2020-02-26 | 0.86 | 0.81 | 0.026 | 0.75 | 0.86 | 1.06 | 0.0602 |
|  |  | 2020-03-04 | 0.87 | 0.79 | 0.026 | 0.74 | 0.84 | 1.10 | 0.0024 |
|  |  | 2020-03-11 | 1.0 | 0.77 | 0.027 | 0.72 | 0.83 | 1.29 | 3.1E-15 |
|  |  | 2020-03-18 | 0.87 | 0.79 | 0.027 | 0.73 | 0.84 | 1.10 | 0.0039 |
|  |  | 2020-03-25 | 0.79 | 0.79 | 0.028 | 0.74 | 0.85 | 1.00 | 0.9486 |
|  |  | 2020-04-01 | 0.76 | 0.80 | 0.028 | 0.75 | 0.86 | 0.94 | 0.1114 |
|  |  | 2020-04-08 | 0.57 | 0.63 | 0.028 | 0.58 | 0.69 | 0.91 | 0.0366 |
|  |  | 2020-04-15 | 0.71 | 0.79 | 0.026 | 0.74 | 0.84 | 0.90 | 0.0037 |
|  |  | 2020-04-22 | 0.72 | 0.80 | 0.026 | 0.75 | 0.85 | 0.90 | 0.0023 |
|  |  | 2020-04-29 | 0.66 | 0.74 | 0.026 | 0.69 | 0.79 | 0.89 | 0.0024 |
|  |  | 2020-05-06 | 0.72 | 0.78 | 0.026 | 0.73 | 0.83 | 0.92 | 0.0120 |
|  |  | 2020-05-13 | 0.72 | 0.77 | 0.026 | 0.71 | 0.82 | 0.94 | 0.0628 |
|  |  | 2020-05-20 | 0.66 | 0.72 | 0.026 | 0.67 | 0.78 | 0.91 | 0.0150 |
|  |  | 2020-05-27 | 0.72 | 0.79 | 0.026 | 0.74 | 0.85 | 0.91 | 0.0054 |
|  |  | 2020-06-03 | 0.74 | 0.75 | 0.026 | 0.70 | 0.80 | 0.98 | 0.5636 |
|  |  | 2020-06-10 | 0.74 | 0.82 | 0.026 | 0.77 | 0.87 | 0.90 | 0.0027 |
|  |  | 2020-06-17 | 0.67 | 0.74 | 0.026 | 0.69 | 0.79 | 0.90 | 0.0041 |
|  |  | 2020-06-24 | 0.76 | 0.84 | 0.026 | 0.79 | 0.89 | 0.91 | 0.0027 |
|  |  | 2020-07-01 | 0.76 | 0.79 | 0.026 | 0.74 | 0.84 | 0.97 | 0.3145 |
|  |  | 2020-07-08 | 0.71 | 0.74 | 0.026 | 0.69 | 0.79 | 0.95 | 0.1922 |
|  |  | 2020-07-15 | 0.69 | 0.72 | 0.026 | 0.66 | 0.77 | 0.96 | 0.2929 |
|  |  | 2020-07-22 | 0.70 | 0.71 | 0.026 | 0.66 | 0.76 | 0.99 | 0.8709 |
|  |  | 2020-07-29 | 0.68 | 0.70 | 0.026 | 0.65 | 0.76 | 0.97 | 0.4415 |
|  |  | 2020-08-05 | 0.69 | 0.72 | 0.026 | 0.66 | 0.77 | 0.97 | 0.3909 |
|  |  | 2020-08-12 | 0.70 | 0.73 | 0.026 | 0.68 | 0.78 | 0.95 | 0.1919 |
|  |  | 2020-08-19 | 0.75 | 0.74 | 0.026 | 0.69 | 0.79 | 1.01 | 0.6853 |
|  |  | 2020-08-26 | 0.73 | 0.76 | 0.026 | 0.71 | 0.82 | 0.95 | 0.1878 |
|  |  | 2020-09-02 | 0.72 | 0.75 | 0.026 | 0.70 | 0.80 | 0.96 | 0.2238 |
|  |  | 2020-09-09 | 0.72 | 0.74 | 0.026 | 0.69 | 0.79 | 0.98 | 0.4897 |
|  |  | 2020-09-16 | 0.71 | 0.74 | 0.026 | 0.69 | 0.79 | 0.95 | 0.1532 |
|  |  | 2020-09-23 | 0.74 | 0.77 | 0.026 | 0.72 | 0.82 | 0.96 | 0.2118 |
|  |  | 2020-09-30 | 0.74 | 0.76 | 0.026 | 0.70 | 0.81 | 0.97 | 0.4196 |
|  |  | 2020-10-07 | 0.71 | 0.75 | 0.026 | 0.70 | 0.80 | 0.94 | 0.1073 |
|  |  | 2020-10-14 | 0.75 | 0.75 | 0.026 | 0.70 | 0.80 | 1.00 | 0.9255 |
|  |  | 2020-10-21 | 0.77 | 0.78 | 0.026 | 0.72 | 0.83 | 0.99 | 0.6824 |
|  |  | 2020-10-28 | 0.73 | 0.76 | 0.026 | 0.71 | 0.81 | 0.97 | 0.3594 |
|  |  | 2020-11-04 | 0.75 | 0.78 | 0.026 | 0.73 | 0.83 | 0.95 | 0.1747 |
|  |  | 2020-11-11 | 0.74 | 0.76 | 0.026 | 0.71 | 0.81 | 0.97 | 0.3866 |
|  |  | 2020-11-18 | 0.74 | 0.78 | 0.026 | 0.73 | 0.83 | 0.95 | 0.1565 |
|  |  | 2020-11-25 | 0.78 | 0.81 | 0.026 | 0.76 | 0.86 | 0.97 | 0.3086 |
|  |  | 2020-12-02 | 0.79 | 0.82 | 0.026 | 0.77 | 0.87 | 0.97 | 0.2902 |
|  |  | 2020-12-09 | 0.80 | 0.84 | 0.026 | 0.79 | 0.89 | 0.95 | 0.1220 |
|  |  | 2020-12-16 | 0.83 | 0.87 | 0.026 | 0.82 | 0.92 | 0.95 | 0.1164 |
|  |  | 2020-12-23 | 0.61 | 0.62 | 0.026 | 0.57 | 0.67 | 0.98 | 0.6228 |
| J05AB | Nucleosides and nucleotides excl. reverse transcriptase inhibitors | 2020-01-01 | 0.50 | 0.53 | 0.018 | 0.50 | 0.57 | 0.95 | 0.1362 |
|  |  | 2020-01-08 | 0.66 | 0.63 | 0.018 | 0.60 | 0.67 | 1.04 | 0.1436 |
|  |  | 2020-01-15 | 0.63 | 0.62 | 0.018 | 0.59 | 0.66 | 1.01 | 0.7339 |
|  |  | 2020-01-22 | 0.64 | 0.64 | 0.018 | 0.61 | 0.68 | 1.00 | 0.9315 |
|  |  | 2020-01-29 | 0.62 | 0.63 | 0.018 | 0.59 | 0.66 | 0.99 | 0.6569 |
|  |  | 2020-02-05 | 0.63 | 0.62 | 0.018 | 0.59 | 0.66 | 1.01 | 0.8495 |
|  |  | 2020-02-12 | 0.60 | 0.61 | 0.018 | 0.57 | 0.64 | 0.99 | 0.7101 |
|  |  | 2020-02-19 | 0.61 | 0.62 | 0.018 | 0.58 | 0.65 | 0.99 | 0.6860 |
|  |  | 2020-02-26 | 0.66 | 0.63 | 0.018 | 0.59 | 0.66 | 1.05 | 0.0771 |
|  |  | 2020-03-04 | 0.67 | 0.61 | 0.018 | 0.58 | 0.65 | 1.10 | 9.5E-04 |
|  |  | 2020-03-11 | 0.76 | 0.60 | 0.018 | 0.57 | 0.64 | 1.27 | 1.1E-15 |
|  |  | 2020-03-18 | 0.65 | 0.62 | 0.019 | 0.58 | 0.65 | 1.04 | 0.1459 |
|  |  | 2020-03-25 | 0.63 | 0.63 | 0.019 | 0.59 | 0.67 | 1.00 | 0.8889 |
|  |  | 2020-04-01 | 0.61 | 0.64 | 0.019 | 0.60 | 0.67 | 0.96 | 0.2456 |
|  |  | 2020-04-08 | 0.47 | 0.51 | 0.019 | 0.48 | 0.55 | 0.92 | 0.0284 |
|  |  | 2020-04-15 | 0.59 | 0.63 | 0.018 | 0.59 | 0.66 | 0.93 | 0.0250 |
|  |  | 2020-04-22 | 0.60 | 0.64 | 0.018 | 0.61 | 0.68 | 0.93 | 0.0215 |
|  |  | 2020-04-29 | 0.55 | 0.60 | 0.018 | 0.57 | 0.64 | 0.91 | 0.0033 |
|  |  | 2020-05-06 | 0.58 | 0.63 | 0.018 | 0.59 | 0.67 | 0.93 | 0.0117 |
|  |  | 2020-05-13 | 0.59 | 0.62 | 0.018 | 0.58 | 0.65 | 0.95 | 0.1050 |
|  |  | 2020-05-20 | 0.55 | 0.59 | 0.018 | 0.55 | 0.62 | 0.93 | 0.0331 |
|  |  | 2020-05-27 | 0.59 | 0.64 | 0.018 | 0.60 | 0.67 | 0.93 | 0.0090 |
|  |  | 2020-06-03 | 0.60 | 0.61 | 0.018 | 0.57 | 0.64 | 0.99 | 0.7628 |
|  |  | 2020-06-10 | 0.61 | 0.66 | 0.018 | 0.62 | 0.69 | 0.92 | 0.0034 |
|  |  | 2020-06-17 | 0.55 | 0.61 | 0.018 | 0.57 | 0.64 | 0.90 | 9.4E-04 |
|  |  | 2020-06-24 | 0.62 | 0.69 | 0.018 | 0.65 | 0.72 | 0.91 | 4.0E-04 |
|  |  | 2020-07-01 | 0.63 | 0.66 | 0.018 | 0.62 | 0.69 | 0.96 | 0.1766 |
|  |  | 2020-07-08 | 0.58 | 0.61 | 0.018 | 0.58 | 0.65 | 0.95 | 0.1188 |
|  |  | 2020-07-15 | 0.57 | 0.59 | 0.018 | 0.56 | 0.63 | 0.96 | 0.2160 |
|  |  | 2020-07-22 | 0.58 | 0.59 | 0.018 | 0.55 | 0.62 | 0.99 | 0.6322 |
|  |  | 2020-07-29 | 0.57 | 0.59 | 0.018 | 0.55 | 0.62 | 0.96 | 0.2538 |
|  |  | 2020-08-05 | 0.57 | 0.59 | 0.018 | 0.56 | 0.63 | 0.96 | 0.1409 |
|  |  | 2020-08-12 | 0.57 | 0.60 | 0.018 | 0.56 | 0.64 | 0.94 | 0.0627 |
|  |  | 2020-08-19 | 0.61 | 0.61 | 0.018 | 0.57 | 0.64 | 1.00 | 0.8790 |
|  |  | 2020-08-26 | 0.60 | 0.62 | 0.018 | 0.59 | 0.66 | 0.96 | 0.1980 |
|  |  | 2020-09-02 | 0.58 | 0.61 | 0.018 | 0.58 | 0.65 | 0.94 | 0.0482 |
|  |  | 2020-09-09 | 0.59 | 0.60 | 0.018 | 0.56 | 0.63 | 0.98 | 0.5689 |
|  |  | 2020-09-16 | 0.58 | 0.60 | 0.018 | 0.56 | 0.64 | 0.96 | 0.2137 |
|  |  | 2020-09-23 | 0.59 | 0.62 | 0.018 | 0.59 | 0.66 | 0.94 | 0.0472 |
|  |  | 2020-09-30 | 0.59 | 0.61 | 0.018 | 0.57 | 0.64 | 0.97 | 0.3826 |
|  |  | 2020-10-07 | 0.57 | 0.61 | 0.018 | 0.57 | 0.64 | 0.94 | 0.0307 |
|  |  | 2020-10-14 | 0.61 | 0.61 | 0.018 | 0.57 | 0.64 | 1.01 | 0.8054 |
|  |  | 2020-10-21 | 0.62 | 0.63 | 0.018 | 0.60 | 0.67 | 0.98 | 0.5290 |
|  |  | 2020-10-28 | 0.60 | 0.61 | 0.018 | 0.58 | 0.65 | 0.98 | 0.4779 |
|  |  | 2020-11-04 | 0.60 | 0.63 | 0.018 | 0.60 | 0.67 | 0.96 | 0.1250 |
|  |  | 2020-11-11 | 0.60 | 0.61 | 0.018 | 0.58 | 0.65 | 0.97 | 0.3561 |
|  |  | 2020-11-18 | 0.59 | 0.63 | 0.018 | 0.59 | 0.66 | 0.94 | 0.0562 |
|  |  | 2020-11-25 | 0.63 | 0.65 | 0.018 | 0.62 | 0.69 | 0.97 | 0.2537 |
|  |  | 2020-12-02 | 0.64 | 0.66 | 0.018 | 0.62 | 0.69 | 0.98 | 0.4680 |
|  |  | 2020-12-09 | 0.64 | 0.67 | 0.018 | 0.64 | 0.71 | 0.95 | 0.0699 |
|  |  | 2020-12-16 | 0.68 | 0.70 | 0.018 | 0.66 | 0.74 | 0.97 | 0.2024 |
|  |  | 2020-12-23 | 0.51 | 0.52 | 0.018 | 0.48 | 0.55 | 0.98 | 0.5968 |
| J05AE | Protease inhibitors | 2020-01-01 | 0.0062 | -0 | 0.0031 | -0 | 0.0061 | . | . |
|  |  | 2020-01-08 | 0.011 | 0.0067 | 0.0031 | 0 | 0.013 | 1.71 | 0.1254 |
|  |  | 2020-01-15 | 0.0079 | 0.0058 | 0.0031 | -0 | 0.012 | 1.36 | 0.4960 |
|  |  | 2020-01-22 | 0.0063 | 0.0057 | 0.0031 | -0 | 0.012 | 1.10 | 0.8469 |
|  |  | 2020-01-29 | 0.0058 | 0.0050 | 0.0031 | -0 | 0.011 | 1.15 | 0.8051 |
|  |  | 2020-02-05 | 0.0066 | 0.0048 | 0.0031 | -0 | 0.011 | 1.38 | 0.5611 |
|  |  | 2020-02-12 | 0.0063 | 0.0043 | 0.0031 | -0 | 0.010 | 1.46 | 0.5236 |
|  |  | 2020-02-19 | 0.0054 | 0.0046 | 0.0031 | -0 | 0.011 | 1.19 | 0.7842 |
|  |  | 2020-02-26 | 0.0098 | 0.0032 | 0.0031 | -0 | 0.0093 | 3.06 | 0.0344 |
|  |  | 2020-03-04 | 0.0077 | 0.0063 | 0.0031 | 0 | 0.012 | 1.22 | 0.6500 |
|  |  | 2020-03-11 | 0.015 | 0.0037 | 0.0032 | -0 | 0.0099 | 4.12 | 3.4E-04 |
|  |  | 2020-03-18 | 0.012 | 0.0028 | 0.0032 | -0 | 0.0091 | 4.34 | 0.0031 |
|  |  | 2020-03-25 | 0.0097 | 0.0045 | 0.0033 | -0 | 0.011 | 2.14 | 0.1238 |
|  |  | 2020-04-01 | 0.0066 | 0.0031 | 0.0033 | -0 | 0.0096 | 2.15 | 0.2894 |
|  |  | 2020-04-08 | 0.0055 | -0 | 0.0033 | -0 | 0.0052 | . | . |
|  |  | 2020-04-15 | 0.0041 | 0.0070 | 0.0031 | 0 | 0.013 | 0.58 | 0.3517 |
|  |  | 2020-04-22 | 0.0049 | 0.0050 | 0.0031 | -0 | 0.011 | 0.98 | 0.9790 |
|  |  | 2020-04-29 | 0.0062 | 0 | 0.0031 | -0 | 0.0069 | 8.49 | 0.0804 |
|  |  | 2020-05-06 | 0.0087 | 0.0047 | 0.0031 | -0 | 0.011 | 1.85 | 0.1996 |
|  |  | 2020-05-13 | 0.0065 | 0.0026 | 0.0031 | -0 | 0.0086 | 2.54 | 0.2059 |
|  |  | 2020-05-20 | 0.0049 | 0.0019 | 0.0031 | -0 | 0.0081 | 2.59 | 0.3329 |
|  |  | 2020-05-27 | 0.0086 | 0.0064 | 0.0031 | 0 | 0.013 | 1.34 | 0.4799 |
|  |  | 2020-06-03 | 0.0072 | 0.0046 | 0.0031 | -0 | 0.011 | 1.57 | 0.4033 |
|  |  | 2020-06-10 | 0.0041 | 0.0052 | 0.0031 | -0 | 0.011 | 0.78 | 0.7190 |
|  |  | 2020-06-17 | 0.0080 | 0.0030 | 0.0031 | -0 | 0.0091 | 2.64 | 0.1094 |
|  |  | 2020-06-24 | 0.0062 | 0.0053 | 0.0031 | -0 | 0.011 | 1.16 | 0.7795 |
|  |  | 2020-07-01 | 0.0065 | 0.0021 | 0.0031 | -0 | 0.0082 | 3.03 | 0.1620 |
|  |  | 2020-07-08 | 0.0048 | 0.0020 | 0.0031 | -0 | 0.0081 | 2.39 | 0.3647 |
|  |  | 2020-07-15 | 0.0059 | 0.0010 | 0.0031 | -0 | 0.0071 | 5.86 | 0.1154 |
|  |  | 2020-07-22 | 0.0050 | 0.0020 | 0.0031 | -0 | 0.0081 | 2.48 | 0.3335 |
|  |  | 2020-07-29 | 0.0073 | -0 | 0.0031 | -0 | 0.0056 | . | . |
|  |  | 2020-08-05 | 0.0061 | 0 | 0.0031 | -0 | 0.0067 | 10.45 | 0.0766 |
|  |  | 2020-08-12 | 0.0058 | 0 | 0.0031 | -0 | 0.0067 | 9.23 | 0.0962 |
|  |  | 2020-08-19 | 0.0069 | 0.0032 | 0.0031 | -0 | 0.0093 | 2.15 | 0.2367 |
|  |  | 2020-08-26 | 0.0055 | 0.0036 | 0.0031 | -0 | 0.0096 | 1.55 | 0.5282 |
|  |  | 2020-09-02 | 0.010 | 0.0026 | 0.0031 | -0 | 0.0087 | 3.82 | 0.0174 |
|  |  | 2020-09-09 | 0.0066 | 0.0037 | 0.0031 | -0 | 0.0098 | 1.79 | 0.3500 |
|  |  | 2020-09-16 | 0.0048 | 0.0028 | 0.0031 | -0 | 0.0089 | 1.71 | 0.5171 |
|  |  | 2020-09-23 | 0.011 | 0.0030 | 0.0031 | -0 | 0.0091 | 3.48 | 0.0162 |
|  |  | 2020-09-30 | 0.0082 | 0.0020 | 0.0031 | -0 | 0.0081 | 4.04 | 0.0474 |
|  |  | 2020-10-07 | 0.0056 | 0.0019 | 0.0031 | -0 | 0.0080 | 3.00 | 0.2288 |
|  |  | 2020-10-14 | 0.0044 | 0 | 0.0031 | -0 | 0.0065 | 12.38 | 0.1886 |
|  |  | 2020-10-21 | 0.0065 | 0 | 0.0031 | -0 | 0.0064 | 18.22 | 0.0496 |
|  |  | 2020-10-28 | 0.0055 | 0.0018 | 0.0031 | -0 | 0.0079 | 3.12 | 0.2285 |
|  |  | 2020-11-04 | 0.0076 | 0.0025 | 0.0031 | -0 | 0.0086 | 3.05 | 0.0997 |
|  |  | 2020-11-11 | 0.0054 | 0 | 0.0031 | -0 | 0.0064 | 20.29 | 0.0985 |
|  |  | 2020-11-18 | 0.0055 | 0 | 0.0031 | -0 | 0.0071 | 5.58 | 0.1462 |
|  |  | 2020-11-25 | 0.0080 | 0.0022 | 0.0031 | -0 | 0.0083 | 3.58 | 0.0636 |
|  |  | 2020-12-02 | 0.0053 | 0.0030 | 0.0031 | -0 | 0.0091 | 1.77 | 0.4582 |
|  |  | 2020-12-09 | 0.0060 | 0.0023 | 0.0031 | -0 | 0.0084 | 2.57 | 0.2388 |
|  |  | 2020-12-16 | 0.0072 | 0.0038 | 0.0031 | -0 | 0.0100 | 1.91 | 0.2723 |
|  |  | 2020-12-23 | 0.0031 | -0 | 0.0031 | -0 | -0 | . | . |
| J05AF | Nucleoside and nucleotide reverse transcriptase inhibitors | 2020-01-01 | 0.018 | 0.020 | 0.0018 | 0.016 | 0.023 | 0.92 | 0.4063 |
|  |  | 2020-01-08 | 0.026 | 0.024 | 0.0018 | 0.021 | 0.028 | 1.08 | 0.2980 |
|  |  | 2020-01-15 | 0.025 | 0.023 | 0.0018 | 0.020 | 0.027 | 1.06 | 0.4216 |
|  |  | 2020-01-22 | 0.024 | 0.022 | 0.0018 | 0.019 | 0.026 | 1.08 | 0.3295 |
|  |  | 2020-01-29 | 0.024 | 0.023 | 0.0018 | 0.019 | 0.026 | 1.04 | 0.6288 |
|  |  | 2020-02-05 | 0.024 | 0.023 | 0.0018 | 0.019 | 0.026 | 1.04 | 0.5797 |
|  |  | 2020-02-12 | 0.022 | 0.023 | 0.0018 | 0.020 | 0.027 | 0.95 | 0.5461 |
|  |  | 2020-02-19 | 0.023 | 0.024 | 0.0018 | 0.021 | 0.028 | 0.93 | 0.3204 |
|  |  | 2020-02-26 | 0.024 | 0.024 | 0.0018 | 0.020 | 0.027 | 1.01 | 0.8742 |
|  |  | 2020-03-04 | 0.028 | 0.024 | 0.0018 | 0.020 | 0.027 | 1.19 | 0.0112 |
|  |  | 2020-03-11 | 0.034 | 0.024 | 0.0018 | 0.021 | 0.028 | 1.40 | 1.4E-07 |
|  |  | 2020-03-18 | 0.034 | 0.025 | 0.0018 | 0.021 | 0.028 | 1.36 | 2.0E-06 |
|  |  | 2020-03-25 | 0.025 | 0.022 | 0.0019 | 0.018 | 0.026 | 1.15 | 0.0854 |
|  |  | 2020-04-01 | 0.026 | 0.025 | 0.0019 | 0.021 | 0.029 | 1.02 | 0.7631 |
|  |  | 2020-04-08 | 0.015 | 0.019 | 0.0019 | 0.015 | 0.022 | 0.79 | 0.0413 |
|  |  | 2020-04-15 | 0.021 | 0.024 | 0.0018 | 0.020 | 0.027 | 0.89 | 0.1465 |
|  |  | 2020-04-22 | 0.021 | 0.025 | 0.0018 | 0.022 | 0.029 | 0.85 | 0.0307 |
|  |  | 2020-04-29 | 0.020 | 0.022 | 0.0018 | 0.019 | 0.026 | 0.90 | 0.1958 |
|  |  | 2020-05-06 | 0.020 | 0.025 | 0.0018 | 0.022 | 0.029 | 0.80 | 0.0045 |
|  |  | 2020-05-13 | 0.024 | 0.024 | 0.0018 | 0.021 | 0.028 | 1.00 | 0.9555 |
|  |  | 2020-05-20 | 0.019 | 0.023 | 0.0018 | 0.020 | 0.027 | 0.83 | 0.0296 |
|  |  | 2020-05-27 | 0.023 | 0.025 | 0.0018 | 0.022 | 0.029 | 0.91 | 0.1884 |
|  |  | 2020-06-03 | 0.024 | 0.025 | 0.0018 | 0.021 | 0.028 | 0.97 | 0.6288 |
|  |  | 2020-06-10 | 0.023 | 0.025 | 0.0018 | 0.021 | 0.028 | 0.93 | 0.2983 |
|  |  | 2020-06-17 | 0.017 | 0.023 | 0.0018 | 0.020 | 0.027 | 0.76 | 0.0015 |
|  |  | 2020-06-24 | 0.024 | 0.026 | 0.0018 | 0.023 | 0.030 | 0.92 | 0.2078 |
|  |  | 2020-07-01 | 0.023 | 0.024 | 0.0018 | 0.020 | 0.027 | 0.99 | 0.8544 |
|  |  | 2020-07-08 | 0.022 | 0.023 | 0.0018 | 0.020 | 0.027 | 0.95 | 0.5394 |
|  |  | 2020-07-15 | 0.022 | 0.020 | 0.0018 | 0.017 | 0.024 | 1.08 | 0.3663 |
|  |  | 2020-07-22 | 0.020 | 0.021 | 0.0018 | 0.017 | 0.024 | 0.96 | 0.6780 |
|  |  | 2020-07-29 | 0.022 | 0.021 | 0.0018 | 0.018 | 0.024 | 1.06 | 0.4544 |
|  |  | 2020-08-05 | 0.022 | 0.022 | 0.0018 | 0.019 | 0.025 | 1.01 | 0.9014 |
|  |  | 2020-08-12 | 0.027 | 0.023 | 0.0018 | 0.020 | 0.026 | 1.15 | 0.0462 |
|  |  | 2020-08-19 | 0.025 | 0.024 | 0.0018 | 0.021 | 0.028 | 1.03 | 0.6435 |
|  |  | 2020-08-26 | 0.021 | 0.026 | 0.0018 | 0.022 | 0.029 | 0.82 | 0.0095 |
|  |  | 2020-09-02 | 0.024 | 0.023 | 0.0018 | 0.019 | 0.026 | 1.05 | 0.5510 |
|  |  | 2020-09-09 | 0.023 | 0.023 | 0.0018 | 0.020 | 0.027 | 0.98 | 0.8072 |
|  |  | 2020-09-16 | 0.023 | 0.023 | 0.0018 | 0.020 | 0.027 | 0.98 | 0.8268 |
|  |  | 2020-09-23 | 0.023 | 0.022 | 0.0018 | 0.019 | 0.026 | 1.06 | 0.4817 |
|  |  | 2020-09-30 | 0.025 | 0.024 | 0.0018 | 0.021 | 0.028 | 1.03 | 0.6484 |
|  |  | 2020-10-07 | 0.021 | 0.024 | 0.0018 | 0.020 | 0.027 | 0.89 | 0.1543 |
|  |  | 2020-10-14 | 0.024 | 0.024 | 0.0018 | 0.020 | 0.027 | 1.01 | 0.9065 |
|  |  | 2020-10-21 | 0.024 | 0.023 | 0.0018 | 0.019 | 0.026 | 1.07 | 0.3497 |
|  |  | 2020-10-28 | 0.024 | 0.024 | 0.0018 | 0.021 | 0.028 | 0.99 | 0.8528 |
|  |  | 2020-11-04 | 0.022 | 0.024 | 0.0018 | 0.020 | 0.027 | 0.93 | 0.3339 |
|  |  | 2020-11-11 | 0.027 | 0.024 | 0.0018 | 0.021 | 0.028 | 1.10 | 0.1508 |
|  |  | 2020-11-18 | 0.025 | 0.025 | 0.0018 | 0.022 | 0.029 | 1.00 | 0.9681 |
|  |  | 2020-11-25 | 0.025 | 0.025 | 0.0018 | 0.022 | 0.029 | 0.99 | 0.8862 |
|  |  | 2020-12-02 | 0.026 | 0.027 | 0.0018 | 0.023 | 0.030 | 0.99 | 0.8542 |
|  |  | 2020-12-09 | 0.026 | 0.026 | 0.0018 | 0.023 | 0.030 | 0.97 | 0.6307 |
|  |  | 2020-12-16 | 0.026 | 0.025 | 0.0018 | 0.022 | 0.029 | 1.03 | 0.7227 |
|  |  | 2020-12-23 | 0.018 | 0.015 | 0.0018 | 0.012 | 0.019 | 1.17 | 0.1437 |
| J05AG | Non-nucleoside reverse transcriptase inhibitors | 2020-01-01 | 0.0042 | 0.0045 | 0.0012 | 0.0021 | 0.0070 | 0.92 | 0.7793 |
|  |  | 2020-01-08 | 0.0094 | 0.0069 | 0.0012 | 0.0045 | 0.0093 | 1.36 | 0.0432 |
|  |  | 2020-01-15 | 0.0058 | 0.0060 | 0.0012 | 0.0036 | 0.0084 | 0.97 | 0.8809 |
|  |  | 2020-01-22 | 0.0080 | 0.0058 | 0.0012 | 0.0034 | 0.0082 | 1.38 | 0.0721 |
|  |  | 2020-01-29 | 0.0062 | 0.0062 | 0.0012 | 0.0038 | 0.0087 | 0.99 | 0.9731 |
|  |  | 2020-02-05 | 0.0087 | 0.0070 | 0.0012 | 0.0046 | 0.0094 | 1.24 | 0.1689 |
|  |  | 2020-02-12 | 0.0068 | 0.0064 | 0.0012 | 0.0040 | 0.0088 | 1.06 | 0.7546 |
|  |  | 2020-02-19 | 0.0056 | 0.0060 | 0.0012 | 0.0036 | 0.0085 | 0.93 | 0.7380 |
|  |  | 2020-02-26 | 0.0069 | 0.0056 | 0.0012 | 0.0032 | 0.0080 | 1.23 | 0.2992 |
|  |  | 2020-03-04 | 0.0075 | 0.0060 | 0.0012 | 0.0036 | 0.0084 | 1.25 | 0.2155 |
|  |  | 2020-03-11 | 0.0085 | 0.0057 | 0.0013 | 0.0033 | 0.0082 | 1.48 | 0.0285 |
|  |  | 2020-03-18 | 0.010 | 0.0076 | 0.0013 | 0.0051 | 0.010 | 1.37 | 0.0265 |
|  |  | 2020-03-25 | 0.0082 | 0.0060 | 0.0013 | 0.0034 | 0.0086 | 1.38 | 0.0918 |
|  |  | 2020-04-01 | 0.0073 | 0.0061 | 0.0013 | 0.0035 | 0.0087 | 1.21 | 0.3424 |
|  |  | 2020-04-08 | 0.0052 | 0.0044 | 0.0013 | 0.0018 | 0.0070 | 1.19 | 0.5196 |
|  |  | 2020-04-15 | 0.0059 | 0.0071 | 0.0012 | 0.0047 | 0.0096 | 0.83 | 0.3187 |
|  |  | 2020-04-22 | 0.0055 | 0.0072 | 0.0012 | 0.0047 | 0.0096 | 0.77 | 0.1775 |
|  |  | 2020-04-29 | 0.0064 | 0.0062 | 0.0012 | 0.0038 | 0.0086 | 1.03 | 0.8868 |
|  |  | 2020-05-06 | 0.0069 | 0.0070 | 0.0012 | 0.0046 | 0.0095 | 0.98 | 0.8975 |
|  |  | 2020-05-13 | 0.0062 | 0.0071 | 0.0012 | 0.0046 | 0.0095 | 0.88 | 0.4834 |
|  |  | 2020-05-20 | 0.0043 | 0.0057 | 0.0012 | 0.0032 | 0.0081 | 0.76 | 0.2801 |
|  |  | 2020-05-27 | 0.0045 | 0.0069 | 0.0012 | 0.0044 | 0.0093 | 0.66 | 0.0628 |
|  |  | 2020-06-03 | 0.0054 | 0.0059 | 0.0012 | 0.0035 | 0.0084 | 0.91 | 0.6633 |
|  |  | 2020-06-10 | 0.0083 | 0.0088 | 0.0012 | 0.0064 | 0.011 | 0.94 | 0.6945 |
|  |  | 2020-06-17 | 0.0059 | 0.0052 | 0.0012 | 0.0028 | 0.0076 | 1.13 | 0.5757 |
|  |  | 2020-06-24 | 0.0064 | 0.0068 | 0.0012 | 0.0044 | 0.0092 | 0.94 | 0.7372 |
|  |  | 2020-07-01 | 0.0054 | 0.0065 | 0.0012 | 0.0041 | 0.0089 | 0.83 | 0.3713 |
|  |  | 2020-07-08 | 0.0045 | 0.0056 | 0.0012 | 0.0032 | 0.0081 | 0.81 | 0.3749 |
|  |  | 2020-07-15 | 0.0073 | 0.0054 | 0.0012 | 0.0030 | 0.0078 | 1.35 | 0.1202 |
|  |  | 2020-07-22 | 0.0076 | 0.0052 | 0.0012 | 0.0028 | 0.0076 | 1.46 | 0.0513 |
|  |  | 2020-07-29 | 0.0053 | 0.0056 | 0.0012 | 0.0032 | 0.0081 | 0.94 | 0.7873 |
|  |  | 2020-08-05 | 0.0059 | 0.0058 | 0.0012 | 0.0033 | 0.0082 | 1.02 | 0.9158 |
|  |  | 2020-08-12 | 0.0060 | 0.0071 | 0.0012 | 0.0047 | 0.0095 | 0.84 | 0.3712 |
|  |  | 2020-08-19 | 0.0083 | 0.0053 | 0.0012 | 0.0028 | 0.0077 | 1.58 | 0.0146 |
|  |  | 2020-08-26 | 0.0052 | 0.0067 | 0.0012 | 0.0043 | 0.0091 | 0.78 | 0.2341 |
|  |  | 2020-09-02 | 0.0062 | 0.0063 | 0.0012 | 0.0038 | 0.0087 | 0.99 | 0.9409 |
|  |  | 2020-09-09 | 0.0050 | 0.0064 | 0.0012 | 0.0040 | 0.0088 | 0.78 | 0.2653 |
|  |  | 2020-09-16 | 0.0045 | 0.0066 | 0.0012 | 0.0041 | 0.0090 | 0.69 | 0.1024 |
|  |  | 2020-09-23 | 0.0071 | 0.0059 | 0.0012 | 0.0035 | 0.0083 | 1.21 | 0.3110 |
|  |  | 2020-09-30 | 0.0072 | 0.0061 | 0.0012 | 0.0037 | 0.0086 | 1.18 | 0.3732 |
|  |  | 2020-10-07 | 0.0065 | 0.0064 | 0.0012 | 0.0040 | 0.0088 | 1.01 | 0.9557 |
|  |  | 2020-10-14 | 0.0055 | 0.0056 | 0.0012 | 0.0032 | 0.0080 | 0.98 | 0.9345 |
|  |  | 2020-10-21 | 0.0076 | 0.0058 | 0.0012 | 0.0034 | 0.0083 | 1.30 | 0.1513 |
|  |  | 2020-10-28 | 0.0067 | 0.0072 | 0.0012 | 0.0048 | 0.0096 | 0.93 | 0.6625 |
|  |  | 2020-11-04 | 0.0060 | 0.0073 | 0.0012 | 0.0049 | 0.0097 | 0.82 | 0.2805 |
|  |  | 2020-11-11 | 0.0064 | 0.0064 | 0.0012 | 0.0040 | 0.0088 | 0.99 | 0.9658 |
|  |  | 2020-11-18 | 0.0059 | 0.0054 | 0.0012 | 0.0030 | 0.0079 | 1.08 | 0.7273 |
|  |  | 2020-11-25 | 0.0064 | 0.0068 | 0.0012 | 0.0044 | 0.0092 | 0.93 | 0.7115 |
|  |  | 2020-12-02 | 0.0065 | 0.0073 | 0.0012 | 0.0049 | 0.0097 | 0.88 | 0.4864 |
|  |  | 2020-12-09 | 0.0072 | 0.0064 | 0.0012 | 0.0040 | 0.0088 | 1.13 | 0.5133 |
|  |  | 2020-12-16 | 0.0077 | 0.0069 | 0.0012 | 0.0045 | 0.0094 | 1.11 | 0.5357 |
|  |  | 2020-12-23 | 0.0035 | 0.0024 | 0.0012 | -0 | 0.0049 | 1.44 | 0.3952 |
| J05AJ | Integrase inhibitors | 2020-01-01 | 0.010 | 0.013 | 0.0018 | 0.0096 | 0.017 | 0.78 | 0.1151 |
|  |  | 2020-01-08 | 0.017 | 0.016 | 0.0018 | 0.012 | 0.020 | 1.05 | 0.6404 |
|  |  | 2020-01-15 | 0.016 | 0.016 | 0.0018 | 0.012 | 0.020 | 1.02 | 0.8478 |
|  |  | 2020-01-22 | 0.017 | 0.016 | 0.0018 | 0.013 | 0.020 | 1.06 | 0.5710 |
|  |  | 2020-01-29 | 0.015 | 0.016 | 0.0018 | 0.012 | 0.019 | 0.93 | 0.5752 |
|  |  | 2020-02-05 | 0.017 | 0.016 | 0.0018 | 0.012 | 0.019 | 1.05 | 0.6845 |
|  |  | 2020-02-12 | 0.015 | 0.016 | 0.0018 | 0.012 | 0.020 | 0.91 | 0.4588 |
|  |  | 2020-02-19 | 0.017 | 0.016 | 0.0018 | 0.013 | 0.020 | 1.03 | 0.7748 |
|  |  | 2020-02-26 | 0.020 | 0.015 | 0.0018 | 0.012 | 0.019 | 1.29 | 0.0167 |
|  |  | 2020-03-04 | 0.018 | 0.016 | 0.0018 | 0.012 | 0.019 | 1.17 | 0.1387 |
|  |  | 2020-03-11 | 0.024 | 0.017 | 0.0019 | 0.013 | 0.020 | 1.46 | 5.8E-05 |
|  |  | 2020-03-18 | 0.025 | 0.016 | 0.0019 | 0.012 | 0.020 | 1.53 | 1.1E-05 |
|  |  | 2020-03-25 | 0.017 | 0.016 | 0.0020 | 0.012 | 0.020 | 1.06 | 0.6349 |
|  |  | 2020-04-01 | 0.017 | 0.017 | 0.0020 | 0.014 | 0.021 | 0.97 | 0.7626 |
|  |  | 2020-04-08 | 0.012 | 0.014 | 0.0020 | 0.0097 | 0.017 | 0.85 | 0.3093 |
|  |  | 2020-04-15 | 0.015 | 0.016 | 0.0018 | 0.013 | 0.020 | 0.91 | 0.4029 |
|  |  | 2020-04-22 | 0.013 | 0.017 | 0.0018 | 0.013 | 0.021 | 0.76 | 0.0265 |
|  |  | 2020-04-29 | 0.012 | 0.015 | 0.0018 | 0.012 | 0.019 | 0.78 | 0.0622 |
|  |  | 2020-05-06 | 0.017 | 0.016 | 0.0018 | 0.013 | 0.020 | 1.06 | 0.5886 |
|  |  | 2020-05-13 | 0.016 | 0.017 | 0.0018 | 0.014 | 0.021 | 0.91 | 0.3964 |
|  |  | 2020-05-20 | 0.014 | 0.017 | 0.0018 | 0.013 | 0.021 | 0.85 | 0.1561 |
|  |  | 2020-05-27 | 0.016 | 0.018 | 0.0018 | 0.014 | 0.021 | 0.91 | 0.4048 |
|  |  | 2020-06-03 | 0.017 | 0.017 | 0.0018 | 0.013 | 0.020 | 1.03 | 0.8148 |
|  |  | 2020-06-10 | 0.017 | 0.018 | 0.0018 | 0.014 | 0.022 | 0.95 | 0.6458 |
|  |  | 2020-06-17 | 0.015 | 0.016 | 0.0018 | 0.012 | 0.019 | 0.98 | 0.8564 |
|  |  | 2020-06-24 | 0.016 | 0.017 | 0.0018 | 0.014 | 0.021 | 0.93 | 0.4985 |
|  |  | 2020-07-01 | 0.016 | 0.016 | 0.0018 | 0.013 | 0.020 | 0.99 | 0.8966 |
|  |  | 2020-07-08 | 0.015 | 0.015 | 0.0018 | 0.012 | 0.019 | 1.00 | 0.9699 |
|  |  | 2020-07-15 | 0.012 | 0.015 | 0.0018 | 0.012 | 0.019 | 0.79 | 0.0722 |
|  |  | 2020-07-22 | 0.017 | 0.016 | 0.0018 | 0.012 | 0.020 | 1.03 | 0.7785 |
|  |  | 2020-07-29 | 0.013 | 0.015 | 0.0018 | 0.012 | 0.019 | 0.84 | 0.1703 |
|  |  | 2020-08-05 | 0.016 | 0.016 | 0.0018 | 0.012 | 0.019 | 1.04 | 0.7341 |
|  |  | 2020-08-12 | 0.016 | 0.016 | 0.0018 | 0.013 | 0.020 | 0.99 | 0.9176 |
|  |  | 2020-08-19 | 0.019 | 0.017 | 0.0018 | 0.013 | 0.020 | 1.15 | 0.1743 |
|  |  | 2020-08-26 | 0.020 | 0.018 | 0.0018 | 0.014 | 0.021 | 1.11 | 0.2726 |
|  |  | 2020-09-02 | 0.019 | 0.018 | 0.0018 | 0.014 | 0.021 | 1.06 | 0.5560 |
|  |  | 2020-09-09 | 0.018 | 0.018 | 0.0018 | 0.014 | 0.022 | 0.98 | 0.8420 |
|  |  | 2020-09-16 | 0.014 | 0.017 | 0.0018 | 0.013 | 0.021 | 0.83 | 0.1099 |
|  |  | 2020-09-23 | 0.017 | 0.018 | 0.0018 | 0.014 | 0.021 | 0.97 | 0.7786 |
|  |  | 2020-09-30 | 0.017 | 0.017 | 0.0018 | 0.013 | 0.021 | 0.99 | 0.9046 |
|  |  | 2020-10-07 | 0.017 | 0.017 | 0.0018 | 0.013 | 0.020 | 1.05 | 0.6540 |
|  |  | 2020-10-14 | 0.015 | 0.017 | 0.0018 | 0.014 | 0.021 | 0.89 | 0.3094 |
|  |  | 2020-10-21 | 0.017 | 0.016 | 0.0018 | 0.013 | 0.020 | 1.02 | 0.8686 |
|  |  | 2020-10-28 | 0.017 | 0.017 | 0.0018 | 0.014 | 0.021 | 0.97 | 0.7683 |
|  |  | 2020-11-04 | 0.019 | 0.017 | 0.0018 | 0.014 | 0.021 | 1.08 | 0.4643 |
|  |  | 2020-11-11 | 0.015 | 0.018 | 0.0018 | 0.015 | 0.022 | 0.81 | 0.0666 |
|  |  | 2020-11-18 | 0.018 | 0.018 | 0.0018 | 0.015 | 0.022 | 1.00 | 0.9891 |
|  |  | 2020-11-25 | 0.019 | 0.018 | 0.0018 | 0.015 | 0.022 | 1.01 | 0.9412 |
|  |  | 2020-12-02 | 0.016 | 0.018 | 0.0018 | 0.015 | 0.022 | 0.90 | 0.3056 |
|  |  | 2020-12-09 | 0.019 | 0.020 | 0.0018 | 0.016 | 0.023 | 0.97 | 0.7622 |
|  |  | 2020-12-16 | 0.016 | 0.018 | 0.0019 | 0.014 | 0.021 | 0.91 | 0.3753 |
|  |  | 2020-12-23 | 0.013 | 0.013 | 0.0018 | 0.0092 | 0.016 | 0.99 | 0.9675 |
| J05AR | Antivirals for treatment of HIV infections, combinations | 2020-01-01 | 0.042 | 0.049 | 0.0040 | 0.041 | 0.057 | 0.84 | 0.0585 |
|  |  | 2020-01-08 | 0.075 | 0.066 | 0.0040 | 0.058 | 0.074 | 1.14 | 0.0258 |
|  |  | 2020-01-15 | 0.068 | 0.060 | 0.0040 | 0.052 | 0.068 | 1.13 | 0.0502 |
|  |  | 2020-01-22 | 0.068 | 0.062 | 0.0040 | 0.054 | 0.070 | 1.09 | 0.1497 |
|  |  | 2020-01-29 | 0.061 | 0.061 | 0.0040 | 0.053 | 0.068 | 1.00 | 0.9616 |
|  |  | 2020-02-05 | 0.067 | 0.060 | 0.0040 | 0.052 | 0.068 | 1.12 | 0.0823 |
|  |  | 2020-02-12 | 0.063 | 0.061 | 0.0040 | 0.053 | 0.069 | 1.05 | 0.4981 |
|  |  | 2020-02-19 | 0.068 | 0.061 | 0.0040 | 0.053 | 0.069 | 1.11 | 0.0939 |
|  |  | 2020-02-26 | 0.074 | 0.059 | 0.0040 | 0.051 | 0.067 | 1.26 | 2.2E-04 |
|  |  | 2020-03-04 | 0.078 | 0.061 | 0.0040 | 0.053 | 0.069 | 1.29 | 2.1E-05 |
|  |  | 2020-03-11 | 0.096 | 0.061 | 0.0041 | 0.053 | 0.070 | 1.57 | 4.1E-15 |
|  |  | 2020-03-18 | 0.097 | 0.061 | 0.0042 | 0.053 | 0.069 | 1.58 | 2.0E-15 |
|  |  | 2020-03-25 | 0.069 | 0.064 | 0.0043 | 0.055 | 0.072 | 1.08 | 0.2542 |
|  |  | 2020-04-01 | 0.066 | 0.063 | 0.0043 | 0.055 | 0.072 | 1.04 | 0.5093 |
|  |  | 2020-04-08 | 0.050 | 0.047 | 0.0043 | 0.039 | 0.056 | 1.06 | 0.5298 |
|  |  | 2020-04-15 | 0.057 | 0.065 | 0.0040 | 0.057 | 0.073 | 0.88 | 0.0512 |
|  |  | 2020-04-22 | 0.055 | 0.064 | 0.0040 | 0.056 | 0.072 | 0.87 | 0.0402 |
|  |  | 2020-04-29 | 0.052 | 0.056 | 0.0040 | 0.048 | 0.064 | 0.92 | 0.2866 |
|  |  | 2020-05-06 | 0.064 | 0.063 | 0.0040 | 0.055 | 0.071 | 1.02 | 0.7667 |
|  |  | 2020-05-13 | 0.062 | 0.061 | 0.0040 | 0.053 | 0.069 | 1.01 | 0.9265 |
|  |  | 2020-05-20 | 0.055 | 0.060 | 0.0041 | 0.052 | 0.068 | 0.91 | 0.2103 |
|  |  | 2020-05-27 | 0.062 | 0.066 | 0.0040 | 0.059 | 0.074 | 0.94 | 0.2946 |
|  |  | 2020-06-03 | 0.066 | 0.063 | 0.0040 | 0.055 | 0.071 | 1.05 | 0.4446 |
|  |  | 2020-06-10 | 0.065 | 0.072 | 0.0040 | 0.064 | 0.080 | 0.90 | 0.0732 |
|  |  | 2020-06-17 | 0.056 | 0.058 | 0.0040 | 0.050 | 0.066 | 0.96 | 0.5664 |
|  |  | 2020-06-24 | 0.066 | 0.066 | 0.0040 | 0.058 | 0.074 | 1.01 | 0.8587 |
|  |  | 2020-07-01 | 0.065 | 0.061 | 0.0040 | 0.053 | 0.068 | 1.07 | 0.2794 |
|  |  | 2020-07-08 | 0.063 | 0.058 | 0.0040 | 0.050 | 0.066 | 1.09 | 0.2007 |
|  |  | 2020-07-15 | 0.057 | 0.057 | 0.0040 | 0.049 | 0.065 | 1.00 | 0.9961 |
|  |  | 2020-07-22 | 0.063 | 0.057 | 0.0040 | 0.049 | 0.065 | 1.10 | 0.1497 |
|  |  | 2020-07-29 | 0.056 | 0.056 | 0.0040 | 0.048 | 0.064 | 0.99 | 0.9206 |
|  |  | 2020-08-05 | 0.063 | 0.058 | 0.0040 | 0.050 | 0.066 | 1.08 | 0.2437 |
|  |  | 2020-08-12 | 0.061 | 0.062 | 0.0040 | 0.054 | 0.070 | 0.98 | 0.7443 |
|  |  | 2020-08-19 | 0.070 | 0.063 | 0.0040 | 0.055 | 0.071 | 1.11 | 0.0766 |
|  |  | 2020-08-26 | 0.065 | 0.065 | 0.0040 | 0.058 | 0.073 | 0.99 | 0.8787 |
|  |  | 2020-09-02 | 0.071 | 0.063 | 0.0040 | 0.055 | 0.071 | 1.12 | 0.0624 |
|  |  | 2020-09-09 | 0.068 | 0.066 | 0.0040 | 0.058 | 0.074 | 1.03 | 0.6305 |
|  |  | 2020-09-16 | 0.062 | 0.064 | 0.0040 | 0.056 | 0.072 | 0.98 | 0.7077 |
|  |  | 2020-09-23 | 0.071 | 0.063 | 0.0040 | 0.055 | 0.071 | 1.12 | 0.0614 |
|  |  | 2020-09-30 | 0.069 | 0.065 | 0.0040 | 0.057 | 0.073 | 1.05 | 0.3925 |
|  |  | 2020-10-07 | 0.070 | 0.065 | 0.0040 | 0.057 | 0.073 | 1.08 | 0.1920 |
|  |  | 2020-10-14 | 0.065 | 0.063 | 0.0040 | 0.055 | 0.070 | 1.03 | 0.5931 |
|  |  | 2020-10-21 | 0.070 | 0.064 | 0.0040 | 0.056 | 0.072 | 1.09 | 0.1771 |
|  |  | 2020-10-28 | 0.064 | 0.064 | 0.0040 | 0.056 | 0.072 | 1.01 | 0.8624 |
|  |  | 2020-11-04 | 0.070 | 0.066 | 0.0040 | 0.058 | 0.074 | 1.07 | 0.2794 |
|  |  | 2020-11-11 | 0.067 | 0.065 | 0.0040 | 0.057 | 0.073 | 1.04 | 0.5351 |
|  |  | 2020-11-18 | 0.070 | 0.064 | 0.0040 | 0.056 | 0.072 | 1.08 | 0.1817 |
|  |  | 2020-11-25 | 0.072 | 0.067 | 0.0040 | 0.059 | 0.075 | 1.07 | 0.2727 |
|  |  | 2020-12-02 | 0.073 | 0.070 | 0.0040 | 0.062 | 0.078 | 1.04 | 0.5192 |
|  |  | 2020-12-09 | 0.076 | 0.070 | 0.0040 | 0.062 | 0.078 | 1.08 | 0.1495 |
|  |  | 2020-12-16 | 0.073 | 0.068 | 0.0041 | 0.060 | 0.076 | 1.07 | 0.2679 |
|  |  | 2020-12-23 | 0.049 | 0.041 | 0.0040 | 0.033 | 0.049 | 1.19 | 0.0588 |
| P01 | Antiprotozoals | 2020-01-01 | 0.29 | 0.30 | 0.018 | 0.27 | 0.34 | 0.97 | 0.6260 |
|  |  | 2020-01-08 | 0.42 | 0.40 | 0.017 | 0.36 | 0.43 | 1.05 | 0.2966 |
|  |  | 2020-01-15 | 0.42 | 0.40 | 0.017 | 0.37 | 0.44 | 1.04 | 0.4103 |
|  |  | 2020-01-22 | 0.42 | 0.40 | 0.017 | 0.37 | 0.44 | 1.04 | 0.3325 |
|  |  | 2020-01-29 | 0.39 | 0.39 | 0.017 | 0.36 | 0.43 | 0.98 | 0.7015 |
|  |  | 2020-02-05 | 0.37 | 0.38 | 0.017 | 0.34 | 0.41 | 0.98 | 0.6852 |
|  |  | 2020-02-12 | 0.36 | 0.36 | 0.017 | 0.33 | 0.40 | 0.99 | 0.7909 |
|  |  | 2020-02-19 | 0.34 | 0.35 | 0.017 | 0.32 | 0.39 | 0.97 | 0.5554 |
|  |  | 2020-02-26 | 0.37 | 0.35 | 0.017 | 0.31 | 0.38 | 1.05 | 0.3140 |
|  |  | 2020-03-04 | 0.34 | 0.34 | 0.017 | 0.30 | 0.37 | 1.02 | 0.6467 |
|  |  | 2020-03-11 | 0.36 | 0.33 | 0.018 | 0.30 | 0.37 | 1.09 | 0.0969 |
|  |  | 2020-03-18 | 0.38 | 0.33 | 0.018 | 0.29 | 0.37 | 1.15 | 0.0065 |
|  |  | 2020-03-25 | 0.34 | 0.33 | 0.019 | 0.29 | 0.37 | 1.03 | 0.5422 |
|  |  | 2020-04-01 | 0.29 | 0.32 | 0.019 | 0.28 | 0.36 | 0.90 | 0.0940 |
|  |  | 2020-04-08 | 0.18 | 0.22 | 0.019 | 0.19 | 0.26 | 0.79 | 0.0127 |
|  |  | 2020-04-15 | 0.24 | 0.31 | 0.018 | 0.27 | 0.34 | 0.78 | 1.4E-04 |
|  |  | 2020-04-22 | 0.23 | 0.32 | 0.017 | 0.28 | 0.35 | 0.74 | 3.8E-06 |
|  |  | 2020-04-29 | 0.21 | 0.27 | 0.018 | 0.24 | 0.31 | 0.77 | 4.7E-04 |
|  |  | 2020-05-06 | 0.24 | 0.31 | 0.017 | 0.28 | 0.34 | 0.77 | 5.2E-05 |
|  |  | 2020-05-13 | 0.24 | 0.31 | 0.017 | 0.27 | 0.34 | 0.79 | 2.6E-04 |
|  |  | 2020-05-20 | 0.21 | 0.28 | 0.018 | 0.25 | 0.32 | 0.75 | 6.9E-05 |
|  |  | 2020-05-27 | 0.23 | 0.34 | 0.017 | 0.31 | 0.38 | 0.68 | 1.4E-09 |
|  |  | 2020-06-03 | 0.23 | 0.32 | 0.017 | 0.29 | 0.35 | 0.72 | 9.2E-07 |
|  |  | 2020-06-10 | 0.25 | 0.36 | 0.017 | 0.33 | 0.40 | 0.68 | 3.4E-10 |
|  |  | 2020-06-17 | 0.21 | 0.30 | 0.017 | 0.27 | 0.33 | 0.69 | 2.4E-07 |
|  |  | 2020-06-24 | 0.23 | 0.35 | 0.017 | 0.32 | 0.38 | 0.66 | 1.6E-10 |
|  |  | 2020-07-01 | 0.24 | 0.31 | 0.017 | 0.28 | 0.35 | 0.76 | 1.8E-05 |
|  |  | 2020-07-08 | 0.23 | 0.29 | 0.017 | 0.25 | 0.32 | 0.80 | 0.0011 |
|  |  | 2020-07-15 | 0.21 | 0.26 | 0.017 | 0.23 | 0.30 | 0.81 | 0.0051 |
|  |  | 2020-07-22 | 0.22 | 0.26 | 0.017 | 0.23 | 0.30 | 0.84 | 0.0142 |
|  |  | 2020-07-29 | 0.22 | 0.26 | 0.017 | 0.23 | 0.30 | 0.83 | 0.0125 |
|  |  | 2020-08-05 | 0.23 | 0.27 | 0.017 | 0.23 | 0.30 | 0.85 | 0.0222 |
|  |  | 2020-08-12 | 0.22 | 0.29 | 0.017 | 0.26 | 0.32 | 0.76 | 8.2E-05 |
|  |  | 2020-08-19 | 0.26 | 0.31 | 0.017 | 0.28 | 0.35 | 0.82 | 0.0020 |
|  |  | 2020-08-26 | 0.26 | 0.34 | 0.017 | 0.30 | 0.37 | 0.78 | 2.6E-05 |
|  |  | 2020-09-02 | 0.25 | 0.33 | 0.017 | 0.30 | 0.37 | 0.77 | 1.5E-05 |
|  |  | 2020-09-09 | 0.25 | 0.33 | 0.017 | 0.30 | 0.37 | 0.75 | 2.9E-06 |
|  |  | 2020-09-16 | 0.26 | 0.35 | 0.017 | 0.31 | 0.38 | 0.75 | 9.9E-07 |
|  |  | 2020-09-23 | 0.27 | 0.37 | 0.017 | 0.33 | 0.40 | 0.73 | 5.6E-08 |
|  |  | 2020-09-30 | 0.26 | 0.39 | 0.017 | 0.35 | 0.42 | 0.66 | 2.6E-12 |
|  |  | 2020-10-07 | 0.26 | 0.39 | 0.017 | 0.35 | 0.42 | 0.67 | 3.7E-12 |
|  |  | 2020-10-14 | 0.25 | 0.41 | 0.017 | 0.37 | 0.44 | 0.62 | 3.6E-16 |
|  |  | 2020-10-21 | 0.26 | 0.41 | 0.017 | 0.37 | 0.44 | 0.64 | 7.2E-15 |
|  |  | 2020-10-28 | 0.26 | 0.37 | 0.017 | 0.34 | 0.40 | 0.70 | 2.0E-09 |
|  |  | 2020-11-04 | 0.26 | 0.38 | 0.017 | 0.35 | 0.42 | 0.68 | 2.9E-11 |
|  |  | 2020-11-11 | 0.27 | 0.38 | 0.017 | 0.34 | 0.41 | 0.71 | 1.6E-09 |
|  |  | 2020-11-18 | 0.26 | 0.39 | 0.017 | 0.35 | 0.42 | 0.67 | 5.2E-12 |
|  |  | 2020-11-25 | 0.27 | 0.43 | 0.017 | 0.39 | 0.46 | 0.63 | 9.8E-17 |
|  |  | 2020-12-02 | 0.28 | 0.47 | 0.017 | 0.44 | 0.51 | 0.60 | 6.1E-22 |
|  |  | 2020-12-09 | 0.28 | 0.50 | 0.017 | 0.47 | 0.54 | 0.56 | 1.1E-27 |
|  |  | 2020-12-16 | 0.29 | 0.49 | 0.018 | 0.46 | 0.53 | 0.59 | 4.6E-23 |
|  |  | 2020-12-23 | 0.18 | 0.28 | 0.018 | 0.25 | 0.32 | 0.65 | 1.0E-07 |
| P01AB | Nitroimidazole derivatives | 2020-01-01 | 0.12 | 0.11 | 0.0098 | 0.086 | 0.12 | 1.13 | 0.1786 |
|  |  | 2020-01-08 | 0.17 | 0.16 | 0.0098 | 0.14 | 0.18 | 1.07 | 0.2305 |
|  |  | 2020-01-15 | 0.17 | 0.17 | 0.0098 | 0.15 | 0.18 | 1.03 | 0.6709 |
|  |  | 2020-01-22 | 0.18 | 0.17 | 0.0098 | 0.15 | 0.19 | 1.09 | 0.1351 |
|  |  | 2020-01-29 | 0.16 | 0.17 | 0.0098 | 0.15 | 0.19 | 0.98 | 0.7740 |
|  |  | 2020-02-05 | 0.17 | 0.17 | 0.0098 | 0.15 | 0.19 | 1.03 | 0.5792 |
|  |  | 2020-02-12 | 0.17 | 0.16 | 0.0098 | 0.14 | 0.18 | 1.04 | 0.5544 |
|  |  | 2020-02-19 | 0.16 | 0.16 | 0.0098 | 0.14 | 0.18 | 1.01 | 0.8422 |
|  |  | 2020-02-26 | 0.17 | 0.16 | 0.0098 | 0.14 | 0.18 | 1.09 | 0.1502 |
|  |  | 2020-03-04 | 0.17 | 0.16 | 0.0098 | 0.14 | 0.18 | 1.04 | 0.5348 |
|  |  | 2020-03-11 | 0.17 | 0.16 | 0.0100 | 0.14 | 0.18 | 1.06 | 0.3602 |
|  |  | 2020-03-18 | 0.15 | 0.17 | 0.010 | 0.15 | 0.19 | 0.91 | 0.1568 |
|  |  | 2020-03-25 | 0.15 | 0.16 | 0.011 | 0.14 | 0.18 | 0.94 | 0.3747 |
|  |  | 2020-04-01 | 0.15 | 0.16 | 0.010 | 0.14 | 0.18 | 0.98 | 0.8169 |
|  |  | 2020-04-08 | 0.11 | 0.12 | 0.011 | 0.098 | 0.14 | 0.94 | 0.5072 |
|  |  | 2020-04-15 | 0.14 | 0.16 | 0.0098 | 0.14 | 0.18 | 0.89 | 0.0591 |
|  |  | 2020-04-22 | 0.14 | 0.16 | 0.0098 | 0.14 | 0.18 | 0.88 | 0.0468 |
|  |  | 2020-04-29 | 0.14 | 0.15 | 0.0098 | 0.13 | 0.17 | 0.93 | 0.2866 |
|  |  | 2020-05-06 | 0.15 | 0.16 | 0.0098 | 0.14 | 0.18 | 0.94 | 0.3544 |
|  |  | 2020-05-13 | 0.16 | 0.16 | 0.0098 | 0.14 | 0.18 | 1.00 | 0.9810 |
|  |  | 2020-05-20 | 0.13 | 0.14 | 0.0099 | 0.12 | 0.16 | 0.94 | 0.3641 |
|  |  | 2020-05-27 | 0.15 | 0.16 | 0.0098 | 0.14 | 0.18 | 0.91 | 0.1433 |
|  |  | 2020-06-03 | 0.15 | 0.14 | 0.0098 | 0.12 | 0.16 | 1.02 | 0.7977 |
|  |  | 2020-06-10 | 0.15 | 0.16 | 0.0098 | 0.14 | 0.18 | 0.94 | 0.3336 |
|  |  | 2020-06-17 | 0.13 | 0.14 | 0.0098 | 0.12 | 0.16 | 0.93 | 0.3312 |
|  |  | 2020-06-24 | 0.14 | 0.16 | 0.0098 | 0.14 | 0.18 | 0.84 | 0.0080 |
|  |  | 2020-07-01 | 0.14 | 0.15 | 0.0098 | 0.13 | 0.17 | 0.97 | 0.6257 |
|  |  | 2020-07-08 | 0.15 | 0.14 | 0.0098 | 0.12 | 0.16 | 1.05 | 0.5115 |
|  |  | 2020-07-15 | 0.13 | 0.13 | 0.0098 | 0.11 | 0.15 | 1.04 | 0.6469 |
|  |  | 2020-07-22 | 0.13 | 0.13 | 0.0098 | 0.11 | 0.15 | 1.07 | 0.3492 |
|  |  | 2020-07-29 | 0.13 | 0.12 | 0.0098 | 0.11 | 0.14 | 1.06 | 0.4174 |
|  |  | 2020-08-05 | 0.14 | 0.13 | 0.0098 | 0.11 | 0.15 | 1.04 | 0.5762 |
|  |  | 2020-08-12 | 0.13 | 0.14 | 0.0098 | 0.12 | 0.16 | 0.92 | 0.2297 |
|  |  | 2020-08-19 | 0.16 | 0.15 | 0.0098 | 0.13 | 0.17 | 1.01 | 0.8436 |
|  |  | 2020-08-26 | 0.16 | 0.16 | 0.0098 | 0.14 | 0.18 | 1.00 | 0.9993 |
|  |  | 2020-09-02 | 0.16 | 0.16 | 0.0098 | 0.14 | 0.18 | 0.99 | 0.9179 |
|  |  | 2020-09-09 | 0.16 | 0.16 | 0.0098 | 0.14 | 0.18 | 0.98 | 0.8060 |
|  |  | 2020-09-16 | 0.16 | 0.16 | 0.0098 | 0.14 | 0.18 | 1.01 | 0.8625 |
|  |  | 2020-09-23 | 0.17 | 0.16 | 0.0098 | 0.14 | 0.18 | 1.04 | 0.4842 |
|  |  | 2020-09-30 | 0.16 | 0.16 | 0.0098 | 0.15 | 0.18 | 0.96 | 0.5100 |
|  |  | 2020-10-07 | 0.16 | 0.16 | 0.0098 | 0.14 | 0.18 | 0.98 | 0.7313 |
|  |  | 2020-10-14 | 0.15 | 0.16 | 0.0098 | 0.15 | 0.18 | 0.94 | 0.3089 |
|  |  | 2020-10-21 | 0.16 | 0.16 | 0.0098 | 0.14 | 0.18 | 1.01 | 0.9019 |
|  |  | 2020-10-28 | 0.16 | 0.15 | 0.0098 | 0.13 | 0.17 | 1.05 | 0.4245 |
|  |  | 2020-11-04 | 0.16 | 0.16 | 0.0098 | 0.15 | 0.18 | 0.97 | 0.5615 |
|  |  | 2020-11-11 | 0.16 | 0.16 | 0.0098 | 0.14 | 0.18 | 1.02 | 0.7587 |
|  |  | 2020-11-18 | 0.16 | 0.16 | 0.0098 | 0.14 | 0.18 | 1.00 | 0.9915 |
|  |  | 2020-11-25 | 0.16 | 0.16 | 0.0098 | 0.14 | 0.18 | 0.98 | 0.7626 |
|  |  | 2020-12-02 | 0.17 | 0.16 | 0.0098 | 0.14 | 0.18 | 1.01 | 0.9099 |
|  |  | 2020-12-09 | 0.16 | 0.17 | 0.0098 | 0.15 | 0.19 | 0.96 | 0.5191 |
|  |  | 2020-12-16 | 0.17 | 0.17 | 0.0099 | 0.15 | 0.19 | 1.00 | 0.9554 |
|  |  | 2020-12-23 | 0.11 | 0.097 | 0.0098 | 0.077 | 0.12 | 1.17 | 0.1054 |
| P01BA | Aminoquinolines | 2020-01-01 | 0.053 | 0.058 | 0.0041 | 0.049 | 0.066 | 0.91 | 0.2231 |
|  |  | 2020-01-08 | 0.074 | 0.072 | 0.0041 | 0.064 | 0.080 | 1.03 | 0.6017 |
|  |  | 2020-01-15 | 0.076 | 0.075 | 0.0041 | 0.067 | 0.083 | 1.01 | 0.8199 |
|  |  | 2020-01-22 | 0.074 | 0.074 | 0.0041 | 0.066 | 0.083 | 1.00 | 0.9848 |
|  |  | 2020-01-29 | 0.072 | 0.072 | 0.0041 | 0.064 | 0.080 | 1.00 | 0.9409 |
|  |  | 2020-02-05 | 0.071 | 0.068 | 0.0041 | 0.060 | 0.076 | 1.04 | 0.4779 |
|  |  | 2020-02-12 | 0.066 | 0.070 | 0.0041 | 0.062 | 0.078 | 0.94 | 0.2726 |
|  |  | 2020-02-19 | 0.071 | 0.071 | 0.0041 | 0.063 | 0.079 | 0.99 | 0.8753 |
|  |  | 2020-02-26 | 0.087 | 0.073 | 0.0041 | 0.065 | 0.081 | 1.18 | 0.0014 |
|  |  | 2020-03-04 | 0.097 | 0.070 | 0.0041 | 0.062 | 0.078 | 1.38 | 4.7E-10 |
|  |  | 2020-03-11 | 0.14 | 0.071 | 0.0042 | 0.063 | 0.079 | 2.03 | 9.0E-42 |
|  |  | 2020-03-18 | 0.20 | 0.071 | 0.0042 | 0.063 | 0.080 | 2.78 | 1.1E-75 |
|  |  | 2020-03-25 | 0.16 | 0.072 | 0.0044 | 0.063 | 0.081 | 2.28 | 5.7E-52 |
|  |  | 2020-04-01 | 0.11 | 0.078 | 0.0044 | 0.069 | 0.086 | 1.47 | 1.3E-14 |
|  |  | 2020-04-08 | 0.050 | 0.054 | 0.0044 | 0.045 | 0.062 | 0.93 | 0.4190 |
|  |  | 2020-04-15 | 0.075 | 0.073 | 0.0041 | 0.065 | 0.082 | 1.03 | 0.6191 |
|  |  | 2020-04-22 | 0.067 | 0.078 | 0.0041 | 0.070 | 0.086 | 0.87 | 0.0128 |
|  |  | 2020-04-29 | 0.060 | 0.067 | 0.0041 | 0.058 | 0.075 | 0.91 | 0.1343 |
|  |  | 2020-05-06 | 0.070 | 0.071 | 0.0041 | 0.063 | 0.079 | 0.99 | 0.8518 |
|  |  | 2020-05-13 | 0.069 | 0.071 | 0.0041 | 0.063 | 0.079 | 0.97 | 0.5748 |
|  |  | 2020-05-20 | 0.059 | 0.064 | 0.0042 | 0.056 | 0.072 | 0.92 | 0.2439 |
|  |  | 2020-05-27 | 0.071 | 0.073 | 0.0041 | 0.064 | 0.081 | 0.98 | 0.6816 |
|  |  | 2020-06-03 | 0.065 | 0.066 | 0.0041 | 0.058 | 0.074 | 0.99 | 0.8321 |
|  |  | 2020-06-10 | 0.074 | 0.075 | 0.0041 | 0.067 | 0.083 | 0.98 | 0.7268 |
|  |  | 2020-06-17 | 0.058 | 0.063 | 0.0041 | 0.055 | 0.071 | 0.92 | 0.2360 |
|  |  | 2020-06-24 | 0.074 | 0.073 | 0.0041 | 0.065 | 0.081 | 1.02 | 0.7163 |
|  |  | 2020-07-01 | 0.071 | 0.068 | 0.0041 | 0.060 | 0.076 | 1.04 | 0.4797 |
|  |  | 2020-07-08 | 0.065 | 0.064 | 0.0041 | 0.056 | 0.072 | 1.02 | 0.7729 |
|  |  | 2020-07-15 | 0.061 | 0.063 | 0.0041 | 0.055 | 0.071 | 0.96 | 0.5100 |
|  |  | 2020-07-22 | 0.064 | 0.063 | 0.0041 | 0.055 | 0.071 | 1.01 | 0.8368 |
|  |  | 2020-07-29 | 0.062 | 0.064 | 0.0041 | 0.056 | 0.072 | 0.98 | 0.7649 |
|  |  | 2020-08-05 | 0.069 | 0.064 | 0.0041 | 0.056 | 0.072 | 1.07 | 0.2518 |
|  |  | 2020-08-12 | 0.065 | 0.069 | 0.0041 | 0.061 | 0.077 | 0.93 | 0.2724 |
|  |  | 2020-08-19 | 0.078 | 0.071 | 0.0041 | 0.063 | 0.079 | 1.11 | 0.0667 |
|  |  | 2020-08-26 | 0.071 | 0.075 | 0.0041 | 0.067 | 0.083 | 0.95 | 0.3661 |
|  |  | 2020-09-02 | 0.073 | 0.072 | 0.0041 | 0.064 | 0.080 | 1.01 | 0.8676 |
|  |  | 2020-09-09 | 0.068 | 0.070 | 0.0041 | 0.062 | 0.078 | 0.98 | 0.7527 |
|  |  | 2020-09-16 | 0.069 | 0.072 | 0.0041 | 0.063 | 0.080 | 0.96 | 0.5019 |
|  |  | 2020-09-23 | 0.075 | 0.074 | 0.0041 | 0.066 | 0.083 | 1.01 | 0.8135 |
|  |  | 2020-09-30 | 0.073 | 0.073 | 0.0041 | 0.065 | 0.081 | 1.00 | 0.9832 |
|  |  | 2020-10-07 | 0.070 | 0.070 | 0.0041 | 0.062 | 0.078 | 1.01 | 0.8974 |
|  |  | 2020-10-14 | 0.073 | 0.073 | 0.0041 | 0.065 | 0.081 | 1.00 | 0.9349 |
|  |  | 2020-10-21 | 0.071 | 0.074 | 0.0041 | 0.066 | 0.082 | 0.96 | 0.5137 |
|  |  | 2020-10-28 | 0.076 | 0.069 | 0.0041 | 0.060 | 0.077 | 1.11 | 0.0664 |
|  |  | 2020-11-04 | 0.077 | 0.072 | 0.0041 | 0.064 | 0.081 | 1.06 | 0.3064 |
|  |  | 2020-11-11 | 0.080 | 0.071 | 0.0041 | 0.063 | 0.079 | 1.13 | 0.0284 |
|  |  | 2020-11-18 | 0.078 | 0.074 | 0.0041 | 0.066 | 0.083 | 1.05 | 0.3717 |
|  |  | 2020-11-25 | 0.083 | 0.075 | 0.0041 | 0.067 | 0.083 | 1.11 | 0.0416 |
|  |  | 2020-12-02 | 0.083 | 0.077 | 0.0041 | 0.069 | 0.085 | 1.07 | 0.1818 |
|  |  | 2020-12-09 | 0.082 | 0.078 | 0.0041 | 0.070 | 0.087 | 1.04 | 0.4225 |
|  |  | 2020-12-16 | 0.085 | 0.077 | 0.0042 | 0.069 | 0.085 | 1.10 | 0.0666 |
|  |  | 2020-12-23 | 0.052 | 0.048 | 0.0041 | 0.040 | 0.056 | 1.09 | 0.3121 |
| P01BB | Biguanides | 2020-01-01 | 0.093 | 0.11 | 0.011 | 0.090 | 0.13 | 0.83 | 0.0969 |
|  |  | 2020-01-08 | 0.14 | 0.14 | 0.011 | 0.11 | 0.16 | 1.03 | 0.7504 |
|  |  | 2020-01-15 | 0.14 | 0.13 | 0.011 | 0.11 | 0.15 | 1.04 | 0.6159 |
|  |  | 2020-01-22 | 0.14 | 0.13 | 0.011 | 0.11 | 0.15 | 1.04 | 0.6553 |
|  |  | 2020-01-29 | 0.12 | 0.12 | 0.011 | 0.10 | 0.15 | 0.97 | 0.7115 |
|  |  | 2020-02-05 | 0.10 | 0.11 | 0.011 | 0.092 | 0.14 | 0.89 | 0.2669 |
|  |  | 2020-02-12 | 0.096 | 0.10 | 0.011 | 0.081 | 0.13 | 0.92 | 0.4724 |
|  |  | 2020-02-19 | 0.080 | 0.092 | 0.011 | 0.070 | 0.11 | 0.87 | 0.2864 |
|  |  | 2020-02-26 | 0.076 | 0.087 | 0.011 | 0.065 | 0.11 | 0.88 | 0.3403 |
|  |  | 2020-03-04 | 0.053 | 0.078 | 0.011 | 0.056 | 0.100 | 0.69 | 0.0323 |
|  |  | 2020-03-11 | 0.021 | 0.072 | 0.011 | 0.049 | 0.094 | 0.29 | 1.5E-05 |
|  |  | 2020-03-18 | 0.0020 | 0.065 | 0.012 | 0.042 | 0.088 | 0.03 | 1.3E-07 |
|  |  | 2020-03-25 | 0 | 0.070 | 0.012 | 0.047 | 0.094 | 0.01 | 3.4E-08 |
|  |  | 2020-04-01 | 0 | 0.060 | 0.012 | 0.036 | 0.084 | 0.01 | 1.4E-06 |
|  |  | 2020-04-08 | 0 | 0.037 | 0.012 | 0.013 | 0.061 | 0.00 | 0.0025 |
|  |  | 2020-04-15 | 0 | 0.043 | 0.011 | 0.021 | 0.065 | 0.00 | 1.7E-04 |
|  |  | 2020-04-22 | 0 | 0.048 | 0.011 | 0.026 | 0.070 | 0.00 | 3.0E-05 |
|  |  | 2020-04-29 | 0 | 0.035 | 0.011 | 0.013 | 0.057 | 0.00 | 0.0021 |
|  |  | 2020-05-06 | 0 | 0.050 | 0.011 | 0.027 | 0.072 | 0.00 | 1.7E-05 |
|  |  | 2020-05-13 | 0 | 0.050 | 0.011 | 0.028 | 0.072 | 0.00 | 1.3E-05 |
|  |  | 2020-05-20 | 0 | 0.041 | 0.011 | 0.019 | 0.064 | 0.00 | 3.4E-04 |
|  |  | 2020-05-27 | 0 | 0.066 | 0.011 | 0.043 | 0.088 | 0.00 | 2.2E-08 |
|  |  | 2020-06-03 | 0 | 0.066 | 0.011 | 0.044 | 0.088 | 0.00 | 1.5E-08 |
|  |  | 2020-06-10 | 0 | 0.075 | 0.011 | 0.053 | 0.097 | 0.00 | 2.0E-10 |
|  |  | 2020-06-17 | 0 | 0.060 | 0.011 | 0.038 | 0.082 | 0.00 | 2.8E-07 |
|  |  | 2020-06-24 | 0 | 0.072 | 0.011 | 0.050 | 0.094 | 0.01 | 1.1E-09 |
|  |  | 2020-07-01 | 0 | 0.064 | 0.011 | 0.042 | 0.086 | 0.00 | 3.6E-08 |
|  |  | 2020-07-08 | 0 | 0.055 | 0.011 | 0.033 | 0.077 | 0.01 | 2.5E-06 |
|  |  | 2020-07-15 | 0 | 0.047 | 0.011 | 0.025 | 0.069 | 0.02 | 5.1E-05 |
|  |  | 2020-07-22 | 0 | 0.048 | 0.011 | 0.026 | 0.070 | 0.01 | 3.1E-05 |
|  |  | 2020-07-29 | 0 | 0.048 | 0.011 | 0.026 | 0.070 | 0.01 | 3.0E-05 |
|  |  | 2020-08-05 | 0 | 0.047 | 0.011 | 0.025 | 0.069 | 0.01 | 5.7E-05 |
|  |  | 2020-08-12 | 0 | 0.051 | 0.011 | 0.029 | 0.073 | 0.00 | 1.1E-05 |
|  |  | 2020-08-19 | 0.0019 | 0.058 | 0.011 | 0.036 | 0.080 | 0.03 | 1.4E-06 |
|  |  | 2020-08-26 | 0.0035 | 0.068 | 0.011 | 0.046 | 0.090 | 0.05 | 3.0E-08 |
|  |  | 2020-09-02 | 0.0018 | 0.072 | 0.011 | 0.050 | 0.094 | 0.03 | 2.5E-09 |
|  |  | 2020-09-09 | 0.0045 | 0.078 | 0.011 | 0.055 | 0.100 | 0.06 | 5.9E-10 |
|  |  | 2020-09-16 | 0.0023 | 0.083 | 0.011 | 0.061 | 0.11 | 0.03 | 9.9E-12 |
|  |  | 2020-09-23 | 0.0049 | 0.10 | 0.011 | 0.081 | 0.13 | 0.05 | 9.3E-16 |
|  |  | 2020-09-30 | 0.0040 | 0.12 | 0.011 | 0.094 | 0.14 | 0.03 | 2.9E-19 |
|  |  | 2020-10-07 | 0.0042 | 0.12 | 0.011 | 0.10 | 0.15 | 0.03 | 2.7E-21 |
|  |  | 2020-10-14 | 0.0029 | 0.14 | 0.011 | 0.12 | 0.16 | 0.02 | 2.2E-26 |
|  |  | 2020-10-21 | 0.0055 | 0.14 | 0.011 | 0.12 | 0.16 | 0.04 | 1.4E-25 |
|  |  | 2020-10-28 | 0.0048 | 0.12 | 0.011 | 0.099 | 0.14 | 0.04 | 2.0E-20 |
|  |  | 2020-11-04 | 0.0039 | 0.12 | 0.011 | 0.094 | 0.14 | 0.03 | 2.4E-19 |
|  |  | 2020-11-11 | 0.0044 | 0.11 | 0.011 | 0.091 | 0.14 | 0.04 | 1.7E-18 |
|  |  | 2020-11-18 | 0.0023 | 0.12 | 0.011 | 0.097 | 0.14 | 0.02 | 1.4E-20 |
|  |  | 2020-11-25 | 0.0052 | 0.14 | 0.011 | 0.12 | 0.17 | 0.04 | 1.1E-26 |
|  |  | 2020-12-02 | 0.0074 | 0.18 | 0.011 | 0.16 | 0.20 | 0.04 | 1.2E-35 |
|  |  | 2020-12-09 | 0.011 | 0.21 | 0.011 | 0.19 | 0.23 | 0.05 | 4.1E-43 |
|  |  | 2020-12-16 | 0.0099 | 0.20 | 0.011 | 0.18 | 0.22 | 0.05 | 9.9E-40 |
|  |  | 2020-12-23 | 0.0029 | 0.11 | 0.011 | 0.092 | 0.14 | 0.03 | 4.6E-19 |
| P01BC | Methanolquinolines | 2020-01-01 | 0.026 | 0.022 | 0.0035 | 0.015 | 0.029 | 1.17 | 0.2919 |
|  |  | 2020-01-08 | 0.028 | 0.030 | 0.0035 | 0.023 | 0.037 | 0.92 | 0.4956 |
|  |  | 2020-01-15 | 0.033 | 0.029 | 0.0035 | 0.022 | 0.036 | 1.16 | 0.1833 |
|  |  | 2020-01-22 | 0.026 | 0.030 | 0.0035 | 0.023 | 0.037 | 0.86 | 0.2421 |
|  |  | 2020-01-29 | 0.027 | 0.027 | 0.0035 | 0.021 | 0.034 | 0.98 | 0.8969 |
|  |  | 2020-02-05 | 0.022 | 0.024 | 0.0035 | 0.017 | 0.031 | 0.91 | 0.5233 |
|  |  | 2020-02-12 | 0.026 | 0.024 | 0.0035 | 0.017 | 0.030 | 1.08 | 0.5698 |
|  |  | 2020-02-19 | 0.023 | 0.023 | 0.0035 | 0.016 | 0.030 | 1.00 | 0.9924 |
|  |  | 2020-02-26 | 0.025 | 0.024 | 0.0035 | 0.017 | 0.031 | 1.03 | 0.8341 |
|  |  | 2020-03-04 | 0.022 | 0.022 | 0.0035 | 0.015 | 0.029 | 0.98 | 0.9158 |
|  |  | 2020-03-11 | 0.021 | 0.023 | 0.0036 | 0.016 | 0.030 | 0.93 | 0.6363 |
|  |  | 2020-03-18 | 0.019 | 0.021 | 0.0036 | 0.014 | 0.028 | 0.88 | 0.4673 |
|  |  | 2020-03-25 | 0.019 | 0.023 | 0.0038 | 0.015 | 0.030 | 0.83 | 0.2955 |
|  |  | 2020-04-01 | 0.016 | 0.023 | 0.0037 | 0.016 | 0.031 | 0.71 | 0.0679 |
|  |  | 2020-04-08 | 0.011 | 0.011 | 0.0037 | 0.0032 | 0.018 | 1.02 | 0.9536 |
|  |  | 2020-04-15 | 0.014 | 0.023 | 0.0035 | 0.016 | 0.030 | 0.61 | 0.0101 |
|  |  | 2020-04-22 | 0.017 | 0.024 | 0.0035 | 0.017 | 0.031 | 0.73 | 0.0593 |
|  |  | 2020-04-29 | 0.0096 | 0.022 | 0.0035 | 0.015 | 0.029 | 0.44 | 6.6E-04 |
|  |  | 2020-05-06 | 0.014 | 0.027 | 0.0035 | 0.020 | 0.034 | 0.53 | 3.2E-04 |
|  |  | 2020-05-13 | 0.013 | 0.028 | 0.0035 | 0.021 | 0.035 | 0.47 | 2.6E-05 |
|  |  | 2020-05-20 | 0.015 | 0.032 | 0.0035 | 0.025 | 0.039 | 0.46 | 2.1E-06 |
|  |  | 2020-05-27 | 0.012 | 0.043 | 0.0035 | 0.036 | 0.050 | 0.28 | 4.1E-16 |
|  |  | 2020-06-03 | 0.017 | 0.042 | 0.0035 | 0.035 | 0.049 | 0.40 | 7.0E-12 |
|  |  | 2020-06-10 | 0.016 | 0.045 | 0.0035 | 0.039 | 0.052 | 0.35 | 4.2E-15 |
|  |  | 2020-06-17 | 0.014 | 0.034 | 0.0035 | 0.027 | 0.041 | 0.40 | 2.0E-08 |
|  |  | 2020-06-24 | 0.015 | 0.038 | 0.0035 | 0.031 | 0.044 | 0.39 | 4.0E-10 |
|  |  | 2020-07-01 | 0.016 | 0.030 | 0.0035 | 0.023 | 0.037 | 0.54 | 1.1E-04 |
|  |  | 2020-07-08 | 0.013 | 0.025 | 0.0035 | 0.018 | 0.032 | 0.52 | 6.2E-04 |
|  |  | 2020-07-15 | 0.016 | 0.023 | 0.0035 | 0.016 | 0.030 | 0.70 | 0.0480 |
|  |  | 2020-07-22 | 0.013 | 0.022 | 0.0035 | 0.015 | 0.029 | 0.58 | 0.0076 |
|  |  | 2020-07-29 | 0.017 | 0.021 | 0.0035 | 0.014 | 0.028 | 0.79 | 0.2122 |
|  |  | 2020-08-05 | 0.015 | 0.021 | 0.0035 | 0.014 | 0.028 | 0.71 | 0.0775 |
|  |  | 2020-08-12 | 0.017 | 0.022 | 0.0035 | 0.016 | 0.029 | 0.75 | 0.1101 |
|  |  | 2020-08-19 | 0.016 | 0.025 | 0.0035 | 0.019 | 0.032 | 0.64 | 0.0091 |
|  |  | 2020-08-26 | 0.020 | 0.027 | 0.0035 | 0.020 | 0.034 | 0.73 | 0.0392 |
|  |  | 2020-09-02 | 0.013 | 0.022 | 0.0035 | 0.015 | 0.029 | 0.60 | 0.0119 |
|  |  | 2020-09-09 | 0.015 | 0.023 | 0.0035 | 0.016 | 0.030 | 0.67 | 0.0302 |
|  |  | 2020-09-16 | 0.017 | 0.024 | 0.0035 | 0.018 | 0.031 | 0.70 | 0.0366 |
|  |  | 2020-09-23 | 0.016 | 0.026 | 0.0035 | 0.019 | 0.033 | 0.64 | 0.0083 |
|  |  | 2020-09-30 | 0.013 | 0.028 | 0.0035 | 0.021 | 0.035 | 0.48 | 4.4E-05 |
|  |  | 2020-10-07 | 0.016 | 0.025 | 0.0035 | 0.018 | 0.031 | 0.63 | 0.0097 |
|  |  | 2020-10-14 | 0.018 | 0.026 | 0.0035 | 0.019 | 0.033 | 0.69 | 0.0202 |
|  |  | 2020-10-21 | 0.018 | 0.028 | 0.0035 | 0.021 | 0.035 | 0.64 | 0.0047 |
|  |  | 2020-10-28 | 0.015 | 0.026 | 0.0035 | 0.019 | 0.033 | 0.56 | 0.0011 |
|  |  | 2020-11-04 | 0.016 | 0.028 | 0.0035 | 0.021 | 0.035 | 0.58 | 9.1E-04 |
|  |  | 2020-11-11 | 0.015 | 0.030 | 0.0035 | 0.023 | 0.037 | 0.50 | 3.3E-05 |
|  |  | 2020-11-18 | 0.017 | 0.036 | 0.0035 | 0.029 | 0.043 | 0.48 | 2.0E-07 |
|  |  | 2020-11-25 | 0.017 | 0.045 | 0.0035 | 0.038 | 0.052 | 0.38 | 1.5E-13 |
|  |  | 2020-12-02 | 0.022 | 0.047 | 0.0035 | 0.040 | 0.054 | 0.47 | 1.4E-11 |
|  |  | 2020-12-09 | 0.021 | 0.042 | 0.0035 | 0.035 | 0.049 | 0.51 | 1.7E-08 |
|  |  | 2020-12-16 | 0.019 | 0.039 | 0.0035 | 0.032 | 0.046 | 0.50 | 1.4E-07 |
|  |  | 2020-12-23 | 0.012 | 0.019 | 0.0035 | 0.012 | 0.026 | 0.63 | 0.0492 |
|  |  |  |  |  |  |  |  |  |  |

*Abbreviations: N (Number), Confidence limit (CL)*, *Anatomical Therapeutic Code (ATC)*

**p-values are presented uncorrected for multiple comparisons*

Table S6 Total volumes of antimicrobials sold to inpatient care institutions in DDDs per 1000 inhabitants, by ATC therapeutic subgroup and year, Sweden, 2015-2020

|  | | Volume sold per 1000 inhabitants, DDDs | | | | | |
| --- | --- | --- | --- | --- | --- | --- | --- |
|  |  | 2015 | 2016 | 2017 | 2018 | 2019 | 2020 |
| J01 | Antibacterials for systemic use | 552.7 | 543.4 | 524.7 | 508.1 | 507.4 | 492.0 |
| J02 | Antimycotics for systemic use | 22.1 | 22.5 | 22.4 | 21.4 | 20.3 | 21.3 |
| J04 | Antimycobacterials | 50.5 | 76.0 | 57.8 | 48.5 | 48.4 | 42.5 |
| J05 | Antivirals for systemic use | 13.1 | 14.6 | 14.3 | 16.4 | 16.2 | 14.1 |
| P01 | Antiprotozoals | 11.4 | 10.6 | 10.1 | 9.7 | 9.7 | 22.0 |
| P02 | Anthelmintics | 0.1 | 0.1 | 0.0 | 0.0 | 0.0 | 0.0 |
|  |  |  |  |  |  |  |  |

Table S7 Observed versus predicted weekly volumes of antimicrobials sold per 1000 inhabitants by ATC therapeutic subgroup, Sweden, 2020

|  | | | Volume sold per 1000 inhabitants, DDDs | | | | | | |
| --- | --- | --- | --- | --- | --- | --- | --- | --- | --- |
|  |  | Week | Observed | Predicted | Standard  error | Lower  95% CL | Upper  95% CL | Ratio  (observed/predicted) | p-value* |
| J01 | Antibacterials for systemic use | 2020-01-01 | 8.4 | 6.7 | 0.74 | 5.3 | 8.2 | 1.25 | 0.0266 |
|  |  | 2020-01-08 | 12 | 9.9 | 0.74 | 8.5 | 11 | 1.16 | 0.0286 |
|  |  | 2020-01-15 | 9.4 | 9.9 | 0.74 | 8.4 | 11 | 0.95 | 0.5379 |
|  |  | 2020-01-22 | 9.6 | 9.6 | 0.74 | 8.2 | 11 | 1.00 | 0.9496 |
|  |  | 2020-01-29 | 9.8 | 9.7 | 0.74 | 8.3 | 11 | 1.00 | 0.9493 |
|  |  | 2020-02-05 | 9.8 | 10 | 0.74 | 8.5 | 11 | 0.98 | 0.8349 |
|  |  | 2020-02-12 | 9.5 | 10 | 0.74 | 8.6 | 12 | 0.94 | 0.4275 |
|  |  | 2020-02-19 | 11 | 10 | 0.74 | 8.8 | 12 | 1.04 | 0.6242 |
|  |  | 2020-02-26 | 10 | 10 | 0.74 | 8.6 | 11 | 1.00 | 0.9782 |
|  |  | 2020-03-04 | 12 | 10 | 0.74 | 8.6 | 12 | 1.17 | 0.0237 |
|  |  | 2020-03-11 | 16 | 9.8 | 0.75 | 8.3 | 11 | 1.65 | 3.7E-15 |
|  |  | 2020-03-18 | 19 | 9.7 | 0.76 | 8.2 | 11 | 1.96 | 2.1E-26 |
|  |  | 2020-03-25 | 17 | 10 | 0.80 | 8.9 | 12 | 1.67 | 5.9E-16 |
|  |  | 2020-04-01 | 16 | 12 | 0.79 | 10 | 13 | 1.35 | 7.5E-07 |
|  |  | 2020-04-08 | 11 | 7.8 | 0.79 | 6.2 | 9.4 | 1.37 | 3.5E-04 |
|  |  | 2020-04-15 | 11 | 9.6 | 0.74 | 8.1 | 11 | 1.15 | 0.0478 |
|  |  | 2020-04-22 | 8.8 | 10 | 0.74 | 8.8 | 12 | 0.86 | 0.0522 |
|  |  | 2020-04-29 | 7.4 | 8.9 | 0.74 | 7.4 | 10 | 0.84 | 0.0539 |
|  |  | 2020-05-06 | 8.3 | 10 | 0.74 | 8.9 | 12 | 0.80 | 0.0059 |
|  |  | 2020-05-13 | 8.1 | 9.9 | 0.74 | 8.4 | 11 | 0.82 | 0.0148 |
|  |  | 2020-05-20 | 6.5 | 8.6 | 0.74 | 7.2 | 10 | 0.76 | 0.0052 |
|  |  | 2020-05-27 | 8.2 | 10 | 0.74 | 8.8 | 12 | 0.80 | 0.0074 |
|  |  | 2020-06-03 | 8.2 | 9.2 | 0.74 | 7.7 | 11 | 0.90 | 0.1982 |
|  |  | 2020-06-10 | 9.2 | 11 | 0.74 | 9.5 | 12 | 0.84 | 0.0171 |
|  |  | 2020-06-17 | 7.6 | 8.7 | 0.74 | 7.2 | 10 | 0.87 | 0.1349 |
|  |  | 2020-06-24 | 7.9 | 9.8 | 0.74 | 8.4 | 11 | 0.81 | 0.0114 |
|  |  | 2020-07-01 | 7.0 | 8.3 | 0.74 | 6.9 | 9.8 | 0.84 | 0.0734 |
|  |  | 2020-07-08 | 6.7 | 7.8 | 0.74 | 6.4 | 9.3 | 0.86 | 0.1341 |
|  |  | 2020-07-15 | 6.6 | 7.5 | 0.74 | 6.1 | 9.0 | 0.88 | 0.2040 |
|  |  | 2020-07-22 | 6.5 | 7.5 | 0.74 | 6.0 | 8.9 | 0.87 | 0.1822 |
|  |  | 2020-07-29 | 6.4 | 7.2 | 0.74 | 5.8 | 8.7 | 0.88 | 0.2579 |
|  |  | 2020-08-05 | 7.1 | 7.6 | 0.74 | 6.1 | 9.0 | 0.94 | 0.5196 |
|  |  | 2020-08-12 | 7.2 | 8.2 | 0.74 | 6.7 | 9.6 | 0.88 | 0.1929 |
|  |  | 2020-08-19 | 7.6 | 8.7 | 0.74 | 7.2 | 10 | 0.87 | 0.1399 |
|  |  | 2020-08-26 | 8.4 | 8.9 | 0.74 | 7.5 | 10 | 0.93 | 0.4236 |
|  |  | 2020-09-02 | 8.3 | 8.8 | 0.74 | 7.4 | 10 | 0.94 | 0.5081 |
|  |  | 2020-09-09 | 8.5 | 9.1 | 0.74 | 7.6 | 11 | 0.94 | 0.4357 |
|  |  | 2020-09-16 | 8.5 | 9.4 | 0.74 | 8.0 | 11 | 0.90 | 0.2089 |
|  |  | 2020-09-23 | 8.5 | 9.7 | 0.74 | 8.2 | 11 | 0.88 | 0.1110 |
|  |  | 2020-09-30 | 8.6 | 9.2 | 0.74 | 7.8 | 11 | 0.93 | 0.3956 |
|  |  | 2020-10-07 | 8.2 | 9.4 | 0.74 | 8.0 | 11 | 0.87 | 0.0866 |
|  |  | 2020-10-14 | 8.8 | 9.5 | 0.74 | 8.0 | 11 | 0.92 | 0.3169 |
|  |  | 2020-10-21 | 8.9 | 9.5 | 0.74 | 8.0 | 11 | 0.93 | 0.3885 |
|  |  | 2020-10-28 | 8.8 | 9.2 | 0.74 | 7.7 | 11 | 0.96 | 0.5780 |
|  |  | 2020-11-04 | 9.1 | 9.8 | 0.74 | 8.3 | 11 | 0.94 | 0.3928 |
|  |  | 2020-11-11 | 9.4 | 9.4 | 0.74 | 7.9 | 11 | 1.00 | 0.9875 |
|  |  | 2020-11-18 | 9.6 | 9.9 | 0.74 | 8.5 | 11 | 0.96 | 0.6337 |
|  |  | 2020-11-25 | 9.5 | 9.8 | 0.74 | 8.3 | 11 | 0.97 | 0.6635 |
|  |  | 2020-12-02 | 10 | 10 | 0.74 | 8.9 | 12 | 0.97 | 0.6869 |
|  |  | 2020-12-09 | 11 | 13 | 0.74 | 11 | 14 | 0.90 | 0.0848 |
|  |  | 2020-12-16 | 15 | 13 | 0.75 | 12 | 15 | 1.13 | 0.0292 |
|  |  | 2020-12-23 | 6.9 | 6.6 | 0.74 | 5.1 | 8.1 | 1.05 | 0.6394 |
| J02 | Antimycotics for systemic use | 2020-01-01 | 0.38 | 0.28 | 0.054 | 0.18 | 0.39 | 1.32 | 0.0966 |
|  |  | 2020-01-08 | 0.51 | 0.36 | 0.054 | 0.26 | 0.47 | 1.41 | 0.0064 |
|  |  | 2020-01-15 | 0.40 | 0.38 | 0.054 | 0.27 | 0.49 | 1.06 | 0.6819 |
|  |  | 2020-01-22 | 0.42 | 0.41 | 0.054 | 0.30 | 0.52 | 1.02 | 0.8662 |
|  |  | 2020-01-29 | 0.45 | 0.41 | 0.054 | 0.31 | 0.52 | 1.09 | 0.5112 |
|  |  | 2020-02-05 | 0.37 | 0.41 | 0.054 | 0.31 | 0.52 | 0.89 | 0.3953 |
|  |  | 2020-02-12 | 0.42 | 0.40 | 0.054 | 0.30 | 0.51 | 1.04 | 0.7890 |
|  |  | 2020-02-19 | 0.43 | 0.38 | 0.054 | 0.28 | 0.49 | 1.12 | 0.4030 |
|  |  | 2020-02-26 | 0.38 | 0.42 | 0.054 | 0.31 | 0.52 | 0.90 | 0.4599 |
|  |  | 2020-03-04 | 0.41 | 0.41 | 0.054 | 0.31 | 0.52 | 0.99 | 0.9437 |
|  |  | 2020-03-11 | 0.59 | 0.41 | 0.055 | 0.31 | 0.52 | 1.43 | 0.0015 |
|  |  | 2020-03-18 | 0.54 | 0.43 | 0.056 | 0.32 | 0.54 | 1.27 | 0.0376 |
|  |  | 2020-03-25 | 0.46 | 0.39 | 0.058 | 0.28 | 0.51 | 1.17 | 0.2402 |
|  |  | 2020-04-01 | 0.53 | 0.52 | 0.058 | 0.40 | 0.63 | 1.03 | 0.8192 |
|  |  | 2020-04-08 | 0.33 | 0.35 | 0.058 | 0.24 | 0.46 | 0.93 | 0.6891 |
|  |  | 2020-04-15 | 0.42 | 0.41 | 0.054 | 0.30 | 0.51 | 1.02 | 0.8648 |
|  |  | 2020-04-22 | 0.56 | 0.42 | 0.054 | 0.31 | 0.52 | 1.35 | 0.0079 |
|  |  | 2020-04-29 | 0.44 | 0.36 | 0.054 | 0.25 | 0.47 | 1.21 | 0.1619 |
|  |  | 2020-05-06 | 0.36 | 0.42 | 0.054 | 0.31 | 0.52 | 0.86 | 0.2721 |
|  |  | 2020-05-13 | 0.43 | 0.42 | 0.054 | 0.32 | 0.53 | 1.02 | 0.8597 |
|  |  | 2020-05-20 | 0.30 | 0.35 | 0.054 | 0.25 | 0.46 | 0.84 | 0.3012 |
|  |  | 2020-05-27 | 0.37 | 0.40 | 0.054 | 0.29 | 0.50 | 0.94 | 0.6502 |
|  |  | 2020-06-03 | 0.49 | 0.36 | 0.054 | 0.25 | 0.47 | 1.36 | 0.0179 |
|  |  | 2020-06-10 | 0.40 | 0.41 | 0.054 | 0.30 | 0.52 | 0.97 | 0.8316 |
|  |  | 2020-06-17 | 0.31 | 0.38 | 0.054 | 0.27 | 0.49 | 0.82 | 0.2058 |
|  |  | 2020-06-24 | 0.39 | 0.39 | 0.054 | 0.29 | 0.50 | 0.98 | 0.9059 |
|  |  | 2020-07-01 | 0.32 | 0.37 | 0.054 | 0.26 | 0.47 | 0.89 | 0.4580 |
|  |  | 2020-07-08 | 0.31 | 0.37 | 0.054 | 0.26 | 0.48 | 0.84 | 0.2827 |
|  |  | 2020-07-15 | 0.39 | 0.36 | 0.054 | 0.26 | 0.47 | 1.07 | 0.6398 |
|  |  | 2020-07-22 | 0.35 | 0.37 | 0.054 | 0.27 | 0.48 | 0.94 | 0.6736 |
|  |  | 2020-07-29 | 0.36 | 0.35 | 0.054 | 0.25 | 0.46 | 1.02 | 0.9072 |
|  |  | 2020-08-05 | 0.30 | 0.35 | 0.054 | 0.24 | 0.45 | 0.87 | 0.3950 |
|  |  | 2020-08-12 | 0.32 | 0.36 | 0.054 | 0.25 | 0.47 | 0.90 | 0.4951 |
|  |  | 2020-08-19 | 0.36 | 0.39 | 0.054 | 0.28 | 0.49 | 0.92 | 0.5809 |
|  |  | 2020-08-26 | 0.32 | 0.37 | 0.054 | 0.27 | 0.48 | 0.87 | 0.3533 |
|  |  | 2020-09-02 | 0.33 | 0.37 | 0.054 | 0.27 | 0.48 | 0.87 | 0.3778 |
|  |  | 2020-09-09 | 0.37 | 0.38 | 0.054 | 0.27 | 0.48 | 0.98 | 0.8835 |
|  |  | 2020-09-16 | 0.35 | 0.40 | 0.054 | 0.29 | 0.50 | 0.88 | 0.3875 |
|  |  | 2020-09-23 | 0.38 | 0.41 | 0.054 | 0.30 | 0.52 | 0.93 | 0.5791 |
|  |  | 2020-09-30 | 0.36 | 0.41 | 0.054 | 0.30 | 0.52 | 0.88 | 0.3588 |
|  |  | 2020-10-07 | 0.37 | 0.43 | 0.054 | 0.32 | 0.53 | 0.88 | 0.3256 |
|  |  | 2020-10-14 | 0.32 | 0.38 | 0.054 | 0.27 | 0.49 | 0.85 | 0.2818 |
|  |  | 2020-10-21 | 0.44 | 0.41 | 0.054 | 0.31 | 0.52 | 1.06 | 0.6393 |
|  |  | 2020-10-28 | 0.45 | 0.37 | 0.054 | 0.26 | 0.47 | 1.23 | 0.1214 |
|  |  | 2020-11-04 | 0.39 | 0.38 | 0.054 | 0.28 | 0.49 | 1.03 | 0.8487 |
|  |  | 2020-11-11 | 0.40 | 0.39 | 0.054 | 0.28 | 0.50 | 1.03 | 0.8358 |
|  |  | 2020-11-18 | 0.58 | 0.41 | 0.054 | 0.31 | 0.52 | 1.40 | 0.0026 |
|  |  | 2020-11-25 | 0.43 | 0.39 | 0.054 | 0.29 | 0.50 | 1.09 | 0.5320 |
|  |  | 2020-12-02 | 0.42 | 0.41 | 0.054 | 0.31 | 0.52 | 1.03 | 0.8329 |
|  |  | 2020-12-09 | 0.46 | 0.43 | 0.054 | 0.32 | 0.54 | 1.08 | 0.5247 |
|  |  | 2020-12-16 | 0.68 | 0.47 | 0.055 | 0.37 | 0.58 | 1.43 | 2.3E-04 |
|  |  | 2020-12-23 | 0.44 | 0.30 | 0.054 | 0.20 | 0.41 | 1.45 | 0.0131 |
| J04 | Antimycobacterials | 2020-01-01 | 0.18 | 0.33 | 0.44 | -1 | 1.2 | 0.54 | 0.7308 |
|  |  | 2020-01-08 | 1.8 | 0.91 | 0.44 | 0.038 | 1.8 | 2.02 | 0.0371 |
|  |  | 2020-01-15 | 1.4 | 0.79 | 0.44 | -0 | 1.7 | 1.74 | 0.1856 |
|  |  | 2020-01-22 | 1.0 | 1.1 | 0.44 | 0.23 | 2.0 | 0.91 | 0.8241 |
|  |  | 2020-01-29 | 0.85 | 0.65 | 0.44 | -0 | 1.5 | 1.31 | 0.6489 |
|  |  | 2020-02-05 | 0.84 | 0.98 | 0.44 | 0.11 | 1.9 | 0.86 | 0.7571 |
|  |  | 2020-02-12 | 0.80 | 0.94 | 0.44 | 0.070 | 1.8 | 0.85 | 0.7561 |
|  |  | 2020-02-19 | 0.90 | 1.1 | 0.44 | 0.23 | 2.0 | 0.82 | 0.6463 |
|  |  | 2020-02-26 | 0.98 | 0.79 | 0.44 | -0 | 1.7 | 1.23 | 0.6748 |
|  |  | 2020-03-04 | 1.3 | 0.77 | 0.44 | -0 | 1.6 | 1.66 | 0.2512 |
|  |  | 2020-03-11 | 1.7 | 0.88 | 0.45 | -0 | 1.8 | 1.93 | 0.0715 |
|  |  | 2020-03-18 | 0.69 | 1.1 | 0.46 | 0.20 | 2.0 | 0.63 | 0.3746 |
|  |  | 2020-03-25 | 0.72 | 1.0 | 0.48 | 0.063 | 1.9 | 0.72 | 0.5527 |
|  |  | 2020-04-01 | 1.2 | 0.60 | 0.47 | -0 | 1.5 | 2.01 | 0.2001 |
|  |  | 2020-04-08 | 0.39 | 0.44 | 0.47 | -0 | 1.4 | 0.89 | 0.9225 |
|  |  | 2020-04-15 | 0.44 | 1.3 | 0.44 | 0.47 | 2.2 | 0.32 | 0.0418 |
|  |  | 2020-04-22 | 0.76 | 0.92 | 0.44 | 0.050 | 1.8 | 0.82 | 0.7152 |
|  |  | 2020-04-29 | 0.40 | 0.72 | 0.44 | -0 | 1.6 | 0.56 | 0.4735 |
|  |  | 2020-05-06 | 0.67 | 0.92 | 0.44 | 0.048 | 1.8 | 0.72 | 0.5657 |
|  |  | 2020-05-13 | 0.53 | 0.99 | 0.44 | 0.12 | 1.9 | 0.54 | 0.2972 |
|  |  | 2020-05-20 | 1.0 | 0.77 | 0.45 | -0 | 1.6 | 1.31 | 0.5977 |
|  |  | 2020-05-27 | 0.72 | 1.0 | 0.44 | 0.14 | 1.9 | 0.71 | 0.5102 |
|  |  | 2020-06-03 | 0.92 | 1.3 | 0.44 | 0.42 | 2.2 | 0.71 | 0.4009 |
|  |  | 2020-06-10 | 0.60 | 1.2 | 0.44 | 0.29 | 2.0 | 0.52 | 0.2047 |
|  |  | 2020-06-17 | 1.1 | 0.77 | 0.44 | -0 | 1.6 | 1.41 | 0.4705 |
|  |  | 2020-06-24 | 0.82 | 0.82 | 0.44 | -0 | 1.7 | 1.00 | 0.9940 |
|  |  | 2020-07-01 | 0.82 | 0.75 | 0.44 | -0 | 1.6 | 1.10 | 0.8714 |
|  |  | 2020-07-08 | 0.66 | 0.89 | 0.44 | 0.018 | 1.8 | 0.75 | 0.6085 |
|  |  | 2020-07-15 | 0.53 | 0.80 | 0.44 | -0 | 1.7 | 0.66 | 0.5379 |
|  |  | 2020-07-22 | 0.55 | 0.69 | 0.44 | -0 | 1.6 | 0.80 | 0.7624 |
|  |  | 2020-07-29 | 1.1 | 0.78 | 0.44 | -0 | 1.7 | 1.39 | 0.4932 |
|  |  | 2020-08-05 | 0.24 | 0.66 | 0.44 | -0 | 1.5 | 0.36 | 0.3432 |
|  |  | 2020-08-12 | 0.59 | 0.50 | 0.44 | -0 | 1.4 | 1.18 | 0.8346 |
|  |  | 2020-08-19 | 0.80 | 1.2 | 0.44 | 0.35 | 2.1 | 0.66 | 0.3485 |
|  |  | 2020-08-26 | 0.69 | 0.72 | 0.44 | -0 | 1.6 | 0.96 | 0.9461 |
|  |  | 2020-09-02 | 0.64 | 1.1 | 0.44 | 0.19 | 1.9 | 0.60 | 0.3376 |
|  |  | 2020-09-09 | 1.4 | 0.81 | 0.44 | -0 | 1.7 | 1.74 | 0.1737 |
|  |  | 2020-09-16 | 0.26 | 0.93 | 0.44 | 0.054 | 1.8 | 0.28 | 0.1329 |
|  |  | 2020-09-23 | 0.74 | 0.94 | 0.44 | 0.070 | 1.8 | 0.79 | 0.6510 |
|  |  | 2020-09-30 | 0.52 | 0.94 | 0.44 | 0.073 | 1.8 | 0.55 | 0.3394 |
|  |  | 2020-10-07 | 0.77 | 0.90 | 0.44 | 0.025 | 1.8 | 0.86 | 0.7775 |
|  |  | 2020-10-14 | 0.74 | 0.94 | 0.44 | 0.067 | 1.8 | 0.79 | 0.6567 |
|  |  | 2020-10-21 | 0.82 | 0.88 | 0.44 | 0.011 | 1.8 | 0.92 | 0.8799 |
|  |  | 2020-10-28 | 1.0 | 0.74 | 0.44 | -0 | 1.6 | 1.39 | 0.5200 |
|  |  | 2020-11-04 | 0.35 | 1.0 | 0.44 | 0.15 | 1.9 | 0.34 | 0.1316 |
|  |  | 2020-11-11 | 0.59 | 1.2 | 0.44 | 0.38 | 2.1 | 0.47 | 0.1397 |
|  |  | 2020-11-18 | 1.0 | 1.1 | 0.44 | 0.23 | 2.0 | 0.92 | 0.8501 |
|  |  | 2020-11-25 | 1.4 | 0.90 | 0.44 | 0.033 | 1.8 | 1.56 | 0.2567 |
|  |  | 2020-12-02 | 0.56 | 0.99 | 0.44 | 0.11 | 1.9 | 0.56 | 0.3315 |
|  |  | 2020-12-09 | 1.4 | 1.3 | 0.44 | 0.46 | 2.2 | 1.07 | 0.8299 |
|  |  | 2020-12-16 | 0.78 | 1.3 | 0.45 | 0.43 | 2.2 | 0.59 | 0.2289 |
|  |  | 2020-12-23 | 0.72 | 0.39 | 0.44 | -0 | 1.3 | 1.83 | 0.4641 |
| J05 | Antivirals for systemic use | 2020-01-01 | 0.30 | 0.41 | 0.070 | 0.28 | 0.55 | 0.74 | 0.1188 |
|  |  | 2020-01-08 | 0.33 | 0.48 | 0.069 | 0.34 | 0.61 | 0.69 | 0.0337 |
|  |  | 2020-01-15 | 0.27 | 0.39 | 0.069 | 0.25 | 0.52 | 0.69 | 0.0827 |
|  |  | 2020-01-22 | 0.28 | 0.40 | 0.069 | 0.26 | 0.54 | 0.69 | 0.0706 |
|  |  | 2020-01-29 | 0.34 | 0.45 | 0.069 | 0.32 | 0.59 | 0.75 | 0.1036 |
|  |  | 2020-02-05 | 0.36 | 0.50 | 0.069 | 0.36 | 0.64 | 0.71 | 0.0379 |
|  |  | 2020-02-12 | 0.36 | 0.58 | 0.069 | 0.44 | 0.71 | 0.62 | 0.0019 |
|  |  | 2020-02-19 | 0.37 | 0.57 | 0.069 | 0.43 | 0.71 | 0.65 | 0.0050 |
|  |  | 2020-02-26 | 0.43 | 0.51 | 0.069 | 0.37 | 0.64 | 0.86 | 0.3094 |
|  |  | 2020-03-04 | 0.35 | 0.47 | 0.069 | 0.33 | 0.61 | 0.74 | 0.0792 |
|  |  | 2020-03-11 | 0.56 | 0.40 | 0.071 | 0.26 | 0.54 | 1.41 | 0.0220 |
|  |  | 2020-03-18 | 0.64 | 0.42 | 0.071 | 0.28 | 0.56 | 1.53 | 0.0024 |
|  |  | 2020-03-25 | 0.35 | 0.45 | 0.075 | 0.31 | 0.60 | 0.78 | 0.1822 |
|  |  | 2020-04-01 | 0.28 | 0.41 | 0.074 | 0.26 | 0.55 | 0.68 | 0.0852 |
|  |  | 2020-04-08 | 0.22 | 0.31 | 0.074 | 0.16 | 0.46 | 0.70 | 0.2071 |
|  |  | 2020-04-15 | 0.22 | 0.31 | 0.070 | 0.17 | 0.45 | 0.71 | 0.2054 |
|  |  | 2020-04-22 | 0.21 | 0.35 | 0.070 | 0.21 | 0.49 | 0.61 | 0.0526 |
|  |  | 2020-04-29 | 0.22 | 0.23 | 0.070 | 0.095 | 0.37 | 0.93 | 0.8162 |
|  |  | 2020-05-06 | 0.25 | 0.30 | 0.070 | 0.16 | 0.44 | 0.83 | 0.4720 |
|  |  | 2020-05-13 | 0.25 | 0.30 | 0.069 | 0.16 | 0.44 | 0.84 | 0.4870 |
|  |  | 2020-05-20 | 0.17 | 0.26 | 0.070 | 0.12 | 0.39 | 0.66 | 0.2156 |
|  |  | 2020-05-27 | 0.18 | 0.28 | 0.070 | 0.14 | 0.42 | 0.65 | 0.1644 |
|  |  | 2020-06-03 | 0.20 | 0.29 | 0.069 | 0.16 | 0.43 | 0.69 | 0.1957 |
|  |  | 2020-06-10 | 0.23 | 0.29 | 0.069 | 0.15 | 0.42 | 0.82 | 0.4553 |
|  |  | 2020-06-17 | 0.26 | 0.23 | 0.070 | 0.097 | 0.37 | 1.10 | 0.7255 |
|  |  | 2020-06-24 | 0.26 | 0.30 | 0.070 | 0.16 | 0.44 | 0.85 | 0.5072 |
|  |  | 2020-07-01 | 0.24 | 0.25 | 0.069 | 0.12 | 0.39 | 0.94 | 0.8257 |
|  |  | 2020-07-08 | 0.24 | 0.24 | 0.069 | 0.10 | 0.37 | 1.01 | 0.9639 |
|  |  | 2020-07-15 | 0.19 | 0.23 | 0.069 | 0.092 | 0.37 | 0.82 | 0.5608 |
|  |  | 2020-07-22 | 0.19 | 0.25 | 0.069 | 0.11 | 0.39 | 0.76 | 0.3858 |
|  |  | 2020-07-29 | 0.16 | 0.22 | 0.069 | 0.084 | 0.36 | 0.71 | 0.3534 |
|  |  | 2020-08-05 | 0.17 | 0.22 | 0.069 | 0.086 | 0.36 | 0.76 | 0.4428 |
|  |  | 2020-08-12 | 0.21 | 0.26 | 0.069 | 0.13 | 0.40 | 0.79 | 0.4363 |
|  |  | 2020-08-19 | 0.17 | 0.24 | 0.069 | 0.10 | 0.37 | 0.70 | 0.3111 |
|  |  | 2020-08-26 | 0.26 | 0.29 | 0.069 | 0.15 | 0.43 | 0.91 | 0.6946 |
|  |  | 2020-09-02 | 0.15 | 0.26 | 0.069 | 0.13 | 0.40 | 0.58 | 0.1089 |
|  |  | 2020-09-09 | 0.29 | 0.26 | 0.069 | 0.12 | 0.39 | 1.11 | 0.6749 |
|  |  | 2020-09-16 | 0.18 | 0.26 | 0.069 | 0.12 | 0.39 | 0.72 | 0.3007 |
|  |  | 2020-09-23 | 0.29 | 0.26 | 0.069 | 0.12 | 0.39 | 1.15 | 0.5791 |
|  |  | 2020-09-30 | 0.22 | 0.26 | 0.069 | 0.12 | 0.40 | 0.86 | 0.5924 |
|  |  | 2020-10-07 | 0.20 | 0.29 | 0.069 | 0.15 | 0.43 | 0.70 | 0.2084 |
|  |  | 2020-10-14 | 0.24 | 0.28 | 0.069 | 0.14 | 0.41 | 0.87 | 0.5968 |
|  |  | 2020-10-21 | 0.18 | 0.29 | 0.069 | 0.16 | 0.43 | 0.63 | 0.1203 |
|  |  | 2020-10-28 | 0.29 | 0.29 | 0.069 | 0.15 | 0.43 | 1.00 | 0.9862 |
|  |  | 2020-11-04 | 0.28 | 0.28 | 0.069 | 0.14 | 0.42 | 0.98 | 0.9490 |
|  |  | 2020-11-11 | 0.26 | 0.30 | 0.069 | 0.16 | 0.43 | 0.89 | 0.6507 |
|  |  | 2020-11-18 | 0.29 | 0.32 | 0.069 | 0.18 | 0.46 | 0.90 | 0.6626 |
|  |  | 2020-11-25 | 0.37 | 0.36 | 0.069 | 0.22 | 0.50 | 1.04 | 0.8553 |
|  |  | 2020-12-02 | 0.29 | 0.35 | 0.069 | 0.21 | 0.48 | 0.83 | 0.4083 |
|  |  | 2020-12-09 | 0.29 | 0.38 | 0.069 | 0.24 | 0.51 | 0.76 | 0.1937 |
|  |  | 2020-12-16 | 0.38 | 0.52 | 0.070 | 0.38 | 0.66 | 0.73 | 0.0515 |
|  |  | 2020-12-23 | 0.22 | 0.31 | 0.070 | 0.18 | 0.45 | 0.69 | 0.1637 |
| P01 | Antiprotozoals | 2020-01-01 | 0.17 | 0.13 | 0.027 | 0.071 | 0.18 | 1.40 | 0.0718 |
|  |  | 2020-01-08 | 0.16 | 0.18 | 0.027 | 0.12 | 0.23 | 0.88 | 0.4448 |
|  |  | 2020-01-15 | 0.18 | 0.17 | 0.027 | 0.11 | 0.22 | 1.09 | 0.5879 |
|  |  | 2020-01-22 | 0.19 | 0.17 | 0.027 | 0.11 | 0.22 | 1.11 | 0.4997 |
|  |  | 2020-01-29 | 0.15 | 0.17 | 0.027 | 0.12 | 0.22 | 0.89 | 0.5063 |
|  |  | 2020-02-05 | 0.20 | 0.17 | 0.027 | 0.11 | 0.22 | 1.17 | 0.3029 |
|  |  | 2020-02-12 | 0.19 | 0.19 | 0.027 | 0.13 | 0.24 | 1.01 | 0.9704 |
|  |  | 2020-02-19 | 0.19 | 0.18 | 0.027 | 0.12 | 0.23 | 1.08 | 0.6157 |
|  |  | 2020-02-26 | 0.21 | 0.19 | 0.027 | 0.14 | 0.25 | 1.09 | 0.5085 |
|  |  | 2020-03-04 | 0.25 | 0.18 | 0.027 | 0.13 | 0.24 | 1.38 | 0.0114 |
|  |  | 2020-03-11 | 0.67 | 0.18 | 0.028 | 0.12 | 0.23 | 3.75 | 2.1E-42 |
|  |  | 2020-03-18 | 5.8 | 0.18 | 0.028 | 0.12 | 0.23 | 32.52 | 5.1E-232 |
|  |  | 2020-03-25 | 5.9 | 0.18 | 0.029 | 0.12 | 0.24 | 32.20 | 1.5E-229 |
|  |  | 2020-04-01 | 1.2 | 0.21 | 0.029 | 0.15 | 0.27 | 5.63 | 5.9E-83 |
|  |  | 2020-04-08 | 0.22 | 0.14 | 0.029 | 0.081 | 0.20 | 1.55 | 0.0091 |
|  |  | 2020-04-15 | 0.33 | 0.19 | 0.027 | 0.14 | 0.24 | 1.75 | 5.3E-07 |
|  |  | 2020-04-22 | 0.15 | 0.24 | 0.027 | 0.18 | 0.29 | 0.65 | 0.0029 |
|  |  | 2020-04-29 | 0.13 | 0.17 | 0.027 | 0.12 | 0.23 | 0.77 | 0.1485 |
|  |  | 2020-05-06 | 0.17 | 0.18 | 0.027 | 0.13 | 0.24 | 0.92 | 0.6189 |
|  |  | 2020-05-13 | 0.14 | 0.17 | 0.027 | 0.11 | 0.22 | 0.85 | 0.3545 |
|  |  | 2020-05-20 | 0.13 | 0.16 | 0.028 | 0.10 | 0.21 | 0.86 | 0.4458 |
|  |  | 2020-05-27 | 0.15 | 0.20 | 0.027 | 0.14 | 0.25 | 0.79 | 0.1426 |
|  |  | 2020-06-03 | 0.15 | 0.16 | 0.027 | 0.11 | 0.21 | 0.95 | 0.7786 |
|  |  | 2020-06-10 | 0.19 | 0.18 | 0.027 | 0.12 | 0.23 | 1.05 | 0.7504 |
|  |  | 2020-06-17 | 0.14 | 0.14 | 0.027 | 0.090 | 0.20 | 0.98 | 0.8959 |
|  |  | 2020-06-24 | 0.11 | 0.17 | 0.027 | 0.11 | 0.22 | 0.64 | 0.0287 |
|  |  | 2020-07-01 | 0.15 | 0.14 | 0.027 | 0.091 | 0.20 | 1.06 | 0.7405 |
|  |  | 2020-07-08 | 0.17 | 0.14 | 0.027 | 0.085 | 0.19 | 1.25 | 0.2092 |
|  |  | 2020-07-15 | 0.15 | 0.13 | 0.027 | 0.079 | 0.19 | 1.11 | 0.6067 |
|  |  | 2020-07-22 | 0.14 | 0.14 | 0.027 | 0.087 | 0.19 | 0.97 | 0.8825 |
|  |  | 2020-07-29 | 0.14 | 0.14 | 0.027 | 0.083 | 0.19 | 1.02 | 0.9166 |
|  |  | 2020-08-05 | 0.15 | 0.15 | 0.027 | 0.094 | 0.20 | 0.99 | 0.9414 |
|  |  | 2020-08-12 | 0.15 | 0.17 | 0.027 | 0.12 | 0.23 | 0.87 | 0.4096 |
|  |  | 2020-08-19 | 0.16 | 0.18 | 0.027 | 0.12 | 0.23 | 0.88 | 0.4422 |
|  |  | 2020-08-26 | 0.15 | 0.19 | 0.027 | 0.14 | 0.24 | 0.81 | 0.1787 |
|  |  | 2020-09-02 | 0.16 | 0.17 | 0.027 | 0.12 | 0.22 | 0.92 | 0.6045 |
|  |  | 2020-09-09 | 0.14 | 0.16 | 0.027 | 0.11 | 0.22 | 0.85 | 0.3719 |
|  |  | 2020-09-16 | 0.16 | 0.17 | 0.027 | 0.12 | 0.23 | 0.94 | 0.6838 |
|  |  | 2020-09-23 | 0.15 | 0.18 | 0.027 | 0.12 | 0.23 | 0.86 | 0.3506 |
|  |  | 2020-09-30 | 0.19 | 0.17 | 0.027 | 0.11 | 0.22 | 1.17 | 0.2979 |
|  |  | 2020-10-07 | 0.18 | 0.19 | 0.027 | 0.13 | 0.24 | 0.95 | 0.7121 |
|  |  | 2020-10-14 | 0.17 | 0.20 | 0.027 | 0.14 | 0.25 | 0.87 | 0.3303 |
|  |  | 2020-10-21 | 0.27 | 0.18 | 0.027 | 0.13 | 0.23 | 1.50 | 0.0012 |
|  |  | 2020-10-28 | 0.15 | 0.16 | 0.027 | 0.11 | 0.22 | 0.96 | 0.7971 |
|  |  | 2020-11-04 | 0.21 | 0.17 | 0.027 | 0.11 | 0.22 | 1.26 | 0.1203 |
|  |  | 2020-11-11 | 0.19 | 0.19 | 0.027 | 0.13 | 0.24 | 1.01 | 0.9702 |
|  |  | 2020-11-18 | 0.16 | 0.18 | 0.027 | 0.12 | 0.23 | 0.93 | 0.6321 |
|  |  | 2020-11-25 | 0.19 | 0.19 | 0.027 | 0.14 | 0.25 | 1.00 | 0.9918 |
|  |  | 2020-12-02 | 0.20 | 0.20 | 0.027 | 0.15 | 0.25 | 0.99 | 0.9379 |
|  |  | 2020-12-09 | 0.24 | 0.23 | 0.027 | 0.18 | 0.29 | 1.04 | 0.7610 |
|  |  | 2020-12-16 | 0.27 | 0.23 | 0.028 | 0.18 | 0.29 | 1.16 | 0.1717 |
|  |  | 2020-12-23 | 0.16 | 0.12 | 0.027 | 0.070 | 0.18 | 1.30 | 0.1712 |
| P02 | Anthelmintics | 2020-01-01 | 0 | 0 | 0.0028 | -0 | 0.0060 | 0.00 | 0.8300 |
|  |  | 2020-01-08 | 0.0014 | -0 | 0.0027 | -0 | 0.0052 | . | . |
|  |  | 2020-01-15 | 0 | 0 | 0.0027 | -0 | 0.0056 | 1.71 | 0.9399 |
|  |  | 2020-01-22 | 0 | -0 | 0.0027 | -0 | 0.0052 | . | . |
|  |  | 2020-01-29 | 0 | 0.0019 | 0.0027 | -0 | 0.0072 | 0.35 | 0.6422 |
|  |  | 2020-02-05 | 0 | -0 | 0.0027 | -0 | 0.0051 | . | . |
|  |  | 2020-02-12 | 0 | -0 | 0.0027 | -0 | 0.0053 | . | . |
|  |  | 2020-02-19 | 0.0018 | -0 | 0.0027 | -0 | 0.0050 | . | . |
|  |  | 2020-02-26 | 0 | -0 | 0.0027 | -0 | 0.0050 | . | . |
|  |  | 2020-03-04 | 0 | 0 | 0.0027 | -0 | 0.0058 | 0.75 | 0.9621 |
|  |  | 2020-03-11 | 0 | 0 | 0.0028 | -0 | 0.0060 | 0.76 | 0.9650 |
|  |  | 2020-03-18 | 0 | 0 | 0.0028 | -0 | 0.0056 | 0.00 | 0.9676 |
|  |  | 2020-03-25 | 0 | -0 | 0.0029 | -0 | 0.0053 | . | . |
|  |  | 2020-04-01 | 0 | 0 | 0.0029 | -0 | 0.0062 | 1.40 | 0.9391 |
|  |  | 2020-04-08 | 0 | -0 | 0.0031 | -0 | 0.0049 | . | . |
|  |  | 2020-04-15 | 0 | 0.0028 | 0.0027 | -0 | 0.0081 | 0.00 | 0.2964 |
|  |  | 2020-04-22 | 0 | 0 | 0.0027 | -0 | 0.0061 | 0.60 | 0.9059 |
|  |  | 2020-04-29 | 0 | 0 | 0.0027 | -0 | 0.0057 | 0.81 | 0.9797 |
|  |  | 2020-05-06 | 0 | 0 | 0.0027 | -0 | 0.0059 | 0.16 | 0.8506 |
|  |  | 2020-05-20 | 0 | 0.0043 | 0.0027 | -0 | 0.0096 | 0.00 | 0.1140 |
|  |  | 2020-05-27 | 0 | 0 | 0.0027 | -0 | 0.0062 | 0.32 | 0.8196 |
|  |  | 2020-06-03 | 0 | -0 | 0.0027 | -0 | 0.0052 | . | . |
|  |  | 2020-06-10 | 0 | 0.0047 | 0.0027 | -0 | 0.0100 | 0.00 | 0.0813 |
|  |  | 2020-06-17 | 0 | 0 | 0.0027 | -0 | 0.0056 | 0.00 | 0.9126 |
|  |  | 2020-06-24 | 0 | 0 | 0.0027 | -0 | 0.0059 | 0.00 | 0.8185 |
|  |  | 2020-07-01 | 0 | 0.0021 | 0.0027 | -0 | 0.0075 | 0.00 | 0.4439 |
|  |  | 2020-07-15 | 0 | -0 | 0.0027 | -0 | 0.0048 | . | . |
|  |  | 2020-07-29 | 0 | -0 | 0.0027 | -0 | 0.0051 | . | . |
|  |  | 2020-08-05 | 0 | 0 | 0.0027 | -0 | 0.0058 | 0.00 | 0.8511 |
|  |  | 2020-08-12 | 0 | 0 | 0.0027 | -0 | 0.0054 | 3.46 | 0.8778 |
|  |  | 2020-08-19 | 0 | -0 | 0.0027 | -0 | 0.0050 | . | . |
|  |  | 2020-08-26 | 0 | -0 | 0.0027 | -0 | 0.0048 | . | . |
|  |  | 2020-09-02 | 0 | 0 | 0.0027 | -0 | 0.0055 | 0.00 | 0.9752 |
|  |  | 2020-09-09 | 0 | -0 | 0.0027 | -0 | 0.0052 | . | . |
|  |  | 2020-09-16 | 0 | 0.0026 | 0.0027 | -0 | 0.0080 | 0.11 | 0.3967 |
|  |  | 2020-09-23 | 0 | 0.0022 | 0.0027 | -0 | 0.0075 | 0.13 | 0.4716 |
|  |  | 2020-09-30 | 0 | -0 | 0.0027 | -0 | 0.0051 | . | . |
|  |  | 2020-10-07 | 0 | 0 | 0.0027 | -0 | 0.0054 | 1.79 | 0.9620 |
|  |  | 2020-10-14 | 0 | -0 | 0.0027 | -0 | 0.0051 | . | . |
|  |  | 2020-10-21 | 0 | 0 | 0.0027 | -0 | 0.0054 | 0.00 | 0.9724 |
|  |  | 2020-10-28 | 0.0012 | -0 | 0.0027 | -0 | 0.0051 | . | . |
|  |  | 2020-11-04 | 0 | -0 | 0.0027 | -0 | 0.0052 | . | . |
|  |  | 2020-11-11 | 0 | -0 | 0.0027 | -0 | 0.0049 | . | . |
|  |  | 2020-11-18 | 0 | -0 | 0.0027 | -0 | 0.0052 | . | . |
|  |  | 2020-11-25 | 0 | 0 | 0.0027 | -0 | 0.0056 | 0.00 | 0.9010 |
|  |  | 2020-12-02 | 0 | -0 | 0.0027 | -0 | 0.0049 | . | . |
|  |  | 2020-12-09 | 0 | 0 | 0.0027 | -0 | 0.0055 | 0.72 | 0.9778 |
|  |  | 2020-12-16 | 0 | 0 | 0.0027 | -0 | 0.0061 | 0.38 | 0.8609 |
|  |  |  |  |  |  |  |  |  |  |

*Abbreviations: Defined daily dose (DDD), Confidence limit (CL)*, *Anatomical Therapeutic Code (ATC)*

**p-values are presented uncorrected for multiple comparisons*
