# Supplementary material for: Antimicrobial use in Sweden during the COVID-19 pandemic: prescription fill and inpatient care requisition patterns
Source: BMC Infect Dis. 2022 May 24;22:492. doi: 10.1186/s12879-022-07405-3 (PMC9128331; doi:10.1186/s12879-022-07405-3)
Supplement: Supplementary file 2 — Additional file 2: Figure S1. Weekly number of prescriptions filled per 1000 inhabitants for selected ATC therapeutic subgroups, by gender, Sweden, 2015-2020 Legend: Yellow-2015;Blue-2016; Red-2017; Green-2018; Orange-2019; Black-2020. Note that the scales on the y-axes are different and not directly comparable. *Excluding J01XX. Figure S2. Weekly number of prescriptions filled per 1000 inhabitants for selected ATC therapeutic subgroups, by geographical area, Sweden, 2015-2020. Legend: Yellow-2015; Blue-2016; Red-2017; Green-2018; Orange-2019; Black-2020. Note that the scales on the y-axes are different and not directly comparable. *Excluding J01XX. Figure S3. Observed versus predicted weekly number of prescriptions filled per 1000 inhabitants with 95% CL for selected antimicrobials by ATC therapeutic subgroup, Sweden, 2020. Note that the scales on the y-axes are different and not directly comparable. Legend: Black–observed; Blue dashed dotted–predicted; Blue dashed-95% confidence limits. Values that remained significant after Bonferroni correction are marked as follows: Single red circle– p≤0.05; Double red circle–p≤0.01;Red star-p≤0.001. Figure S4. Observed versus predicted weekly number of prescriptions filled per 1000 inhabitants with 95% CL for other antimicrobials by ATC chemical subgroups, Sweden, 2020. Note that the scales on they-axes are different and not directly comparable. Legend: Black-observed; Blue dashed dotted-predicted; Blue dashed-95% confidence limits. Values that remained significant after Bonferroni correction are marked as follows: p≤0.05 – single red circle;p≤0.01 – double red circle;p≤0.001 - red star. [file 12879_2022_7405_MOESM2_ESM.docx]

Figure S1 Weekly number of prescriptions filled per 1000 inhabitants for selected ATC therapeutic subgroups, by gender, Sweden, 2015-2020

*Legend: Yellow-2015; Blue-2016; Red-2017; Green-2018; Orange-2019; Black-2020*

*Note that the scales on the y-axes are different and not directly comparable.*

*** *Excluding J01XX*

Figure S2 Weekly number of prescriptions filled per 1000 inhabitants for selected ATC therapeutic subgroups, by geographical area, Sweden, 2015-2020

*Legend: Yellow-2015; Blue-2016; Red-2017; Green-2018; Orange-2019; Black-2020*

*Note that the scales on the y-axes are different and not directly comparable.*

*** *Excluding J01XX*

Figure S3 Observed versus predicted weekly number of prescriptions filled per 1000 inhabitants with 95% CL for selected antimicrobials by ATC therapeutic subgroup, Sweden, 2020

*Note that the scales on the y-axes are different and not directly comparable.*

*Legend: Black–observed; Blue dashed–predicted; Blue dashed dotted-95% confidence limits*

*Values that remained significant after Bonferroni correction are marked as follows: Single red circle– p≤0.05; Double red circle–p≤0.01; Red star-p≤0.001.*

Figure S3 Observed (black) versus predicted (dashed blue) weekly number of prescriptions filled per 1000 inhabitants with 95% CL (dashed dotted blue) for other antimicrobials by ATC chemical subgroups, Sweden, 2020

*Note that the scales on the y-axes are different and not directly comparable. Values that remained significant after Bonferroni correction are marked as follows: p≤0.05 – single red circle; p≤0.01 – double red circle; p≤0.001 - red star.*
